# Synthesis of multi-module low density lipoprotein receptor class A domains with acid labile cyanopyridiniumylides (CyPY) as aspartic acid masking groups†

**DOI:** 10.1039/d2cb00234e

**Published:** 2023-01-24

**Authors:** Kevin Neumann, Alex Vujinovic, Saidu Kamara, André Zwicky, Simon Baldauf, Jeffrey W. Bode

**Affiliations:** a Laboratorium für Organische Chemie, Department of Chemistry and Applied Biosciences, ETH Zürich Zürich 8093 Switzerland bode@org.chem.ethz.ch

## Abstract

Low-density lipoprotein receptor class A domains (LA modules) are common ligand-binding domains of transmembrane receptors of approximately 40 amino acids that are involved in several cellular processes including endocytosis of extracellular targets. Due to their wide-ranging function and distribution among different transmembrane receptors, LA modules are of high interest for therapeutic applications. However, the efficient chemical synthesis of LA modules and derivatives is hindered by complications, many arising from the high abundance of aspartic acid and consequent aspartimide formation. Here, we report a robust, efficient and general applicable chemical synthesis route for accessing such LA modules, demonstrated by the synthesis and folding of the LA3 and LA4 modules of the low-density lipoprotein receptor, as well as a heterodimeric LA3–LA4 constructed by chemical ligation. The synthesis of the aspartic acid-rich LA domain peptides is made possible by the use of cyanopyridiniumylides (CyPY) – reported here for the first time – as a masking group for carboxylic acids. We show that cyanopyridiniumylide masked aspartic acid monomers are readily available building blocks for solid phase peptide synthesis and successfully suppress aspartimide formation. Unlike previously reported ylide-based carboxylic acid protecting groups, CyPY protected aspartic acids are converted to the free carboxylic acid by acidic hydrolysis and are compatible with all common residues and protecting groups. The chemical synthesis of Cys- and Asp-rich LA modules enables new access to a class of difficult to provide, but promising protein domains.

## Abstract 2

Single and multi-module low density lipoprotein receptor class A domains (LA modules) were synthesized with new acid labile cyanopyridiniumylides (CyPY) as aspartic acid monomers, which do not form aspartimides during solid phase peptide synthesis.

## Graphical abstract

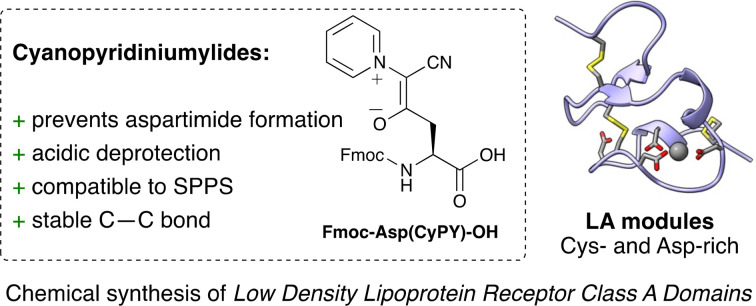

## Introduction

The low-density lipoprotein receptor (LDLR) family comprises a large range of transmembrane receptors responsible for endocytosis of several extracellular ligands.^1^ LDLR, the prototype of this receptor family, maintains the plasma level of cholesterol by binding to and subsequent endocytosis of cholesterol-rich low-density lipoproteins (LDL). Besides their endocytosis function, it is well accepted that the receptors are involved in other cellular processes including cell signaling, cell migration and vitamin homeostasis.^2^

**Fig. 1:**
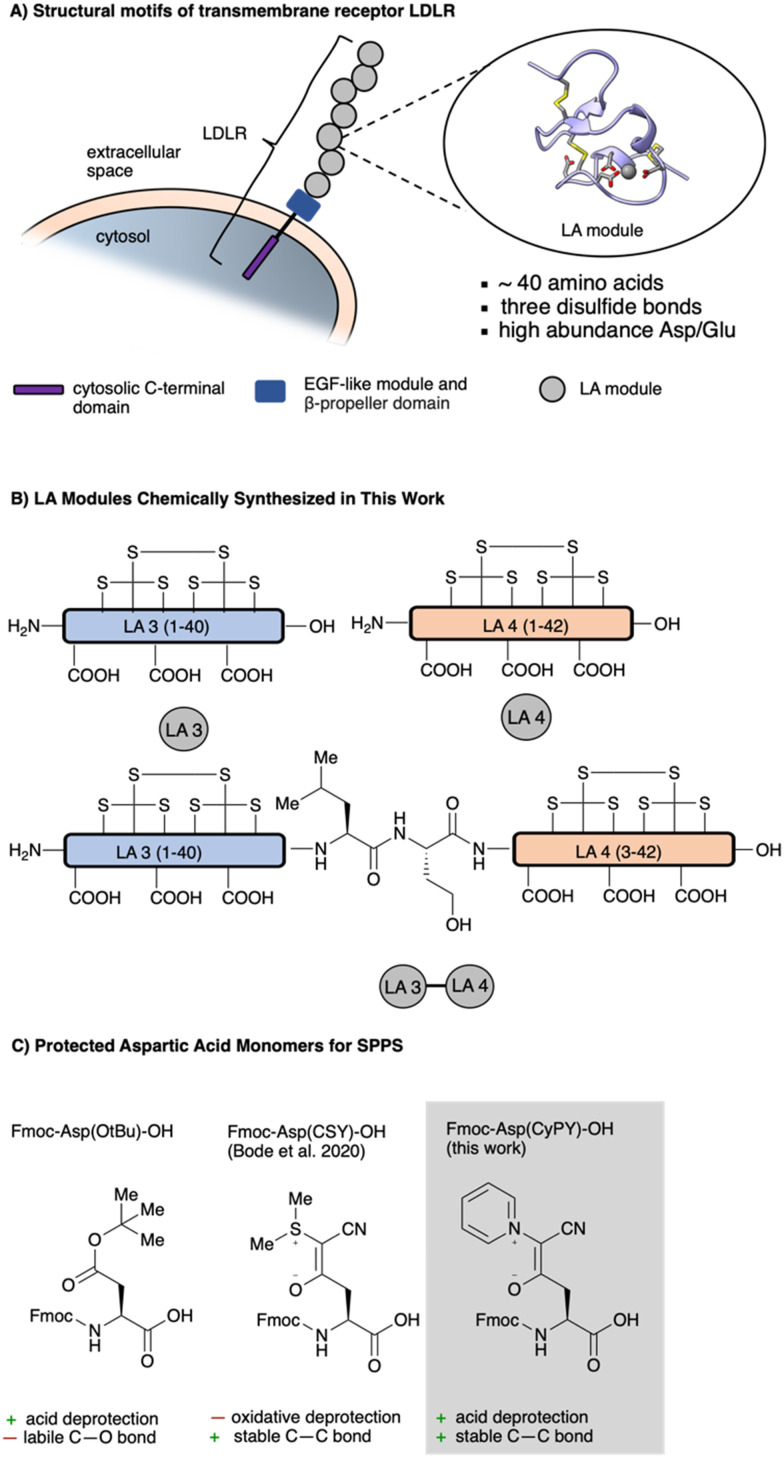
(A) Low density lipoprotein receptor (LDLR) consists of a cytosolic domain, EGF-like domain, β-propeller domain and several LA modules (LA module reproduced from PDB 1AJJ, ref. 32). (B) Overview of LA modules that were chemically synthesized in this work. (C) Comparison of ylide masked aspartic acid building blocks with the typically used aspartic acid building block Fmoc-Asp(O^*t*^Bu)-OH.

A common structural motif of LDLR-associated receptors is the N-terminal extracellular concatenation of so-called LDLR class A domains (LA modules).^3,4^ These protein microdomains are responsible for high affinity ligand binding. The number of concatenations of LA modules varies over different receptors from seven to several dozens. Typically, each LA module consists of ∼40 amino acids with three disulfide bonds aligned in a tandem pattern and an octahedral calcium-binding site formed by highly conserved acidic amino acid residues, in particular aspartic acid (Fig. 1A). In addition to calcium binding, the negatively charged pocket is also responsible for electrostatic binding to basic residues in their ligands, most commonly lysines.^5^

LA modules are not only found as domains of LDLR and LDLR-related receptors but are also present in other transmembrane receptors including low-density lipoprotein receptor class A domain-containing protein 3 (LDLRAD3) and relaxin receptor 1 (RXFP1), which are responsible for the uptake of Venezuelan equine encephalitis virus and relaxins.^6,7^ Due to their versatile functions and association to many diseases, LA modules are of high interest for therapeutic applications, either as therapeutic targets or as therapeutics themselves.^8,9^ A versatile and robust chemical synthesis route of LA modules would advance their therapeutic potential and enhance comprehension of their role in cellular function.

In this report, we document the synthesis of two single LA modules and the construction of a synthetic, multi-domain LA-module with KAHA ligation (Fig. 1B). The cysteine- and aspartic acid-rich LA modules are often extremely difficult to synthesize due to the high density of cysteine residues and the formation of undesired aspartimides. Recently, we reported the use of cyanosulfurylides as masking groups for carboxylic acids on aspartic acid side chains (Fig. 1). These remarkably stable zwitterionic protecting groups effectively prevented aspartimide formation and enabled the synthesis of otherwise inaccessible peptides but required stochiometric amounts of electrophilic halogen species for final deprotection of the Asp residues.^10,11^ This oxidative reaction necessitated a separate step after standard resin cleavage and global deprotection and was incompatible with methionine residues and the most common cysteine protecting groups. Therefore, although this solution to aspartimide formation is appropriate for many challenging peptides, it is not suitable for the synthesis of complex dimeric LA modules by convergent assembly using native chemical ligation (NCL) and/or the *alpha*-ketoacid–hydroxylamine (KAHA) ligation.^12,13^ The synthesis and ligation of these modules therefore required the development of a new class of aspartimide-detering protecting groups – ideally one that could be removed under aqueous, acidic conditions. These efforts led to the identification of cyanopyridiniumylides (CyPYs), reported here for the first time, as easily installed and removed protecting groups for Asp monomers, resulting in straightforward access to folded LA domains by SPPS and their heterodimers by chemical ligation.

## Results

### Cyanopyridiumylides as masking groups for carboxylic acids

**Scheme 1:**
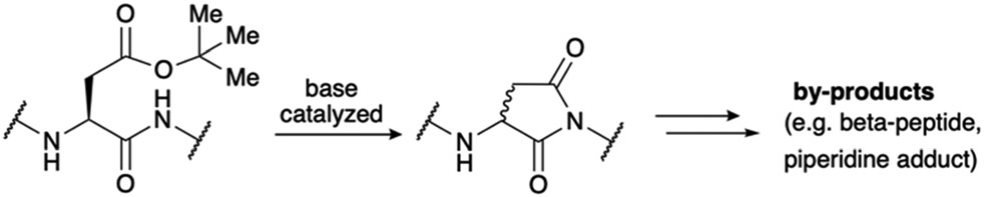
Aspartimide is formed by the base-catalyzed attack of the backbone amide onto the ester protected aspartic acid sidechain. Several by-products are formed upon ring-opening by nucleophiles such as water and piperidine.

At the outset of our studies, we intended to chemically synthesize LA modules 3 and 4 of LDLR, as these modules have been reported to contribute significantly to strong ligand binding.^14^ Initial attempts to prepare modules LA3 or LA4 of LDLR were not successful using the standard pool of peptide building blocks. This was in accordance with both our previous experience and literature reports indicating that many LA modules are extremely difficult to synthesize or even synthetically inaccessible.^15^ The high abundance of aspartic acid results in motifs prone to aspartimide formation, a frequently occurring problem for longer peptides during solid phase peptide synthesis (SPPS).^16^ Aspartimides are formed by the attack of the amide backbone onto the protected side chain of aspartic acid, which is typically an ester (Scheme 1).^17^ Aspartimide, a succinimide derivative prone to racemization and ring opening, results in a range of undesired side products, including diastereomers and regioisomers. As it is promoted by base, the formation of aspartimide can occur repeatedly during the iterative cycles of SPPS.

**Fig. 2:**
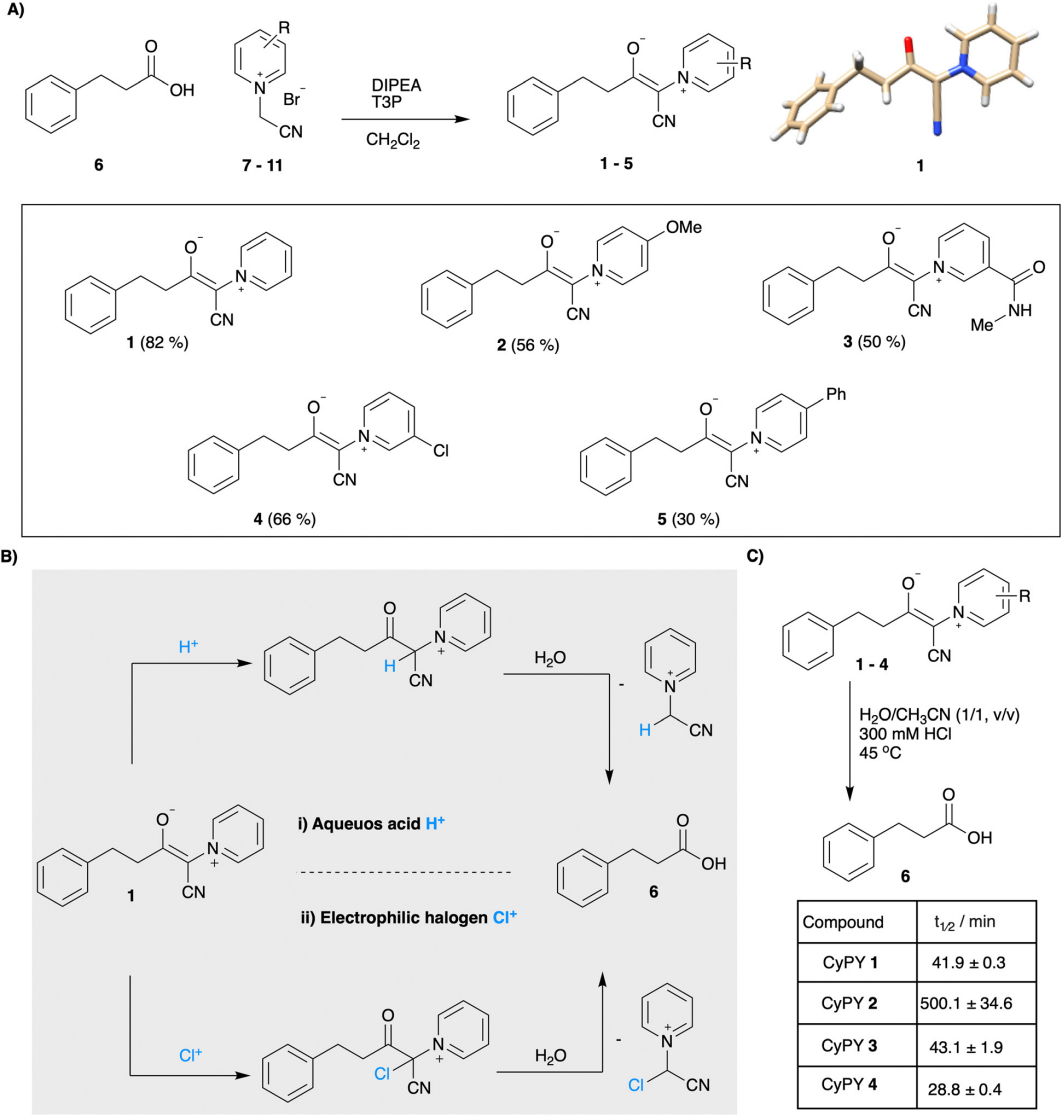
(A) Synthesis of cyanopyridiniumylides 1–5 from hydrocinnamic acid 6 and pyridinium salt 7–11 (7 R = H, 8 = 4-OCH3, 9 = C(O)NHCH3, 10 = 3-Cl, 11 = 4-Ph). The crystal structure of cyanopyridiniumylide 1 confirms Z configuration. (B) Postulated mechanism for the conversion of cyanopyridiniumylides to the free carboxylic acid using (i) acidic conditions (ii) electrophilic halogen species. (C) Reaction rates of hydrolysis of 1–4 under acidic conditions as determined by 1H-NMR. 4-Phenyl substituted cyanopyridiniumylide 5 was insoluble under the given conditions.

While our recently reported cyanosulfurylides could suppress aspartimide formation during SPPS, their conversion to aspartic acid residues was not compatible with all peptide side chains – including methionine and key functional groups employed for the *alpha*-ketoacid–hydroxylamine (KAHA) peptide ligation. We therefore sought to identify alternative stabilized ylides that could be converted to the free carboxylic acid under non-oxidative conditions. By considering the postulated mechanism of cyanosulfurylide deprotection, we envisioned that protonation of a somewhat more basic ylide could replace the chlorination step, leading to an electrophilic ketone that could undergo a haloform-like decomposition to the carboxylic acid in the presence of water. After considering potential sulfur, phosphorous, and nitrogen-based alternatives, we selected cyanopyridiniumylides (CyPY) (Fig. 1 and 2) for further development.^18^ We found that CyPYs display similar chemical reactivity to cyanosulfurylides, but can be electronically tuned by substituent groups on the aromatic ring.^19,20^ We were further encouraged by early reports from Taylor implying that cyanopyridiniumylides can be converted to free carboxylic acids by treatment with acid.^21^

While pyridinium ylides are commonly employed as building blocks in organic chemistry, the more stabilized CyPYs have received less attention, likely due to their relatively low reactivity.^22^ In order to investigate their stability and ability to hydrolyze under acidic conditions, we synthesized model cyanopyridiniumylides 1–5 with varied electronic properties from hydrocinnamic acid 6 and readily available pyridinium salts 7–11 (Fig. 2a and ESI†). X-Ray structural analysis of a single crystal of 1 confirms Z-configuration and suggests conjugation to the aromatic pyridinium resulting in a planar structure.

NMR studies revealed that under aqueous acidic conditions CyPY 1 hydrolyzes to hydrocinnamic acid 6 and the corresponding pyridinium salt 7. We hypothesize that protonation in the *alpha*-position occurs upon incubation under strong acidic conditions, followed by hydration and elimination of a pyridinium ylide (Fig. 2B). We evaluated the stability of the different cyanopyridiniumylides by ^1^H-NMR and were pleased to observe that all cyanopyridiniumylides display high stability under neutral and basic conditions, as well as in the presence of acetic acid anhydride (ESI† page 7). Notably, the stability of cyanopyridiniumylides under acidic conditions was influenced by the electronic properties of the pyridine moiety; electron-rich CyPY-OMe 2 displayed the highest stability under acidic conditions (300 mM HCl, 45 °C) with hydrolysis half-life of 500.1 ± 34.6 min. Unsubstituted CyPY 1 and CyPY-amide 3 (*t*_1/2_ = 41.3 ± 0.6 min and 43.1 ± 1.9 min, respectively) as well as electron-deficient CyPY-Cl 4 (28.8 ± 0.4 min) hydrolyze significantly faster under acidic conditions.

We found that aqueous acidic conditions including dilute hydrogen chloride (350 mM) and H_2_O/AcOH (4 : 1 v : v, ESI† page 10) promote hydrolysis of cyanopyridiniumylides. We therefore sought adaptations to standard SPPS workflows for global deprotection and resin cleavage that would also result in deprotection of Asp-CyPY and directly afford fully unprotected peptides – *i.e.* without the additional manipulations required for removing the sulfurylide counterparts.

### Cyanopyridiniumylides as building blocks in peptide chemistry

Based on the stability and reactivity of these cyanopyridiniumylides, we selected unsubstituted cyanopyridiniumylide 1 for construction of a new aspartic acid building block for SPPS. CyPY-protected l-aspartic acid 12 was accessed from commercially available Fmoc-Asp(OH)-O^*t*^Bu 14 and pyridinium salt 7 in good yield (70%). The acid-labile nature of the CyPY building block required optimization of the *tert*-butyl ester deprotection to avoid premature cleavage of the side chain cyanopyridiniumylide. This was accomplished with anhydrous TFA in CH_2_Cl_2_ (1/1, v/v), followed by an aqueous workup featuring basic and weakly acidic washes to provide aspartic acid monomer 12.

**Fig. 3:**
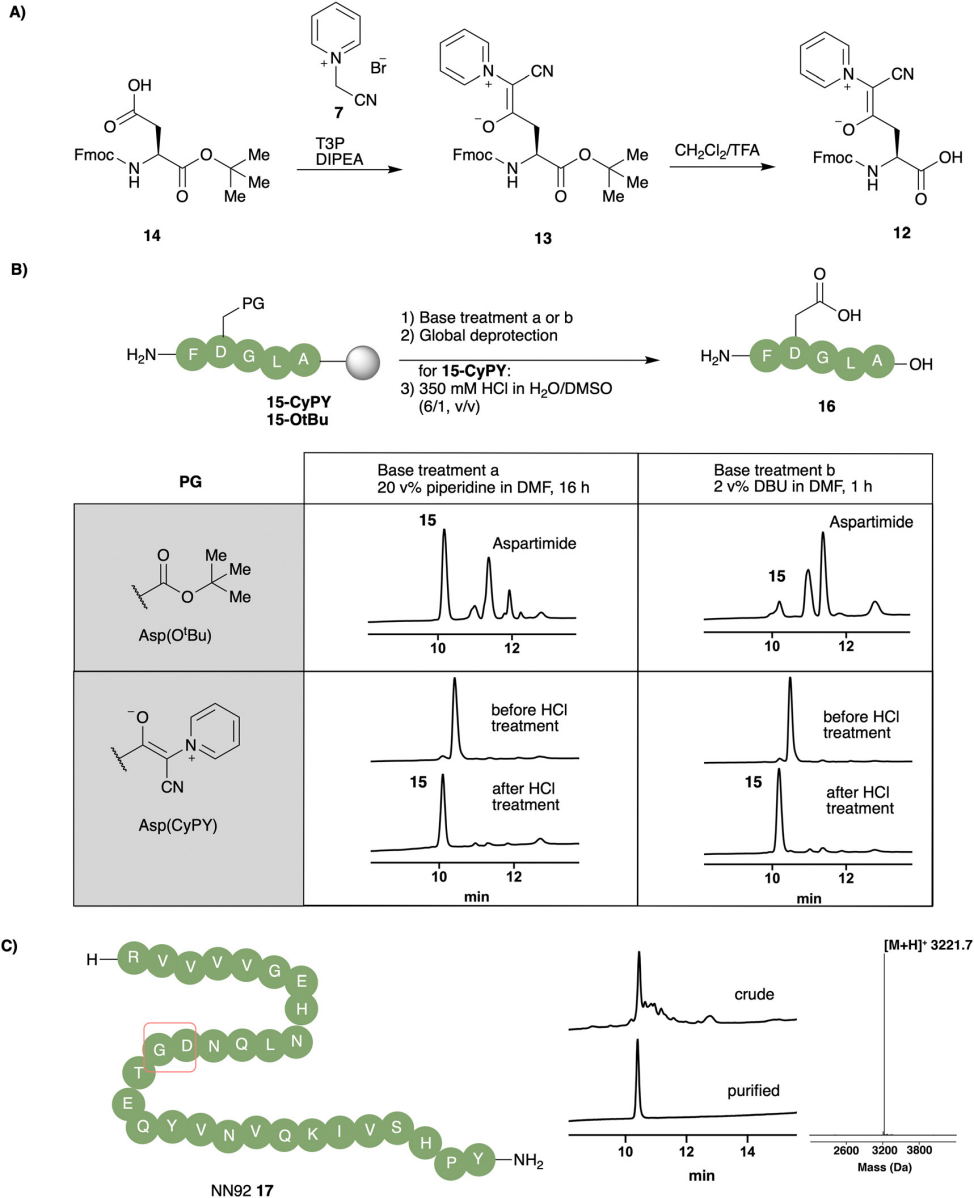
(A) Synthesis of Fmoc-Asp(CyPY)-OH 12 from commercially available Fmoc-Asp(OH)-O^*t*^Bu 14 and pyridinium salt 7. (B) Model peptide 16 synthesized using conventional building block Fmoc-Asp(O^*t*^Bu)-OH and Fmoc-Asp(CyPY)-OH 12. 15-CyPY and 15-O^*t*^Bu were incubated with (a) 20 v% piperidine in DMF (16 h, rt) or (b) 2 v% DBU (1 h, rt) to evaluate their ability to prevent formation of aspartimide (15-Aspartimide). (C) Sequence of aspartimide prone NN92 17 containing an Asp-Gly motif.

To determine the ability of CyPY to suppress aspartimide formation, we synthesized model peptides 15-CyPY and 15-O^*t*^Bu using Fmoc-Asp(CyPY)-OH (12) and Fmoc-Asp(O^*t*^Bu)-OH, respectively, and treated those peptides, on resin, with bases typically used in peptide chemistry (piperidine and DBU). After standard global deprotection, peptides were incubated in 350 mM HCl in H_2_O/DMSO (6 : 1, v : v) to ensure full hydrolysis of CyPY moieties. HPLC analysis revealed that no significant amount of aspartimide was observed for model peptide 16, prepared by using CyPY building block 12, even after prolonged treatment with piperidine (16 h) or DBU (1 h). In contrast, peptide 16 prepared using the conventional *tert*-butyl ester protected aspartic acid monomer showed a large amount of aspartimide and aspartimide-related side-products (Fig. 3B). We attributed the small amounts of aspartimide observed with the CyPY group to the acid mediated hydrolysis of CyPY, since its formation was independent of the exposure time to base and depended on solvent system (Fig. 3B and ESI†). In brief, to minimize aspartimide formation during CyPY-removal, best results were observed by using high proportions of water instead of organic solvent; in case a co-solvent is needed, DMSO provides better results than CH_3_CN.

To demonstrate the compatibility of Fmoc-Asp(CyPY)-OH 12 with standard-SPPS protocols, we successfully employed it for the synthesis of peptide S2, which contained oxidation-sensitive amino acid residues including methionine, trityl protected cysteine, tyrosine and tryptophan (see ESI†).

We applied Fmoc-Asp(CyPY)-OH 12 for the synthesis of NN92 17, a 29 amino acids long peptide previously reported as prone to aspartimide formation.^23^ Using Fmoc-Asp(CyPY)-OH 12, we obtained the desired peptide in high purity, with only a minor amount of aspartimide being observed (Fig. 3C). Pure NN92 17 was isolated after HPLC purification in good yields (9%).

### Cyanopyridiniumylides for the synthesis of monomeric and dimeric LA-modules

**Fig. 4:**
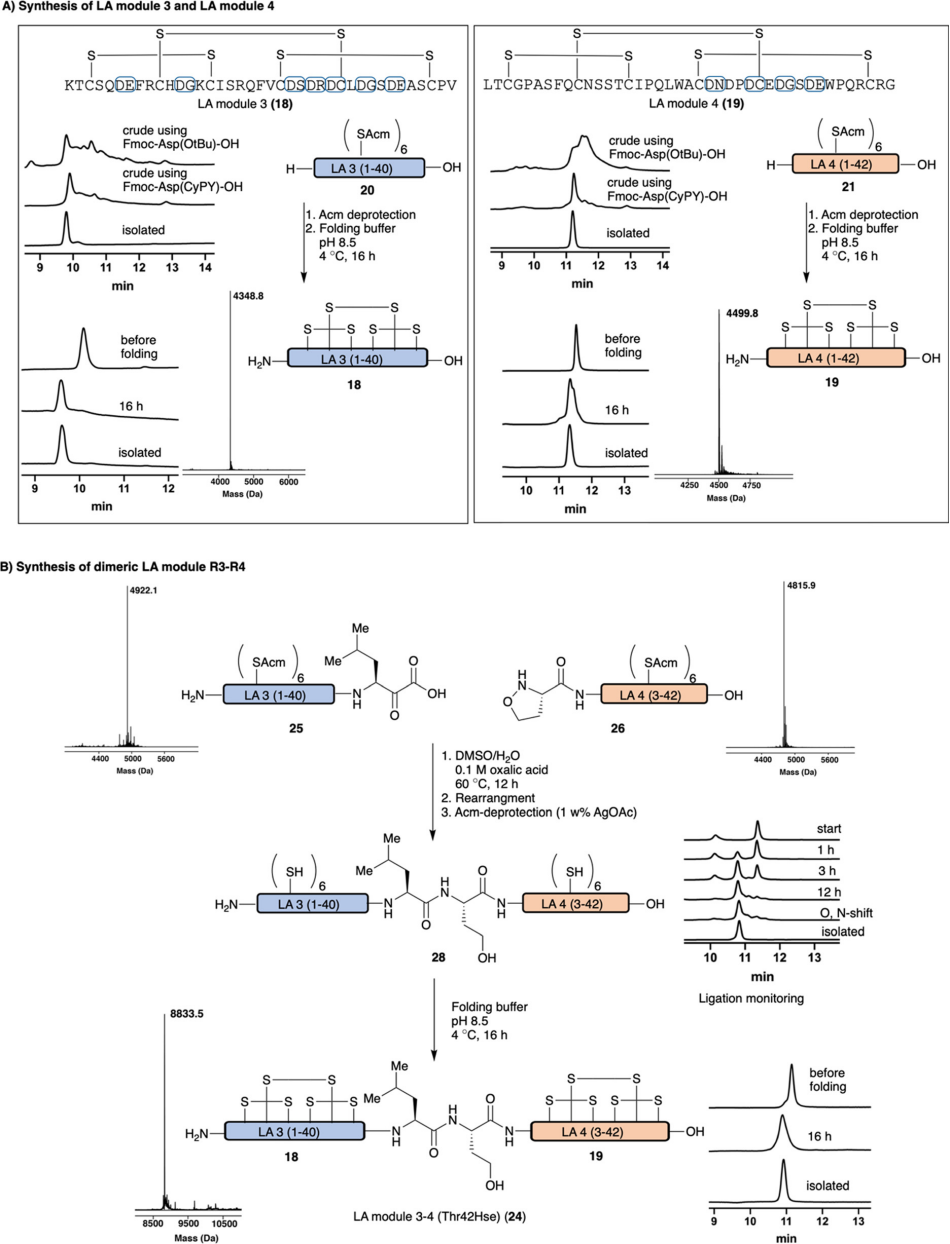
(A) Synthesis of monomeric LA modules R3 18 and R4 19. Aspartic acids residues able of aspartimide formation are highlighted by blue circles. Synthesis utilizing Fmoc-Asp(CyPY)-OH 12 and Fmoc-Asp(O^*t*^Bu)-OH allowed comparison of the two monomers. (B) Synthesis of dimeric LA module R3–R4 (Thr42Hse) 24 using KAHA ligation. Acm-deprotection conditions: AcOH/H_2_O (1/1, v/v), 1 w% AgOAc, 45 °C, 2 h; Folding conditions: NaCl (150 mM), CaCl_2_ (2.5 mM), TRIS (50 mM), GSH (3 mM), GSSG (0.3 mM), pH 8.5, 4 °C, 16 h.

With a successful protecting group and acid cleavage protocol established, we turned our attention to our intended target, LA-modules from LDLR. We envisioned that Fmoc-Asp(CyPY)-OH 12 would allow for the chemical synthesis of LA modules, not only as single domains but also more complex heterodimeric structures. We targeted the synthesis of two monomeric LA modules, LA module R3 18 and LA module R4 19 of LDLR consisting out of 40 and 42 amino acid residues, respectively. Both modules contain several highly aspartimide-prone motifs (*e.g.* Asp-Asn, Asp-Gly, Asp-Cys) and six cysteine residues that form three intramolecular disulfide bonds. We performed the synthesis with Fmoc-Asp(CyPY)-OH 12 using otherwise standard peptide synthesis protocols, including Acm protected Cys, which were not compatible with cyanosulfurylide protected Asp. A parallel synthesis was carried out using conventional Fmoc-Asp(O^*t*^Bu)-OH, which was treated under the same conditions. After full elongation of the peptides and global deprotection, the crude peptides were incubated in acidic H_2_O/DMSO mixtures in order to convert the cyanopyridiniumylides to free carboxylic acids. When Fmoc-Asp(CyPY)-OH 12 was utilized, we observed the desired, fully elongated Acm-protected LA module R3 20 and LA module R4 21 as the major products, as determined by HPLC (Fig. 4A and ESI†). In particular, Acm-protected LA module R4 21 was obtained in good yield, and only minor amounts of by-products were observed. The Acm-protected cysteine residues were quantitatively deprotected using AgOAc in AcOH/H_2_O affording unfolded peptides R3 22 and R4 23. The peptides were folded in the presence of calcium cations by formation of three disulfide bonds, and isolated by HPLC purification to provide pure LA module R3 18 and LA module R4 19. While we carried out the Acm-removal and folding separately, one can consider combining these steps in the future.^24,25^

LA modules often exist as linear heterogenic oligomers and we sought to prepare a dimeric LA module, namely LA dimer R3–R4 of LDLR 24, by the union of the two single monomeric LA modules to afford a folded peptide consisting of ∼80 AA residues and 6 disulfide bonds. In the past, the *alpha*-ketoacid-hydroxylamine (KAHA) ligation was successfully utilized for the chemical synthesis of a range of different proteins and was selected for this purpose.^12^ We prepared LA module R3 25 and LA module R4 26 with a C-terminal *alpha*-ketoacid and a N-terminal (*S*)-5-oxaproline, respectively, using Fmoc-Asp(CyPY)-OH 12. After peptide elongation and global deprotection, the unpurified peptides were incubated in acidic H_2_O/DMSO (4/1, v/v) to promote conversion of cyanopyridiniumylides to the free carboxylic acids, which were purified by HPLC. KAHA ligation of purified LA module R3 25 and LA module R4 26 was carried out in DMSO/H_2_O (9/1, v/v) with 0.1 M oxalic acid. The initially formed *depsi*-peptide was rearranged to amide 27 in sodium carbonate buffer (pH 9.5) for 1 h and purified by HPLC. Acm deprotection of cysteines afforded the free thiol containing dimeric LA module R3–R4 (Thr42Hse) 28. After some optimization, the dimeric module – containing 12 unprotected Cys residues – was folded using conditions similar to those identified for the monomeric modules (ESI† page 16), resulting in the folded protein domains containing 6 disulfide bonds. The pure dimeric LA module R3–R4 (Thr42Hse) module 24 was obtained after HPLC purification.

## Discussion

Aspartimide formation is widely recognized as one of the most difficult complications of solid phase peptide synthesis and can severely limit access to certain peptides and the preparation of longer sequences. Established mitigation strategies include sterically demanding esters and buffered deprotection conditions.^26–28^ These can reduce the amount of aspartimide-derived byproducts but cannot serve as a general solution due to inefficient suppression of aspartimide formation or restrictions to certain amino acids. While we recently reported that cyanosulfurylide protecting groups for aspartic acid completely prevents aspartimide formation during SPPS, their conversion to the free carboxylic acid requires oxidants that are not compatible with methionine or common cysteine protecting groups. Furthermore, their removal requires a separate step outside of the usual workflows of SPPS, hindering its wider application in the field of peptide chemistry.

We applied Fmoc-Asp(CyPY)-OH 12 for the total chemical synthesis of a variety of LA modules of the human low-density lipoprotein receptor (LDLR). These small protein domains are involved in mediating cholesterol homeostasis in mammalian cells, endocytosis of several ligands and viral uptake.^2,6^ However, their synthesis is hindered due the high abundance of aspartic acid residues and the formation of aspartimide during SPPS.^15^ We could overcome these obstacles using Fmoc-Asp(CyPY)-OH 12 and successfully chemically synthesized two monomeric LA modules (R3 and R4) as well as their linear dimeric LA module (R3–R4).

The chemical synthesis of LA modules reported here will complement the existing work on extracellular receptor domains and enable further efforts on cysteine-rich ligand-binding domains of modest size for therapeutic applications.^9^ Notable prior studies include the synthesis of structurally related EGF modules, for example Nishimura's insightful studies on the synthesis of human Notch 1 EGF modules and the effect of glycosylation on ligand binding.^29,30^ Craik designed EGF-based inhibitors for proprotein convertase with high affinity.^31^ The chemical synthesis of folded protein domains has shown value for the design of variants with tailored properties. This work therefore expands the toolbox for such studies by providing a general applicable route to access synthetic LA modules.

## Conclusions

In summary, we have identified a new class of ylide-based carboxylic acid protecting groups that suppresses aspartimide formation during SPPS and which can be removed by a simple adaptation of standard resin cleavage and global deprotection procedures. These cyanopyridiniumylides (CyPY) are easily prepared and are fully compatible with all standard amino acid residues and protecting group strategies, including methionine and Cys(Acm), as well as the building blocks employed for KAHA ligation. With these new Asp-forming monomers, we could readily prepare, isolate, and fold challenging Cys- and Asp-rich LA domains, as well as a ligated heterodimer. The CyPY carboxylic acid protecting group should be broadly applicable to other challenging peptide sequences and further enable the synthesis and study of synthetic peptides and proteins.

## Author contributions

KN and JB conceived the project and planned all experiments. KN, SK, AZ and SB synthesized peptides and analysed peptides. AV carried out small molecule synthesis and stability studies. KN performed folding of domains. The manuscript was written by KN and JB with contributions from all authors.

## Conflicts of interest

There are no conflicts to declare.

## Supplementary Material

CB-004-D2CB00234E-s001
