# Supplementary material for: Synthesis of multi-module low density lipoprotein receptor class A domains with acid labile cyanopyridiniumylides (CyPY) as aspartic acid masking groups
Source: RSC Chem Biol. 2023 Jan 24;4(4):292–9. doi: 10.1039/d2cb00234e (PMC10074552; doi:10.1039/d2cb00234e)
Supplement: CB-004-D2CB00234E-s001 [file CB-004-D2CB00234E-s001.pdf]

Electronic Supplementary Material (ESI) for RSC Chemical Biology.
This journal is © The Royal Society of Chemistry 2023

Supplementary Information

Synthesis of Multi-Module Low Density Lipoprotein Receptor Class A Domains with Acid Labile Cyanopyridiniumylides (CyPY) as Aspartic Acid Masking Groups

Kevin Neumann,[a,b] Alex Vujinovic,[a] Saidu Kamara,[a] André Zwicky,[a] Simon Baldauf,[a] Jeffrey Bode[a]*

[a]Laboratorium für Organische Chemie, Department of Chemistry and Applied Biosciences, ETH Zürich, 8093 Zürich, Switzerland

[b]Systems Chemistry Department, Institute for Molecules and Materials, Radboud University Nijmegen, Heyendaalseweg 135, 6525 AJ Nijmegen, Netherlands.

bode@org.chem.ethz.ch

Table of Contents

1.	General Information	3
2.	Solid Phase Peptide Synthesis	5
3.	Acid Mediated CyPY-removal	6
4.	Stability of CyPY	7
5.	Kinetics of Acid Mediated Hydrolysis	8
6.	Hydrolysis Mediated by Acetic Acid	10
7.	Typical Procedure for CyPY-removal	11
8.	Minimization of Aspartimide During CyPY-removal	12
9.	SPPS of LA Modules Peptide with CyPY	14
10.	Folding of LA modules	16
11.	Experimental	18
12.	NMR-Spectra	37
13.	HRMS Sperctra	49
14.	MS Spectra	54
15.	Crystal Structures	58
16.	References	60

1. General Information

Reagents

Fmoc-amino acids with side-chain protecting groups, HATU (1-[bis(dimethylamino)methylene]-1H-1,2,3-triazolo[4,5-b]pyridinium 3-oxide hexafluorophosphate) and HCTU (*O*-(1*H*-6-chlorobenzotriazol-1-yl)-*N*,*N*,*N*',*N*'-tetramethyluroniumhexafluorophosphate) were purchased from Peptides International (Louisville, KY, USA) and ChemImpex (Wood Dale, IL, USA). Solvents for silica chromatography (MeOH and CH_2Cl_2) were of technical grade. CH_3CN (HPLC grade) from Sigma-Aldrich was used for analytical and preparative HPLC purification. DMF (> 99.8%) from Thommen-Furler AG was directly used without further purification for solid phase peptide synthesis. Other commercially available reagents and solvents were purchased from Sigma-Aldrich (Buchs, Switzerland), TCI Europe (Zwijndrecht, Belgium) and Acros Organics (Geel, Belgium).

Characterization

^{1}H and ^{13}C NMR spectra were recorded on a Bruker AVIII500 spectrometer. Chemical shifts for ^{1}H NMR (500 MHz) and ^{13}C NMR (126 MHz) are expressed in parts per million and are referenced to residual un-deuterated solvent signals. Coupling constants are reported in Hertz (Hz) and the corresponding splitting patterns are indicated as follows: s, singlet; bs, broad singlet; d, doublet; dd, doublet of doublet; ddd, doublet of doublet of doublet; td, triplet of doublet; t, triplet; m, multiplet. High-resolution mass spectra were recorded by the Molecular and Biomolecular Analysis Service (MoBiAS) at ETH Zurich, either with a Bruker maXis instrument (ESI-MS measurements) equipped with an ESI source and a Qq-TOF detector or with a Bruker solariX instrument (MALDI-FTICR-MS) using 4-hydroxy-α-cyanocinnamic acid as the matrix.

Reactions and purification

All reactions were performed using standard practical laboratory techniques under an atmosphere of N_2. Reactions and fractions from flash column chromatography were monitored by thin layer chromatography using aluminum TLC plates (Merck, TLC Silica gel 60 W F_{254}s) and, if necessary, visualized by staining with basic $KMnO_4$ solution or acidic ninhydrin solution. Flash column chromatography was performed on Sigma-Aldrich SiO_2 Type F60 (230-400 mesh) using a forced flow of air at 0.5-1.0 bar. Unless otherwise stated, peptides and protein segments were analyzed and purified by reversed phase high performance liquid chromatography (RP-HPLC) on Jasco analytical and preparative instruments equipped with dual pumps, mixer and in-line degasser, a variable wavelength UV detector (simultaneous monitoring of the eluent at 220 nm, 254 nm and 301 nm) and a Rheodyne injector fitted with a 20 or 1000 µL injection loop. If required, the columns were heated using an Alltech column heater or a H_2O bath (preparative HPLC). The mobile phase for RP-HPLC were Milipore-H_2O containing 0.1% (v/v) TFA and HPLC grade CH_3CN containing 0.1% (v/v) TFA. Analytical HPLC was performed on Shiseido Capcell Pak C18 MGII (5 µm, 4.6 mm I.D. x 250 mm) or Shiseido Capcell Pak C18 (UG 80, 5 µm, 4.6 mm I.D. x 250 mm) columns at a flow rate of 1 ml/min. Preparative HPLC was performed on Shiseido Capcell Pak MGII (5 µm, 20 mm I.D. x 250 mm) at a flow rate of 10 mL/min.

Peptide amounts below 10 mg were determined in solution by UV-absorption (λ = 280 nm) with extinction coefficient of given peptide.

General *analytical* HPLC methods:

- flow 1 mL/min, isocratic 10% CH_3CN for 3 min, then gradient from 10% to 90% CH_3CN in 22 min

General *analytical* LC-MS methods:

- flow 0.5 mL/min, isocratic 2% CH_3CN for 0.5 min, then gradient from 2% to 98% CH_3CN in 6 min

List of general *preparative* HPLC methods:

- Method A: flow 10 mL/min, isocratic 10% CH_3CN for 5 min, then gradient from 20% to 70% CH_3CN in 28 min.
- Method B: flow 40 mL/min, isocratic 20% CH_3CN for 10 min, then gradient 20% to 70% CH_3CN in 40min.

2. Solid phase peptide synthesis

Loading of amino acids on solid support was performed as followed:

- chloro-trityl resin: The amino acid (1.20 equiv of desired loading) was dissolved in CH_2Cl_2 (500mM). NEt^iPr_2 (2 equiv) was added to the solution. The solution was added to preswollen chloro-trityl resin and was shaken for 1 h. The resin was washed three times with CH_2Cl_2. Remaining chloro-trityl moieties were capped with CH_2Cl_2/MeOH/DIPEA (17:2:1, v:v:v) for 1 min. The resin was washed with CH_2Cl_2 and used immediately for SPPS.
- Rink-amide resin: Rink amide resin was first Fmoc deprotected using 20% Piperidine in DMF (2 x 10 min). The resin was washed six times with DMF. The amino acid (1.20 equiv of target loading) and HCTU (1.15 equiv of target loading) was dissolved in DMF (400 mM). NMM (2.50 equiv) was added to the solution. The solution was added to preswollen Rink-amide resin and shaken for 6 h. The resin was washed three times with DMF and CH_2Cl_2. Remaining active amines were capped with DMF/DIPEA/acetic acid anhydride (10:2:1, v:v:v). The resin was washed with CH_2Cl_2 and stored at 5°C.

Peptides were synthesized on a Multisyntech Syro I parallel synthesizer using Fmoc-SPPS chemistry. The following Fmoc amino acids with side-chain protection groups were used: Fmoc-Ala-OH, Fmoc-Arg(Pbf)-OH, Fmoc-Asn(Trt)-OH, Fmoc-Asp(OtBu)-OH, Fmoc-Cys(S^tBu)-OH, Fmoc-Gln(Trt)-OH, Fmoc-Glu(OtBu)-OH, Fmoc-Gly-OH, Fmoc-His(1-Trt)-OH, Fmoc-Ile-OH, Fmoc-Leu-OH, Fmoc-Lys(Boc)-OH, Fmoc-Met-OH, Fmoc-Nle-OH, Fmoc-Phe-OH, Fmoc-Pro-OH, Fmoc-Ser(tBu)-OH, Fmoc-Thr(tBu)-OH, Fmoc-Trp(Boc)-OH, Fmoc-Tyr(tBu)-OH, Fmoc-Val-OH.

3. Acid Mediated CyPY-removal

CyPY **1** (10 mg, 0.04 mmol) was dissolved in D_2O/d_6-DMSO (4/1, v/v, 0.7 mL). Aqueous HCl (4 vol%) was added and the solution was stirred at room temperature. After one hour the reaction mixture was analyzed by ^{1}H-NMR and LC-MS confirming the formation of carboxylic acid **6** and pyridinium salt **7**.

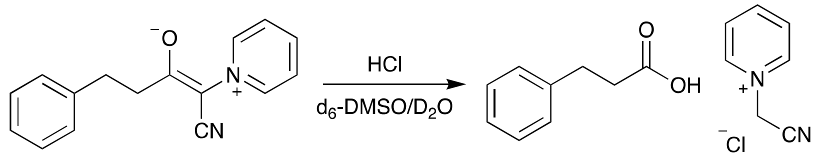

1 **6** **7**

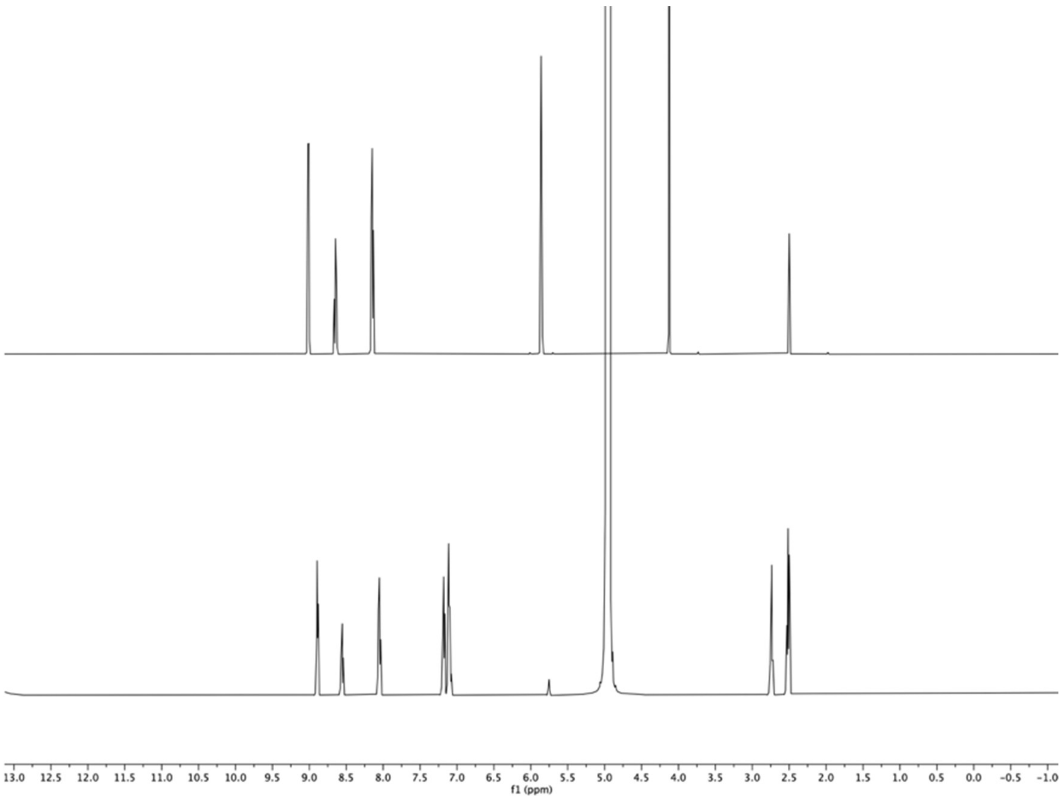

Figure S1 Top: ^{1}H-NMR of pyridinium salt **7** as reference. Button: ^{1}H-NMR of reaction mixture after one hour.

4. Stability of Cyanopyridiniumylides

Cyanopyridiniumylides were tested for their stability in the presence of electrophiles (acetic acid anhydride), aqueous basic and neutral conditions. Typically, cyanopyridiniumylide (2 mg) was dissolved in H_2O/CH_3CN (1/1, v/v, 1 ml) or CH_3CN (1 ml) and incubated at 45°C. The exact conditions are listed in Table S1. The stability was determined by LC-MS.

Table S1 CyPY **1-4** was incubated under different conditions (entry 1 – 7) in order to determine the stability by LC-MS.

Entry	CyPY	Condition	Result
1	**1** (-H)	Ac_2O (10 v% in CH_3CN) for 3 h	Stable
2	**2** (-OMe)	Ac_2O (10 v% in CH_3CN) for 3 h	Stable
3	**3** (-amide)	Ac_2O (10 v% in CH_3CN) for 3 h	Stable
4	**4** (-Cl)	Ac_2O (10 v% in CH_3CN) for 3 h	Stable
5	**1** (-H)	H_2O/CH_3CN for 36 h	Stable
6	**1** (-H)	0.5M KOH in H_2O/CH_3OH for 12 h	Stable
7	**2** (-OMe)	0.5M KOH in H_2O/CH_3OH for 12 h	Methoxy is converted to free phenol

5. Kinetics of Acid Mediated Hydrolysis

To determine rates of hydrolysis under acidic conditions, cyanopyridiniumylides **1** – **4** were incubated in D_2O/CD_3CN (1/1, v/v, 25 to 35 mM) at 45 °C with 2 vol% aq. HCl. Samples were taken at different time points and diluted with aqueous $NaHCO_3$ in order to stop the reaction. Time points were taken in triplicate. The samples were analyzed by ^{1}H-NMR. To determine conversion the methylene groups of starting material and product were compared. First order rate constants k_1 were calculated assuming a pseudo first order kinetic.

$$\ln(c_t) = -kt + \ln(c_o)$$

with the half-life $t_{1/2}$

$$t_{1/2} = \frac{ln2}{k}$$

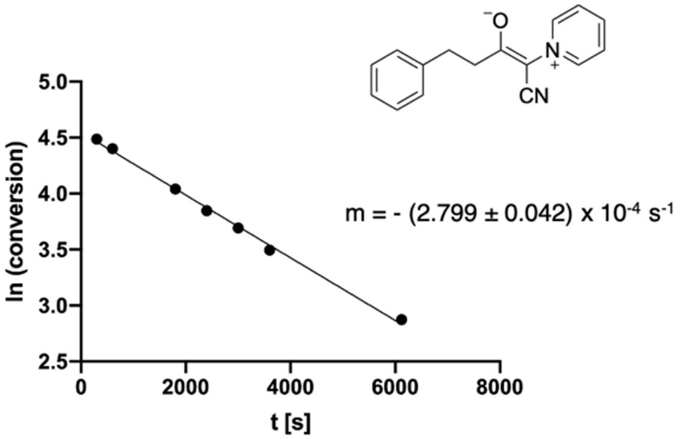

Figure S2 CyPY 1 displays a half-life of $t_{1/2}$ = 41.3 ± 0.6 min.

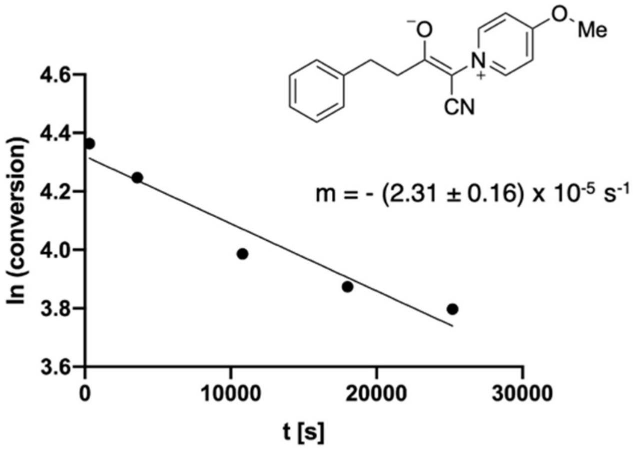

Figure S3 CyPY 2 displays a half-life of $t_{1/2}$ = 500.1 ± 34.6 min.

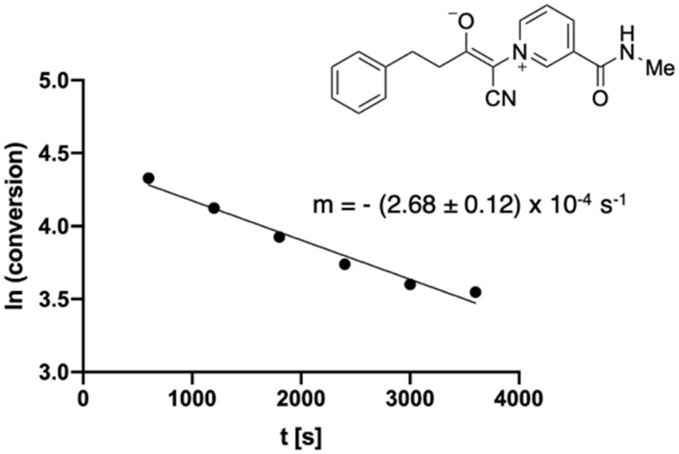

Figure S4 CyPY 3 displays a half-life of $t_{1/2}$ = 43.1 ± 1.9 min.

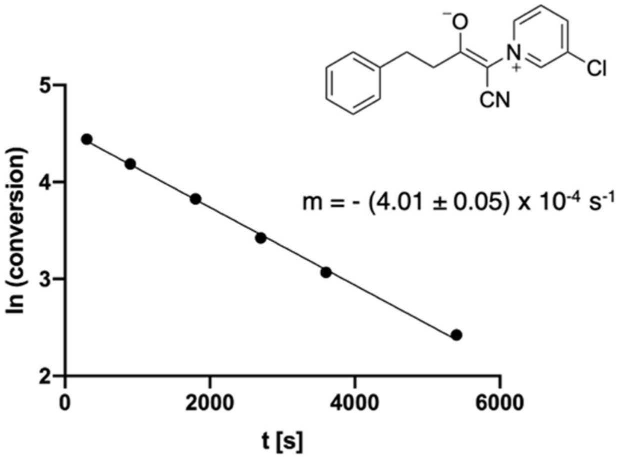

Figure S5 CyPY 4 displays a half-life of $t_{1/2}$ = 28.8 ± 0.4 min.

6. Hydrolysis Mediated by Acetic Acid

The stability of cyanopyridiniumylide **1** was tested in the presence of acetic acid and water. Cyanopyridiniumylide **1** (3 mg) was dissolved in H_2O/AcOH (4/1, v/v, 1 ml) and incubated at 45°C. The reaction was monitored with LC-MS. It was observed that also acetic acid mediates hydrolysis to the free carboxylic acid. The hydrolysis, however, requires much longer reaction time compared to hydrolysis mediated by aqueous HCl.

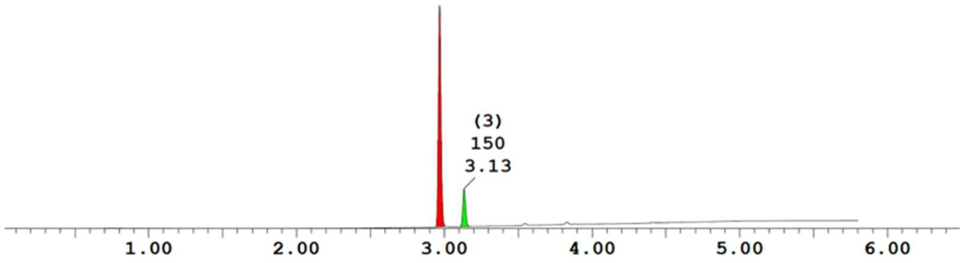

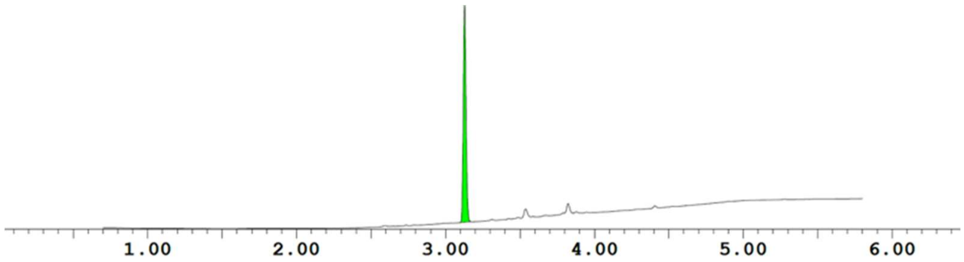

Figure S6 LC-MS traces of acetic acid mediated hydrolysis of **CyPY 1** after t = 2 h (top) and t = 12 h (below). After 12 h CyPY is fully converted to the free carboxylic acid.

7. Typical Procedure for CyPY-removal

Global deprotection including resin cleavage was carried out using standard protocol (thiol- or silane-based scavengers). After two hours, the resin was separated from the cleavage cocktail by filtration and added into Et_2O. The formed precipitate was filtered off and was washed once more with diethyl ether. For CyPY-removal, the crude peptide was dissolved in acidified water (1 v% to 5 v% HCl). For clean conversion from CyPY to carboxylic acid, as little organic co-solvent as possible should be used (DMSO is preferred over acetonitrile, page 12). The reaction time is strongly sequence-depending and varied. For some peptide sequences, it was necessary to carry out the deprotection at increased temperature (e.g. 35 °C or higher) to guarantee full CyPY-removal. However, higher temperature increases the possibility of potential side-reactions such as aspartimide formation (see page 12). Once full CyPY-removal was indicated by HPLC or mass spectroscopy, the reaction mixture was purified by reverse phase HPLC.

8. Minimization of Aspartimide During CyPY-removal

Since we observed some formation of aspartimide during CyPY removal, we investigated the influence of solvent systems, temperature and concentration of acid. We synthesized a Fmoc-containing model-peptide **S1** that required the use of an organic co-solvent for full solubility. The peptide was treated with acid in either DMSO/water or acetonitrile/water mixtures at different temperatures. We observed that an increasing amount of acetonitrile results in enhanced formation of aspartimide as side product. Less aspartimide was formed using DMSO/water mixtures. In addition, we observed that less aspartimide was formed as a side-product if lower temperature was used. The amount of acid seemed to have only minor influence on the formation of undesired side-product such as aspartimide. Selected HPLC traces are shown in Figure S9.

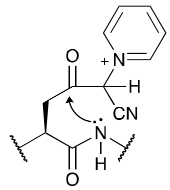

Figure S7 The formation of aspartimide is a competing side-reaction during CyPY-removal.

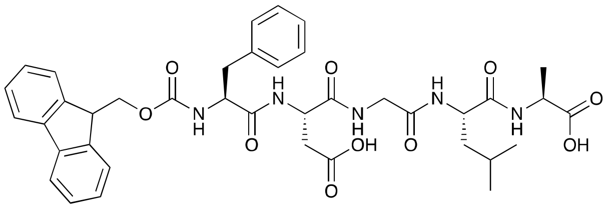

Figure S8 Fmoc-terminated model-peptide **S1** that requires an organic-solvent during CyPY-removal.

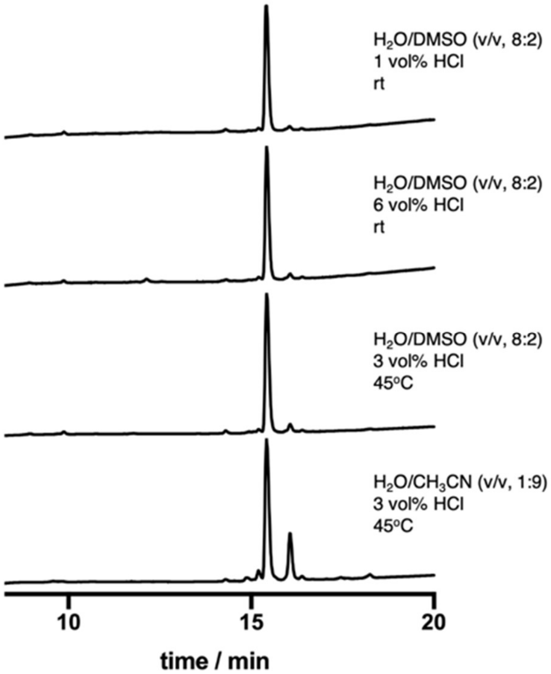

Figure S9 HPLC traces obtained after CyPY-removal from model-peptide **S1**. The results show that aspartimide formation as side-reaction during CyPY removal can be suppressed using higher proportions of water and DMSO as an organic co-solvent.

9. SPPS of LA Modules Peptide with CyPY

Below crude and purified HPLC traces are shown as well as mass spectra of purified samples. The exact experimental protocol is described in Experimental section.

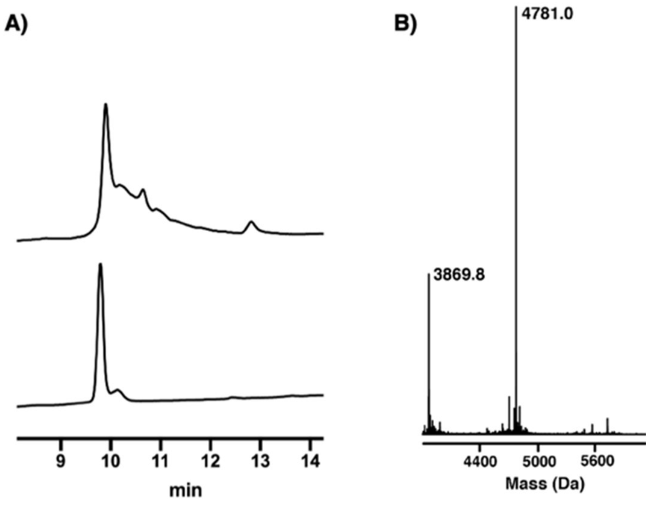

Figure S10 LA(Acm)$_6$-3 module 20 A) Top: crude HPLC trace after SPPS; Button: HPLC trace of purified peptide **B)** The recorded m/z data was deconvoluted by applying MaxEnt3 algorithm and mass of first isotope was calculated (for full spectrum refer to section 13).

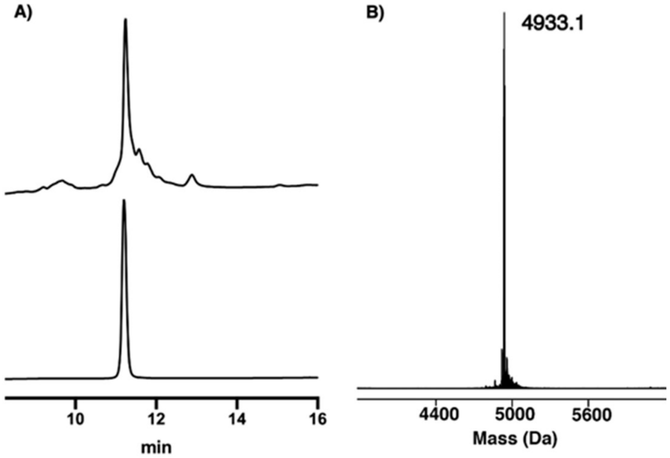

Figure S11 LA(Acm)$_6$-4 module 21 A) Top: crude HPLC trace after SPPS; Button: HPLC trace of purified peptide **B)** The recorded m/z data was deconvoluted by applying MaxEn1 algorithm and average mass was calculated (for full spectrum refer to section 13).

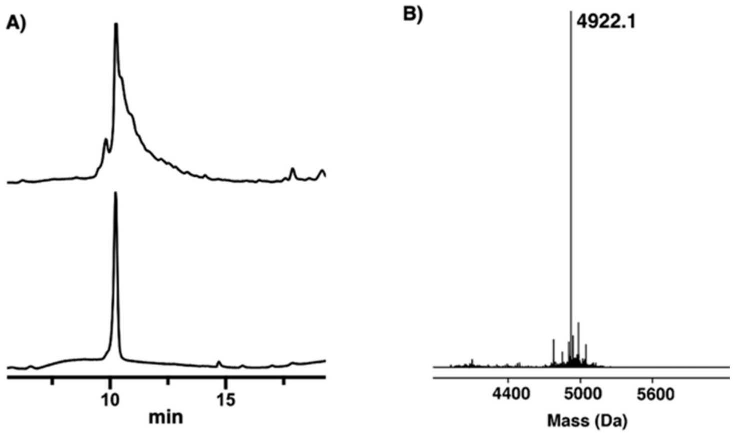

Figure S12 Synthesis of LA(SAcm)$_6$-3 ketoacid module 25 A) Top: crude HPLC trace after SPPS; Button: HPLC trace of purified peptide **B)** The recorded m/z data was deconvoluted by applying MaxEnt3 algorithm and mass of first isotope was calculated (for full spectrum refer to section 13).

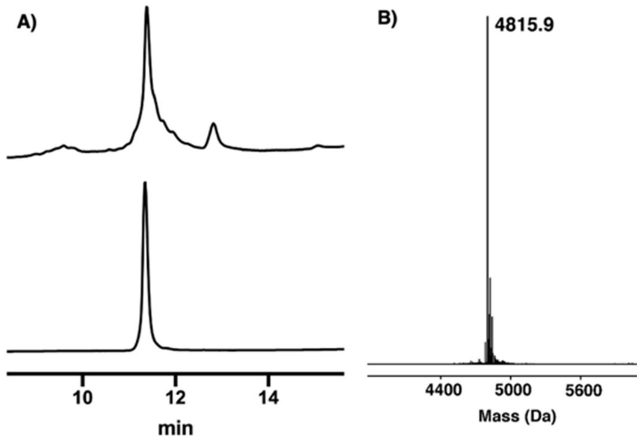

Figure S13 LA(SAcm)$_6$-4 hydroxylamine module 26 A) Top: crude HPLC trace after SPPS; Button: HPLC trace of purified peptide **B)** The recorded m/z data was deconvoluted by applying MaxEnt3 algorithm and mass of first isotope was calculated (for full spectrum refer to section 13).

10. Folding of LA modules

Folding was carried out according to a literature report.[1] Briefly, the unfolded LA modules were dissolved (150 µg mL^{-1}; 100 µg mL^{-1} for heterodimer) in folding buffer at pH 8.5 containing of GSH (3 mM), GSSG (0.3 mM), NaCl (150 mM), Tris-HCl (50 mM) and $CaCl_2$ (2.5 mM) at 4 °C. For optimal conversion during folding it is crucial to pre-cool the folding buffer.

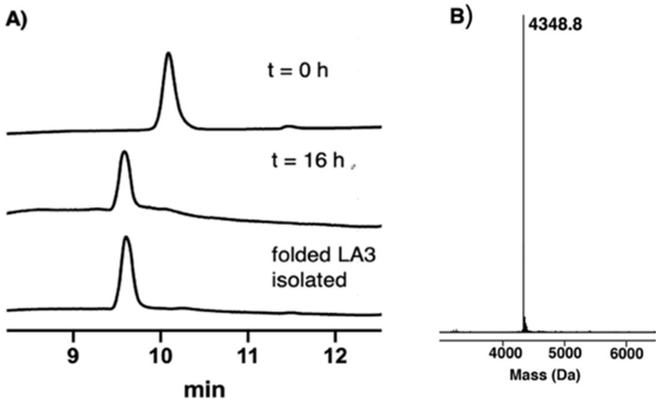

Figure S14 Crude HPLC trace of LA module 3 after 16h in folding buffer and preparative HPLC purified LA module 3. The recorded m/z data was deconvoluted by applying the MaxEnt3 algorithm and mass of first isotope was calculated (for full spectrum refer to section 13). For HRMS see page S53.

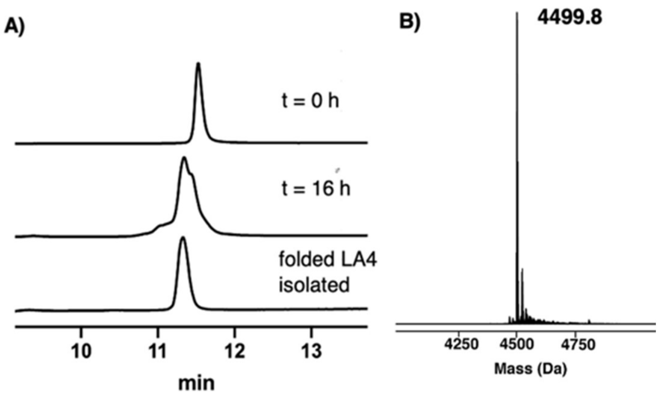

Figure S15 Crude HPLC trace of LA module 4 after 16h in folding buffer and preparative HPLC purified LA module 4. The recorded m/z data was deconvoluted by applying the MaxEnt1 algorithm and monoisotopic mass was calculated (for full spectrum refer to section 13). For HRMS see page S53.

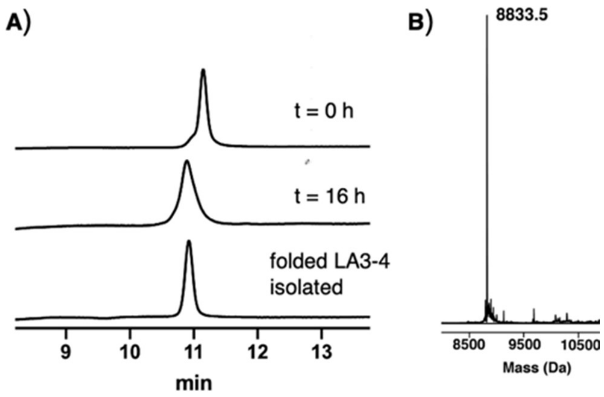

Figure S16 Crude HPLC trace of dimeric LA module 3-4 after 16h in folding buffer and preparative HPLC purified dimeric LA module 3-4. The recorded m/z data was deconvoluted by applying the MaxEn1 algorithm and average mass was calculated (for full spectrum refer to section 13).

11. Experimental

General procedure A for the synthesis of hydrocinnamic acid cyanopyridiniumylides **1 – 5**

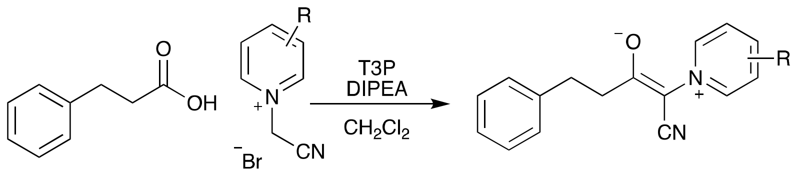

Hydrocinnamic acid (1.00 equiv) was dissolved in CH_2Cl_2 (300 mM). *N,N*-diisopropylethylamine (3.00 equiv) was added dropwise followed by dropwise addition of propylphosphonic anhydride (T3P) (≥ 50 %w/w in EtOAc, 1.20 equiv). The solution was stirred for 5 min before N-acetonitrilepyridinium bromide (1.20 equiv) was added in one portion as a solid. The resulting solution was stirred for 12 h at room temperature and then diluted with CH_2Cl_2. The organic layer was washed with sat. aq. $NaHCO_3$ (3x), water (1x) and brine (1x), and dried over Na_2SO_4. The drying agent was removed by filtration and solvent was removed under reduced pressure. The residue was purified by flash column chromatography (CH_2Cl_2:MeOH 60:1 to 30:1) to provide hydrocinnamic acid cyanopyridiniumylides as a yellow solid.

Hydrocinnamic acid cyanopyridiniumylide 1

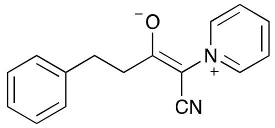

Hydrocinnamic acid cyanopyridiniumylide was synthesized according to general procedure **A** using *N*-acetonitrilepyridinium bromide (0.50 g, 2.56 mmol, 1.2 equiv) and hydrocinnamic acid (0.32 g, 2.11 mmol, 1.0 equiv). The product was obtained as a yellow solid (0.44 g, 1.75 mmol, 82%).

^{1}H NMR (500 MHz, CD_3OD) δ 8.97–8.87 (m, 2H), 8.27–8.20 (m, 1H), 7.91 (m, 2H), 7.31–7.23 (m, 4H), 7.22–7.13 (m, 1H), 3.02 – 2.95 (m, 2H), 2.80–2.73 (m, 2H).

^{13}C NMR (126 MHz, CD_3OD) δ 183.73 (CO), 144.73 (2xCH), 142.68 (CH) 142.49 (C), 129.46 (2xCH), 129.43 (2xCH), 128.41 (2xCH), 127.11 (CH), 122.36 (CN), 91.34 (C), 40.89 (CH_2), 33.70 (CH_2).

HRMS (ESI): calculated for $[C_{16}H_{15}N_2O]^+$: m/z 251.1179 , found : m/z 251.1178.

IR (cm^{-1}, neat): 3066, 3029, 2917, 2156, 1553, 1470.

Hydrocinnamic acid cyano(4-methoxy)pyridiniumylide 2

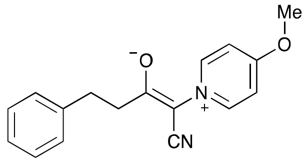

Hydrocinnamic acid cyano(4-methoxy)pyridiniumylide was synthesized according to general procedure **A** using *N*-acetonitrile(4-methoxy)pyridinium bromide (0.55 g, 2.44 mmol, 1.2 equiv) and hydrocinnamic acid (0.31 g, 2.04 mmol, 1.0 equiv). The product was obtained as a yellow solid (0.32 g, 1.14 mmol, 56%).

^{1}H NMR (500 MHz, CD_3OD) δ 8.47 (d, J = 7.62 Hz, 2H), 7.44 (d, J = 7.64, 2H), 7.28–7.25 (m, 4H), 7.19–7.17 (m, 1H), 4.13 (s, 3H), 2.98–2.95 (m, 2H), 2.71–2.67 (m, 2H).

^{13}C NMR (126 MHz, CD_3OD) δ 182.65 (CO), 170.28 (C), 147.75 (2xCH), 141.14 (C), 128.12 (2xCH), 128.01 (2xCH), 125.71 (CH), 121.30 (CN), 112.75 (2xCH), 88.88 (C), 57.02 (CH_3), 38.65 (CH_2), 32.50 (CH_2).

HRMS (ESI): calculated for $[C_{17}H_{17}N_2O_2]^+$: m/z 281.1285 , found : m/z 281.1281.

IR (cm^{-1}, neat): 3010, 2166, 1634, 1545, 1516, 1316.

Hydrocinnamic acid cyano(3-(methyl-amide))pyridiniumylide 3

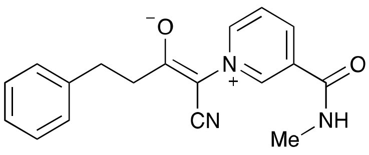

Hydrocinnamic acid cyano(3-(methyl-amide))pyridiniumylide was synthesized according to general procedure **A** using *N*-acetonitrile(3-(methyl-amide))pyridinium bromide (0.50 g, 2.00 mmol, 1.2 equiv) and hydrocinnamic acid (0.25 g, 1.67 mmol, 1.0 equiv). The product was obtained as a yellow solid (0.28 g, 0.83 mmol, 50%).

^{1}H NMR (500 MHz, $CDCl_3$) δ 10.00 (td, J = 1.5, 0.6 Hz, 1H), 9.19 (dt, J = 6.4, 1.3 Hz, 1H), 8.35 (ddd, J = 8.0, 1.7, 1.2 Hz, 1H), 7.75 (dd, J = 1.6, 0.6 Hz, 1H), 7.31–7.24 (m, 4H), 7.21 (m, 1H), 3.04–2.99 (m, 5H), 2.94–2.88 (m, 2H).

^{13}C NMR (126 MHz, $CDCl_3$) δ 183.55 (CO), 162.34 (CONH), 141.22 (C), 139.37 (CH), 137.10 (CH), 136.68 (CH), 134.07 (C), 128.57 (2xCH), 128.51 (2xCH), 126.49 (CH), 126.21 (CH), 121.22 (CN), 90.08 (C), 40.64 (CH_2), 32.11 (CH_2), 27.28 (CH_3).

HRMS (ESI): calculated for $[C_{18}H_{17}N_3O_2Na]^+$: m/z 330.1213 , found : 330.1212.

IR (cm^{-1}, neat): 3261, 3073, 2178, 1666, 1531, 1373.

Hydrocinnamic acid cyano(3-chloro)pyridiniumylide 4

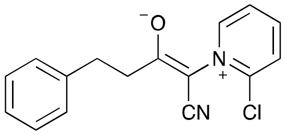

Hydrocinnamic acid cyano(3-chloro)pyridiniumylide was synthesized according to general procedure **A** using *N*-acetonitrile(3-chloro)pyridinium bromide (0.60 g, 2.61 mmol, 1.2 equiv) and hydrocinnamic acid (0.33 g, 2.18 mmol, 1.0 equiv). The product was obtained as a yellow solid (0.41 g, 1.44 mmol, 66%).

^{1}H NMR (500 MHz, d_6-DMSO) δ 9.62 (td, *J* = 1.5, 0.7 Hz, 1H), 9.01 (ddd, *J* = 6.4, 1.4, 0.9 Hz, 1H), 8.22 (ddd, *J* = 8.4, 2.0, 0.9 Hz, 1H), 7.90 (ddd, *J* = 8.4, 6.4, 0.5 Hz, 1H), 7.32 – 7.20 (m, 4H), 7.17 (d, *J* = 7.1 Hz, 1H), 2.90 (dd, *J* = 9.2, 6.5 Hz, 2H), 2.76 – 2.69 (m, 2H).

^{13}C NMR (126 MHz, d_6-DMSO) δ 181.10 (CO), 141.50 (C), 137.81 (CH), 137.68 (CH), 137.51 (CH), 132.82 (C), 128.28 (2xCH), 128.19 (2xCH), 127.66 (CH), 125.80 (CH), 121.20 (CN), 88.47 (C), 39.93 (CH_2), 31.23 (CH_2).

HRMS (ESI): calculated for $[C_{16}H_{14}ClN_2O]^+$: m/z 285.0789 , found : m/z 285.0788.

IR (cm^{-1}, neat): 3109, 3062, 3034, 2935, 2172, 1572, 1448.

Hydrocinnamic acid cyano(4-phenyl)pyridiniumylide 5

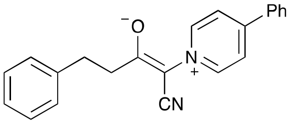

Hydrocinnamic acid cyano(4-phenyl)pyridiniumylide was synthesized according to general procedure **A** using *N*-acetonitrile(4-phenyl)pyridinium bromide (0.30 g, 1.11 mmol, 1.2 equiv) and hydrocinnamic acid (0.14 g, 0.93 mmol, 1.0 equiv). The product was obtained as a yellow solid (0.09 g, 0.28 mmol, 30%).

^{1}H NMR (500 MHz, $CDCl_3$) δ 9.34–9.30 (m, 2H), 7.88–7.84 (m, 2H), 7.72 (ddd, J = 5.8, 2.9, 1.4 Hz, 2H), 7.61–7.56 (m, 3H), 7.33–7.29 (m, 4H), 7.22–7.19 (m, 1H), 3.10–3.01 (m, 2H), 2.96–2.91 (m, 2H).

^{13}C NMR (126 MHz, $CDCl_3$) δ 183.23 (CO), 149.10 (C), 141.67 (C), 139.00 (2xCH), 134.61 (C), 131.40 (CH), 129.92 (2xCH), 128.65 (2xCH), 128.46 (2xCH), 127.18 (2xCH), 126.01 (CH), 123.46 (2xCH), 122.04 (CN), 89.09 (C), 40.79 (CH_2), 32.34 (CH_2).

HRMS (ESI): calculated for $[C_{22}H_{19}N_2O]^+$: m/z 327.1492 , found : m/z 327.1490.

IR (cm^{-1}, neat): 3027, 2167, 1564, 1465, 1321.

General procedure B for the synthesis of N-acetonitrilepyridiniums **7 – 11**

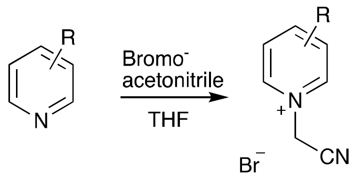

Pyridine (1.1 equiv) was dissolved in THF (1.5M) under a nitrogen atmosphere. Bromoacetonitrile (1.0 equiv) was added to the solution. The solution was stirred for 24 h at room temperature. The resulting solid was filtered and washed with Et_2O. The white solid was dried under reduced pressure at room temperature.

N-Acetonitrile-pyridinium Bromide 7

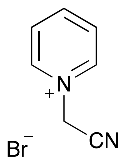

N-Acetonitrile-pyridinium bromide was synthesized according to the general procedure **B** using pyridine (1.0 mL, 12.3 mmol, 1.1 equiv) and bromoacetonitrile (0.8 mL, 11.5 mmol, 1.0 equiv). The product was obtained as a white solid (1.4 g, 7.8 mmol, 68%).

^{1}H NMR (500 MHz, d_6-DMSO) δ 9.32 – 9.21 (m, 2H), 8.75 (d, J = 7.9 Hz, 1H), 8.35 – 8.23 (m, 2H), 6.10 (s, 2H).

^{13}C NMR (126 MHz, d_6-DMSO) δ 147.57, 145.44, 128.54, 114.30, 47.63.

HRMS (ESI): calculated for $[C_7H_7N_2]^+$: m/z 119.0602 , found : m/z 119.0602.

IR (cm^{-1}, neat): 3035, 2922, 2862, 2343, 1626.

N-Acetonitrile(4-methoxy)pyridinium Bromide 8

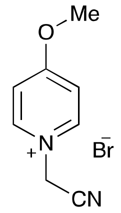

N-Acetonitrile(4-methoxy)pyridinium bromide was synthesized according to general procedure **B** using (4-methoxy)pyridine (1.0 mL, 9.8 mmol, 1.1 equiv) and bromoacetonitrile (0.6 mL, 9.0 mmol, 1.0 equiv). The product was obtained as a white solid (1.4 g, 6.3 mmol, 70%).

^{1}H NMR (500 MHz, d_6-DMSO) δ 9.07 (d, J = 7.7 Hz, 2H), 7.78 (d, J = 7.6 Hz, 2H), 5.95 (s, 2H), 4.14 (s, 3H).

^{13}C NMR (126 MHz, d_6-DMSO) δ 171.80, 146.60, 114.72, 113.95, 58.59, 45.68.

HRMS (ESI): calculated for $[C_8H_9N_2O]^+$: m/z 149.0709 , found : m/z 149.0707.

IR (cm^{-1}, neat): 2939, 1640, 1521, 1312, 1186.

N-Acetonitrile-[3-(methyl-amide)]pyridinium Bromide 9

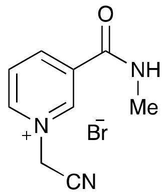

N-Acetonitrile-[3-(methyl-amide)]pyridinium bromide was synthesized according to general procedure **B** using (3-methyl amide)pyridine (1 g, 7.4 mmol, 1.1 equiv) and bromoacetonitrile (0.5 mL, 6.7 mmol, 1.0 equiv). The product was obtained as a white solid (1.0 g, 4.0 mmol, 60%).

^{1}H NMR (500 MHz, d_6-DMSO) δ 9.64 (m, 1H), 9.37 (m, 1H), 9.29 (d, J = 4.7 Hz, 1H), 9.13 (ddd, J = 8.2, 1.8, 1.2 Hz, 1H), 8.39 (ddd, J = 8.2, 6.2, 0.6 Hz, 1H), 6.13 (s, 2H), 2.85 (d, J = 4.6 Hz, 3H).

^{13}C NMR (126 MHz, d_6-DMSO) δ 161.70, 147.28, 146.06, 145.29, 134.35, 128.74, 114.55, 48.34, 26.86.

HRMS (ESI): calculated for $[C_9H_{10}N_3O]^+$: m/z 176.0818 , found : m/z 176.0817.

IR (cm^{-1}, neat): 3208, 3043, 3002, 2934, 1672, 1546, 1497.

N-Acetonitrile-(3-chloro)pyridinium Bromide 10

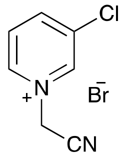

N-Acetonitrile-(3-chloro)pyridinium bromide was synthesized according to general **B** procedure using (3-chloro)pyridine (1 mL, 10.6 mmol, 1.1 equiv) and bromoacetonitrile (0.7 mL, 9.7 mmol, 1.0 equiv). The product was obtained as a white solid (1.4 g, 6.3 mmol, 65%).

^{1}H NMR (500 MHz, d_6-DMSO) δ 9.65 (m, 1H), 9.26 (m, 1H), 8.92 (ddd, J = 8.5, 2.1, 1.1 Hz, 1H), 8.31 (ddd, J = 8.5, 6.1, 0.5 Hz, 1H), 6.04 (s, 2H).

^{13}C NMR (126 MHz, d_6-DMSO) δ 147.13, 145.15, 144.32, 134.18, 129.15, 113.84, 47.68.

HRMS (ESI): calculated for $[C_7H_6ClN_2]^+$: m/z 153.0214 , found : m/z 153.0712.

IR (cm^{-1}, neat): 2993, 2936, 2897, 1627, 1492, 1468.

N-Acetonitrile-(4-phenyl)pyridinium Bromide 11

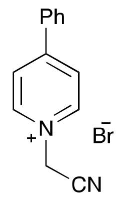

N-Acetonitrile-(4-phenyl)pyridinium bromide was synthesized according to general procedure **B** using (4-phenyl)pyridine (1 g, 6.4 mmol, 1.1 equiv) and bromoacetonitrile (0.4 mL, 5.9 mmol, 1.0 equiv). The product was obtained as a white solid (0.7 g, 2.7 mmol, 45%).

^{1}H NMR (500 MHz, d_6-DMSO) δ 9.24 (d, J = 7.1 Hz, 2H), 8.65 (d, J = 7.1 Hz, 2H), 8.12 (dd, J = 8.1, 1.5 Hz, 2H), 7.74 – 7.62 (m, 3H), 6.01 (s, 2H).

^{13}C NMR (126 MHz, d_6-DMSO) δ 156.41, 145.38, 133.19, 132.66, 129.78, 128.41, 124.84, 114.44, 46.82.

HRMS (ESI): calculated for $[C_{13}H_{11}N_2]^+$: m/z 195.0917 , found : m/z 195.0914.

IR (cm^{-1}, neat): 3017, 2878, 1635, 1596, 1553, 1521.

Fmoc-Asp(CyPY)-O^{t}Bu 13

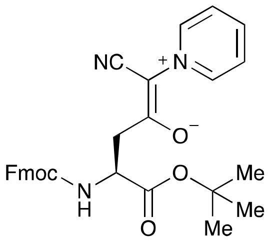

Fmoc-Asp(OH)OtBu **14** (5.00 g, 12.1 mmol, 1.00 equiv) was dissolved in CH_2Cl_2 (50 mL). *N*,*N*-Diisopropylethylamine (6.00 mL, 36.3 mmol, 3.00 equiv) was added dropwise followed by dropwise addition of propylphosphonic anhydride (T3P) (≥ 50%w/w in EtOAc; 9.23 mL, 14.5 mmol, 1.20 equiv). The solution was stirred for 5 min before pyridinium **7** (2.90 g, 14.5 mmol, 1.20 equiv) was added in one portion as a solid. The resulting solution was stirred for 12 h at room temperature and diluted with CH_2Cl_2 (250 ml). The organic layer was washed with sat. aq. $NaHCO_3$ (3x), water (1x) and brine (1x), and dried over Na_2SO_4. The drying agent was removed by filtration and solvent was removed under reduced pressure. The residue was purified by flash column chromatography (CH_2Cl_2:MeOH 60:1 to 30:1) to give Fmoc-Asp(CyPY)OtBu **13** (4.63 g, 9.08 mmol, 75%) as a yellow white foam.

^{1}H NMR (500 MHz, d^6-DMSO) δ 9.14–9.09 (m, 2H), 8.21 (tt, J = 7.7, 1.3 Hz, 1H), 7.99–7.93 (m, 2H), 7.92–7.87 (m, 2H), 7.72 (ddt, J = 7.6, 2.1, 1.0 Hz, 2H), 7.63 (d, J = 8.3 Hz, 1H), 7.42 (tt, J = 6.7, 1.1 Hz, 2H), 7.32 (td, J = 7.4, 1.1 Hz, 2H), 4.48 (ddd, J = 8.6, 6.8, 5.9 Hz, 1H), 4.33–4.22 (m, 3H), 2.82 (qd, J = 15.1, 6.4 Hz, 2H), 1.39 (s, 9H).

^{13}C NMR (126 MHz, d^6-DMSO) δ 177.92 (CO), 171.46 (CO), 156.31 (CO), 144.30 (2XC), 141.76 (2xCH), 141.18 (2xC), 140.57 (CH), 128.10 (2xCH), 127.71 (2xCH), 127.52 (2xCH), 125.73 (2xCH), 122.21 (CN), 120.58 (2xCH), 88.99 (C), 80.96 (C), 66.14 (CH_2), 51.81 (CH), 47.09 (CH), 39.97 (CH_2), 28.08 (CH_3).

HRMS (ESI): calculated for $[C_{30}H_{30}N_3O_5]^+$: m/z 512.2180 , found : 512.2175.

IR (cm^{-1}, neat): 3324, 3064, 2977, 2168, 1714, 1569, 1472.

$[\alpha]_{546}^{23.21}$ (c = 0.08, CH_3OH) = – 20.10

Fmoc-Asp(CyPY)-OH 12

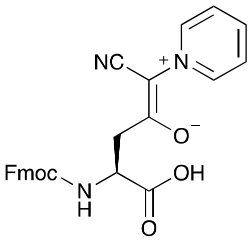

Fmoc-Asp(CyPY)OtBu **13** (2.0 g, 3.9 mmol, 1 equiv) was dissolved in anhydrous CH_2Cl_2 (6.5 ml, 600 mM) followed by the addition of triisopropylsilane (0.8 ml, 3.9 mmol, 1 equiv). Anhydrous trifluoroacetic acid (6.5 ml) was added to the solution and the mixture was stirred vigorously. The reaction was monitored by LC-MS. After full conversion was indicated (1 to 2 h) the solution was added to Et_2O (12 times the volume of TFA/CH_2Cl_2). The resulting solid was separated by centrifugation or filtration and dissolved in anhydrous THF. The solution was washed with aq. sodium bicarbonate/sodium sulfate solution (0.25 M $NaHCO_3$, 1.0 M Na_2SO_4) and aq. sodium sulfate solution (1.0 M Na_2SO_4). The organic layer was concentrated under reduced pressure to half of the volume. The solution was added into phosphate buffer (pH = 4, 10 mM sodium phosphate buffered, 100 mM NaCl) and extracted with CH_2Cl_2. The organic phase was separated and washed with H_2O (1x) and brine (1x) and dried over Na_2SO_4. The drying agent was removed by filtration and solvent was removed under reduced pressure. The product Fmoc-Asp(CyPY)OH **12** was obtained (1.24 g, 2.7 mmol, 70%) as a yellow foam.

^{1}H NMR (500 MHz, d^6-DMSO) δ 9.10 (d, J = 1.7 Hz, 2H), 8.20 (dd, J = 7.7, 1.3 Hz, 1H), 8.00–7.92 (m, 2H), 7.91–7.85 (m, 2H), 7.72 (m, 2H), 7.60 (d, J = 8.4 Hz, 1H), 7.40 (tt, J = 7.5, 1.3 Hz, 2H), 7.30 (tdd, J = 7.5, 2.1, 1.2 Hz, 2H), 4.52 (m, 1H), 4.32–4.17 (m, 3H), 2.88 (d, J = 6.3 Hz, 2H).

^{13}C NMR (126 MHz, d^6-DMSO) δ 177.69, 173.45, 155.87, 143.84, 141.43, 140.71, 140.15, 127.63, 127.24, 127.06, 125.30, 121.62, 120.10, 88.48, 65.71, 50.55, 46.63, 38.79.

HRMS (ESI): calculated for $[C_{26}H_{22}N_3O_5]^+$: m/z 456.1554 , found : 456.1554.

IR (cm^{-1}, neat): 3311, 3064, 2945, 2872, 2172, 1710,1470.

$[\alpha]_{546}^{25.54}$ (c = 0.1, CH_3OH) = – 32.9

Model peptide 15

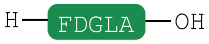

Model peptide 15 was prepared on chloro-trityl resin (loading of 0.36 mmol/g, 300 mg resin). The resin was loaded with Fmoc-Ala-OH according to the general peptide methods. The automated peptide elongation was carried out on a Syro according to general peptide methods. Fmoc-Asp(CyPY)-OH **12** was coupled manually (2 equiv, 60 min, one coupling). For the peptide cleavage, the peptide was treated with TFA/TIPS/H_2O (95:2.5:2.5, v/v) for 1 h and the resin was removed by filtration. The solution was triturated with Et_2O and centrifuged to obtain crude model peptide **15**. The crude peptide was dissolved in H_2O/DMSO (5:1, v/v) and acidified with 3 vol% conc HCl. The solution was stirred at room temperature and the CyPY deprotection was monitored by HPLC and mass spectroscopy. The crude peptide was purified by preparative HPLC (Method A). The purified peptide **15** was obtained as a white solid (24%, 14 mg).

HRMS (ESI): calculated for $[C_{24}H_{36}N_5O_8]^+$: m/z 522.2558 , found : 522.2562.

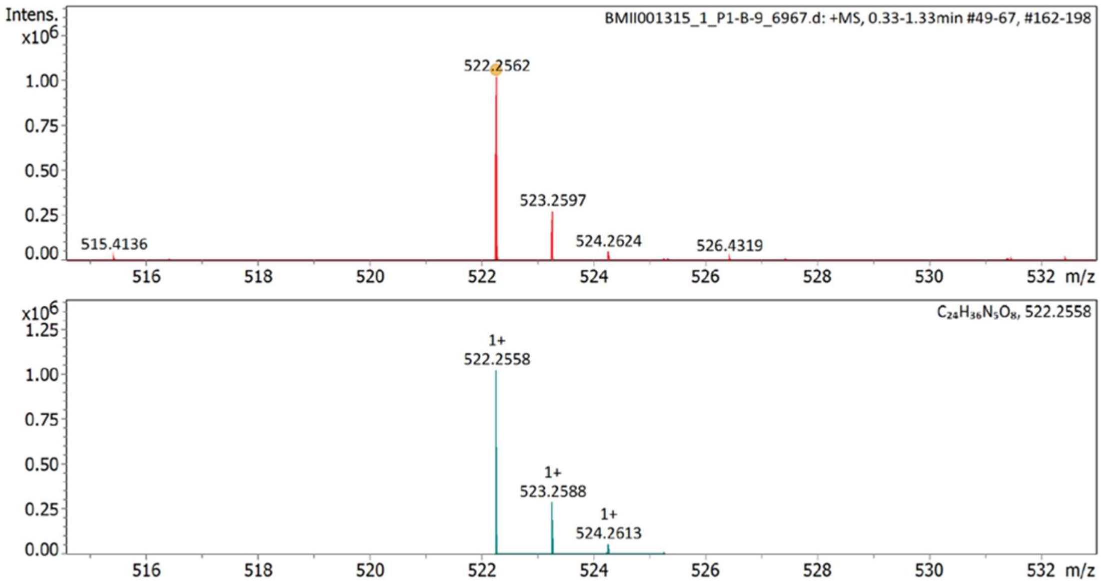

Model peptide S2

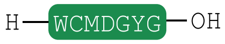

Model peptide S2 was prepared on chloro-trityl resin (loading of 0.35 mmol/g, 200 mg resin). The resin was loaded with Fmoc-Gly-OH according to the general peptide methods. The automated peptide elongation was carried out on a Syro according to general peptide methods. Fmoc-Asp(CyPY)-OH **12** was coupled manually (2 equiv, 60 min, one coupling). Fmoc-Cys(Acm)-OH was coupled manually under base free conditions (3 equiv, DIC 3 equiv, Cl-HOBt 3 equiv). For the peptide cleavage, the peptide was treated with TFA/TIPS/H_2O (95:2.5:2.5, v/v) for 1 h and the resin was removed by filtration. The solution was triturated with Et_2O and centrifuged to obtain crude model peptide **S2**. The crude peptide was dissolved in H_2O/DMSO (4:1, v/v) and acidified with 3 vol% conc HCl. The solution was stirred at room temperature and the CyPY deprotection was monitored by HPLC and mass spectroscopy. The crude peptide was purified by preparative HPLC (Method A). The purified peptide was obtained as a white solid (15%, 9 mg).

HRMS (ESI): calculated for $[C_{36}H_{47}N_8O_{11}S_2]^+$: m/z 831.2800 , found : 831.2799.

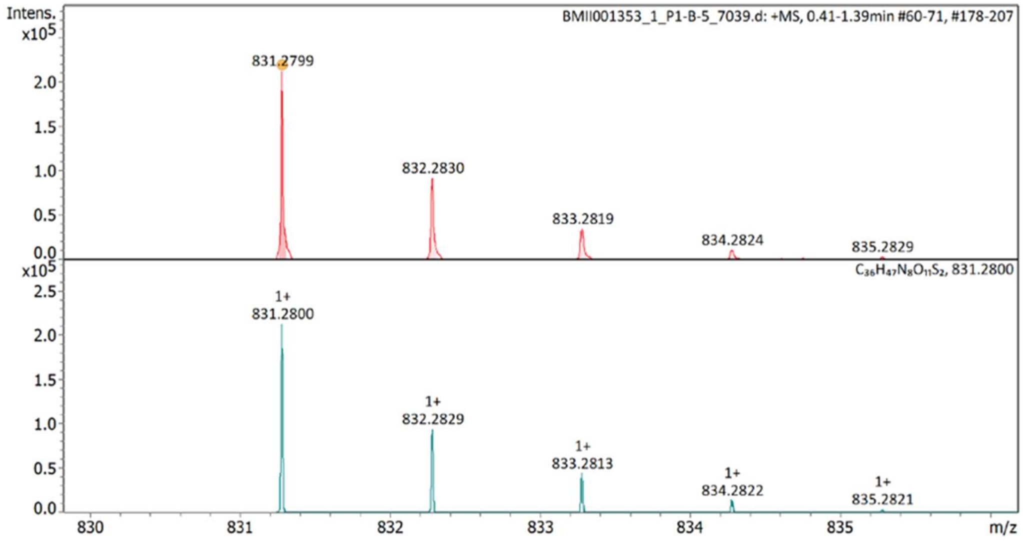

NN92 17

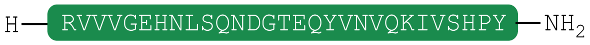

NN92 17 was prepared on rink-amide resin (loading of 0.38 mmol/g, 200 mg resin). The resin was loaded with Fmoc-Ala-OH according to the general peptide methods. The automated peptide elongation was carried out on a Syro according to general peptide methods. Fmoc-Asp(CyPY)-OH **12** was coupled manually (2 equiv, 60 min, one coupling). For the peptide cleavage, the peptide was treated with TFA/TIPS/H_2O (95:2.5:2.5, v/v) for 1 h and the resin was removed by filtration. The solution was triturated with Et_2O and centrifuged to obtain crude **NN92 17**. The crude peptide was dissolved in H_2O/DMSO (4:1, v/v) and acidified with 3 vol% conc HCl. The solution was stirred at room temperature and the CyPY deprotection was monitored by HPLC and mass spectroscopy. The crude peptide was purified by preparative HPLC (Method B) and the purified peptide **17** was obtained as a white solid (12%, 29 mg).

HRMS (ESI): calculated for $[C_{141}H_{221}N_{43}O_{44}]^+$: m/z 3220.6378 , found : 3220.6422.

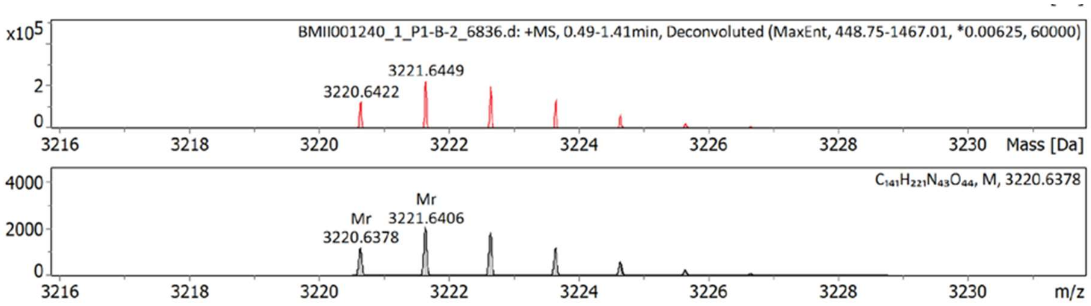

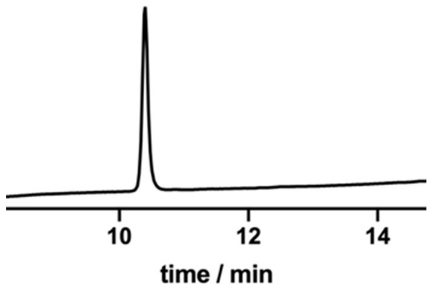

LA(Acm)$_6$-3 module 20

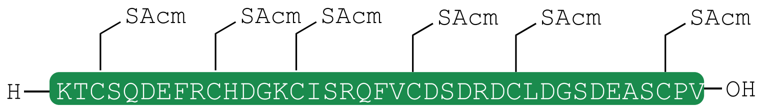

LA(Acm)$_6$-3 module 20 was prepared on chloro-trityl resin (loading of 0.30 mmol/g, 250 mg resin). The resin was loaded with Fmoc-Val-OH according to the general peptide methods. The automated peptide elongation was carried out on a Syro according to general peptide methods. Fmoc-Asp(CyPY)OH **12** was used instead of Fmoc-Asp(O^{t}Bu)OH for aspartimide prone motifs (Asp-Gly, Asp-Cys, Asp-Arg, Asp-Ser) and coupled manually (2 equiv, 60 min, one coupling). Fmoc-Cys(Acm)-OH was coupled manually under base free conditions (3 equiv, DIC 3 equiv, Cl-HOBt 3 equiv). At position 23/24 Ile-Ser was coupled as Fmoc-pseudoproline (2 equiv, 60 min, one coupling). For the peptide cleavage, the peptide was treated with TFA/DODT/H_2O (95:2.5:2.5, v/v) for 2 h and the resin was removed by filtration. The solution was triturated with Et_2O and centrifuged to obtain crude LA(Acm)-3 module **20**. The crude peptide was re-dissolved in H_2O/DMSO (5:1, v/v) and acidified with 3 vol% conc HCl. The solution was stirred at room temperature and the CyPY deprotection was monitored by HPLC and mass spectroscopy. The crude peptide was purified by preparative HPLC (Method B). The purified peptide **20** was obtained as a white solid (7%, 26 mg).

HRMS (ESI): calculated for $[C_{191}H_{302}N_{60}O_{72}S_6]^+$: m/z 4780.0139 , found : 4780.0279.

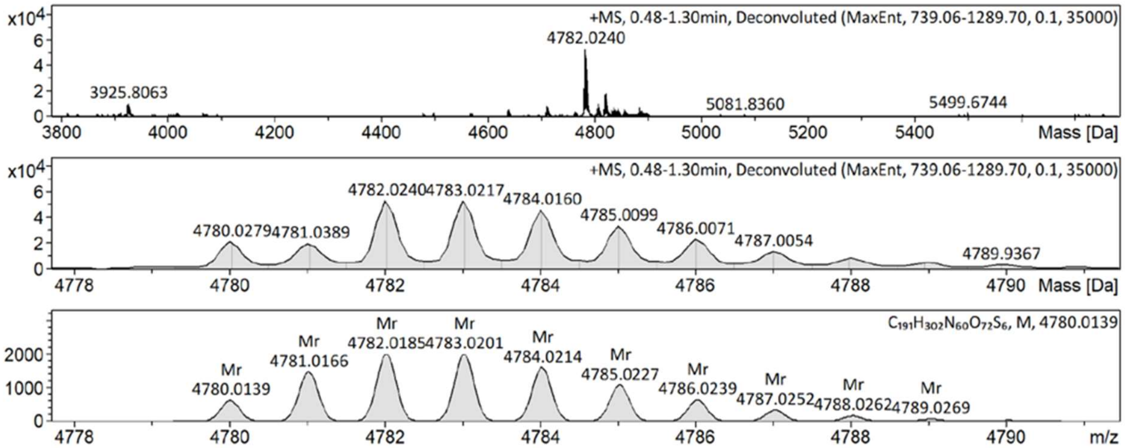

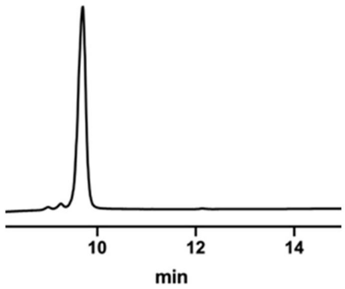

LA(Acm)$_6$-4 module 21

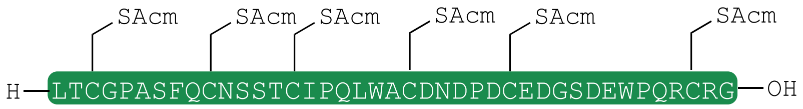

LA(Acm)$_6$-4 module **21** was prepared on chloro-trityl resin (loading of 0.29 mmol/g, 250 mg resin). The resin was loaded with Fmoc-Gly-OH according to the general peptide methods. The automated peptide elongation was carried out on a Syro according to general peptide methods. Fmoc-Asp(CyPY)OH **12** was coupled manually (2 equiv, 60 min, one coupling). Fmoc-Cys(Acm)-OH was coupled manually under base free conditions (3 equiv, DIC 3 equiv, Cl-HOBt 3 equiv). For the peptide cleavage, the peptide was treated with TFA/DODT/H_2O (95:2.5:2.5, v/v) for 2 h and the resin was removed by filtration. The solution was triturated with Et_2O and centrifuged to obtain crude LA(Acm)-3 module **21**. The crude peptide was redissolved in H_2O/DMSO (3:1, v/v) and acidified with 3 vol% conc HCl. The solution was stirred at 35 °C and the CyPY deprotection was monitored by HPLC and mass spectroscopy. The crude peptide was purified by preparative HPLC (Method A). The purified peptide **21** was obtained as a white solid (8%, 28 mg).

HRMS (ESI): calculated for $[C_{202}H_{304}N_{60}O_{73}S_6]^+$: m/z 4930.0244 , found : 4930.0657 .

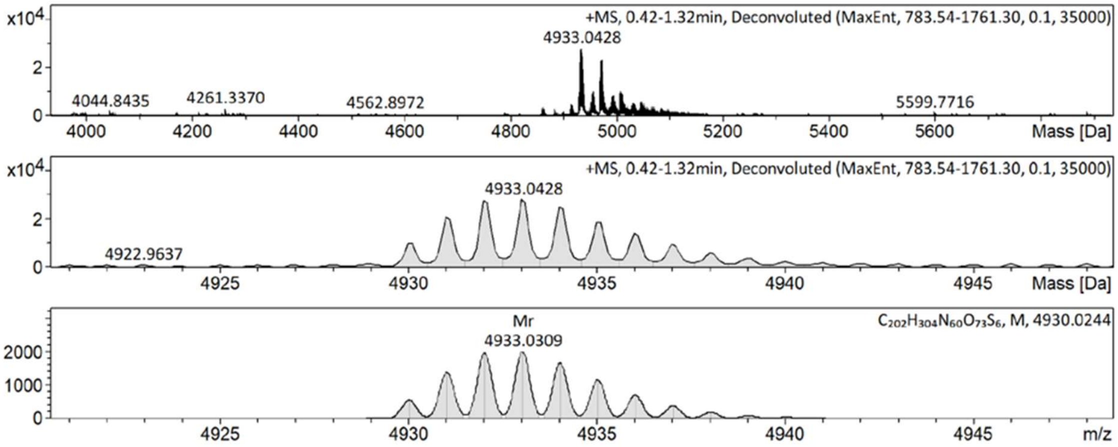

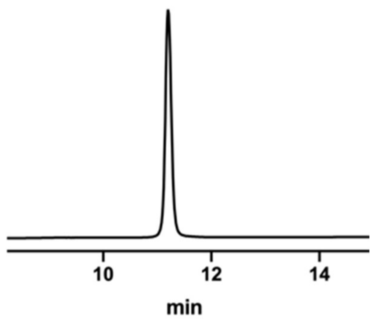

LA(SH)$_6$-3 module 22

H—KTCSQDEFRCHDGKCISRQFVCDSDRDCLDGSDEASCPV—OH

LA(Acm)$_6$-3 module **20** (4.2 µmol, 20 mg) was dissolved in AcOH/H_2O (1/1, v/v, 0.3 mM) and AgOAc was added (2 w%). The suspension was stirred in the dark at 50°C for 2h. DTT was added (1.5 equiv to AgOAc) and the precipitate was separated by centrifugation. The precipitate was washed twice with AcOH/H_2O. The aqueous solution layers were combined. The crude peptide was purified by preparative HPLC (Method A). The purified peptide LA(SH)$_6$-3 module **21** was obtained as a white solid (24%, 5 mg).

HRMS (ESI): calculated for $[C_{173}H_{272}N_{54}O_{66}S_6]^+$: m/z 4353.7912 , found : 4353.7796 .

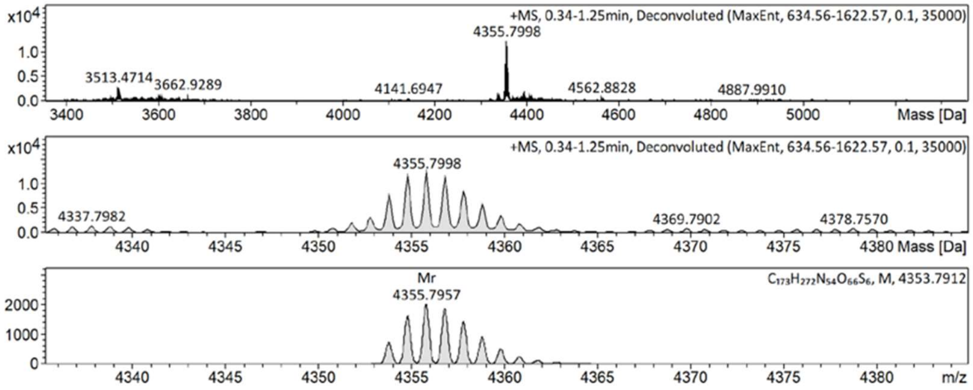

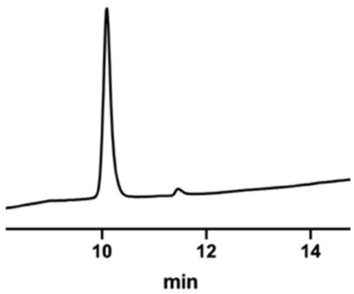

LA(SH)$_6$-4 module 23

H—LTCGPASFQCNSSTCIPQLWACDNDPDCEDGSDEWPQRCRG—OH

LA(Acm)$_6$-4 module **21** (3.0 µmol, 15 mg) was dissolved in AcOH/H_2O (1/1, v/v, 0.3 mM) and AgOAc was added (2 w%). The suspension was stirred in the dark at 50°C for 2h. DTT was added (1.5 equiv to AgOAc) and the precipitate was separated by centrifugation. The precipitate was washed twice with AcOH/H_2O. The aqueous solution layers were combined. The crude peptide was purified by preparative HPLC (Method A). The purified peptide LA(SH)$_6$-4 module **23** was obtained as a white solid (31%, 4.1 mg).

HRMS (ESI): calculated for $[C_{184}H_{274}O_{67}S_6]^+$: m/z 4503.8018 , found : 4503.8188.

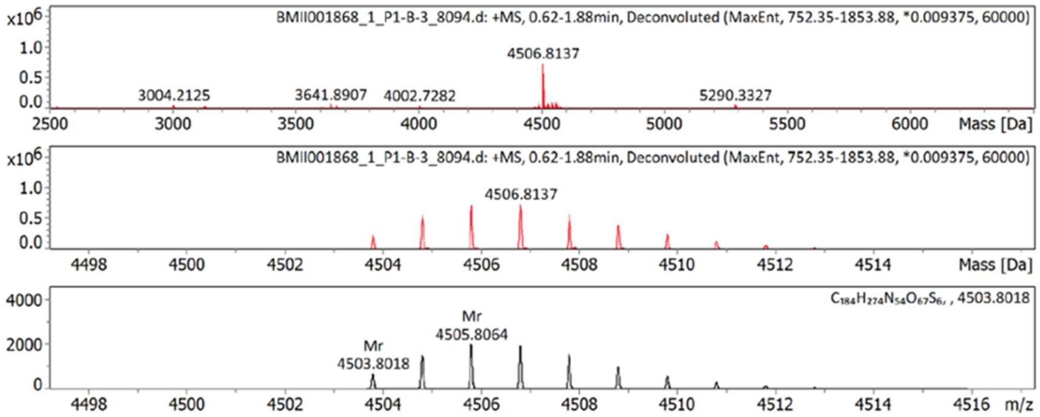

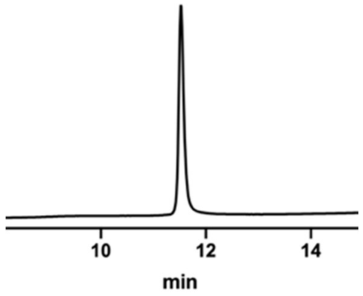

LA(SAcm)$_6$-3 ketoacid module 25

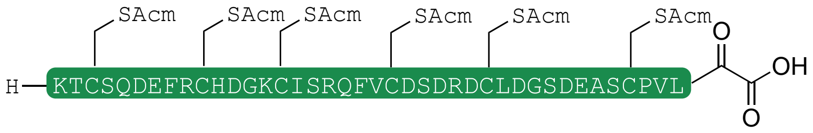

LA(SAcm)$_6$-3 alpha-ketoacid module **25** was prepared on rink-amide-resin (loading of 0.29 mmol/g, 300 mg resin). The resin was loaded with reported Fmoc-Leu-alpha-ketoacid monomer according to the general peptide methods.[2] The automated peptide elongation was carried out on a Syro according to general peptide methods. Fmoc-Asp(CyPY)OH **12** was used instead of Fmoc-Asp(O^{t}Bu)OH for aspartimide prone motifs (Asp-Gly, Asp-Cys, Asp-Arg, Asp-Ser) was coupled manually (2 equiv, 60 min, one coupling). Fmoc-Cys(Acm)-OH was coupled manually under base free conditions (3 equiv, DIC 3 equiv, Cl-HOBt 3 equiv). For the peptide cleavage, the peptide was treated with TFA/DODT/H_2O (95:2.5:2.5, v/v) for 2 h and the resin was removed by filtration. The solution was triturated with Et_2O and centrifuged to obtain crude LA(SAcm)$_6$-3 ketoacid module **25**. The crude peptide was redissolved in H_2O/DMSO (3:1, v/v) and acidified with 3 vol% conc HCl. The solution was stirred at room temperature and the CyPY deprotection was monitored by HPLC and mass spectroscopy. The crude peptide was purified by preparative HPLC (Method A). The purified peptide **25** was obtained as a white solid (7%, 31 mg).

HRMS (ESI): calculated for $[C_{198}H_{313}N_{61}O_{74}S_6]^+$: m/z 4921.0929 , found : 4921.1215 .

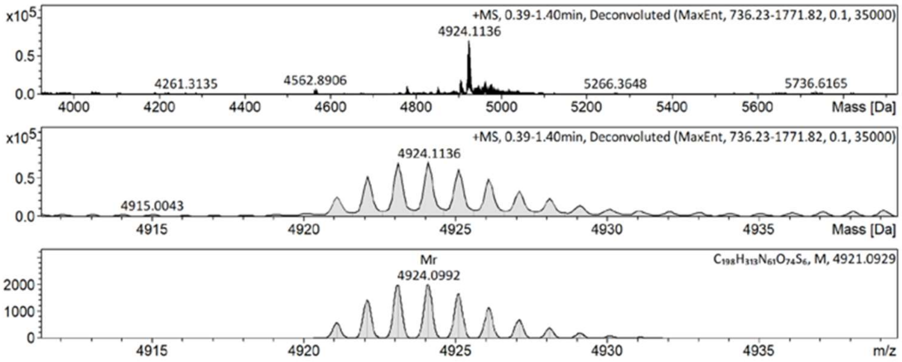

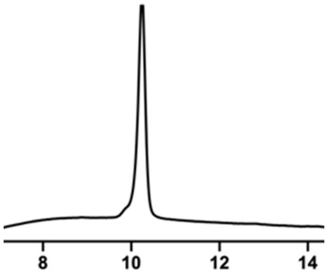

LA(SAcm)$_6$-4 hydroxylamine module 26

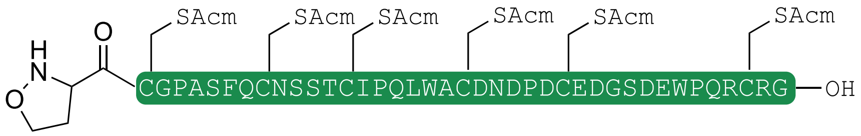

LA(SAcm)$_6$-4 hydroxylamine module **26** was prepared on chloro-trityl resin (loading of 0.28 mmol/g, 180 mg resin). The resin was loaded with Fmoc-Gly-OH according to the general peptide methods. The automated peptide elongation was carried out on a Syro according to general peptide methods. Fmoc-Asp(CyPY)-OH **12** was coupled manually (2 equiv, 60 min, one coupling). Fmoc-Cys(Acm)-OH was coupled manually under base free conditions (3 equiv, DIC 3 equiv, Cl-HOBt 3 equiv). N-Boc-oxaproline was coupled manually (3 equiv, 60 min, one coupling). For the peptide cleavage, the peptide was treated with TFA/DODT/H_2O (95:2.5:2.5, v/v) for 2 h and the resin was removed by filtration. The solution was triturated with Et_2O and centrifuged to obtain crude LA(SAcm)$_6$-4 hydroxylamine module **26**. The crude peptide was dissolved in H_2O/DMSO (4:1, v/v) and acidified with 3 vol% conc HCl. The solution was stirred at 35 °C and the CyPY deprotection was monitored by HPLC and mass spectroscopy. The crude peptide was purified by preparative HPLC (Method A). The purified peptide **26** was obtained as a white solid (12%, 27 mg).

HRMS (ESI): calculated for $[C_{196}H_{291}N_{59}O_{72}S_6]^+$: m/z 4814.9247 , found : 4814.9261 .

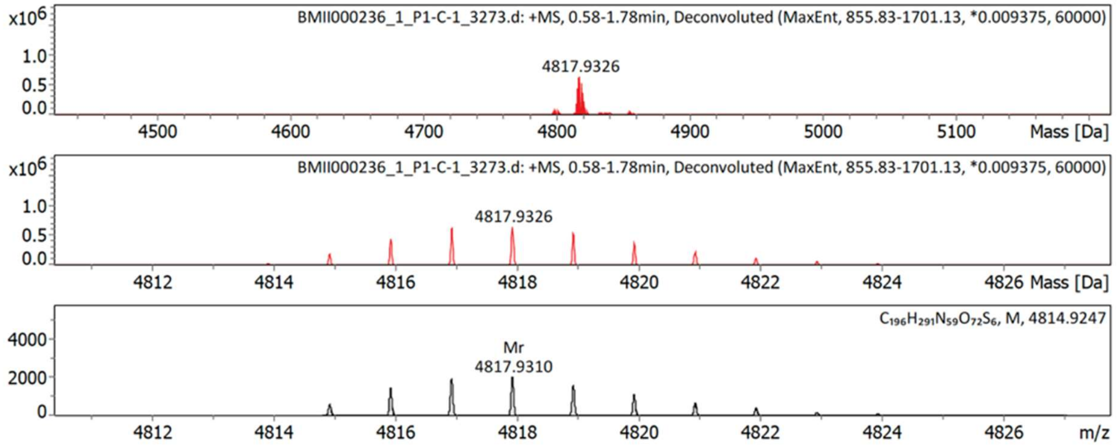

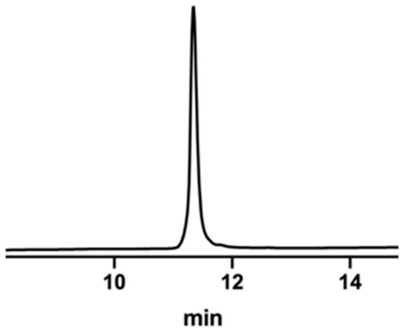

LA(SAcm)$_{12}$-3-4 module 27

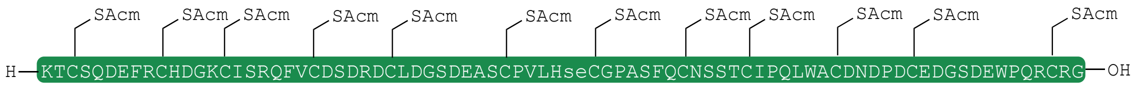

LA(SAcm)$_6$-4 hydroxylamine module **26** (5.2 µmol, 25 mg, 1.1 equiv) and LA(SAcm)$_6$-3 alpha-ketoacid **25** (5.2 µmol, 23 mg, 1.0 equiv) were dissolved in DMSO/H_2O (9/1, v:v, 18 mM) with 0.1 M oxalic acid. The solution was heated to 60 °C and stirred for 12h. The reaction was monitored with HPLC. After 12 h the solution was diluted in sodium carbonate buffer (pH 9.5, 3 ml) and stirred for 90 min at room temperature. The solution was acidified and purified by preparative HPLC (Method A). LA(SAcm)$_{12}$-3-4 module **27** was obtained as white solid (26%, 12 mg).

HRMS (ESI): calculated for $[C_{393}H_{604}N_{120}O_{144}S_{12}]^+$: m/z 9692.0278 , found : 9692.0146.

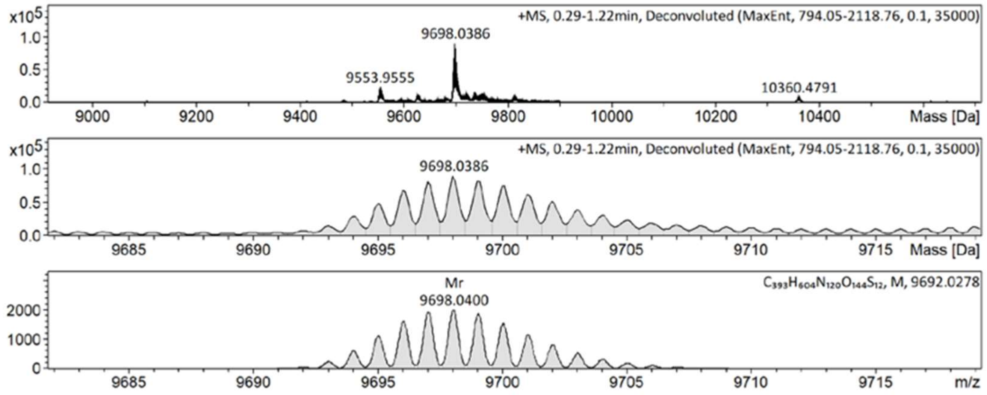

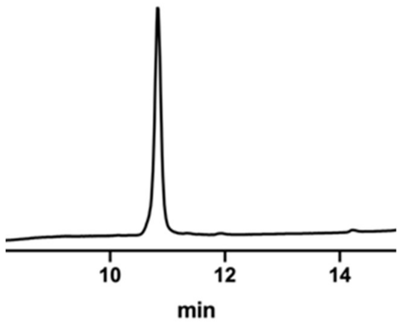

LA(SH)$_{12}$-3-4 module 28

H—KTCSQDEFRCHDGKCISRQFVCDSDRDCLDGSDEASCPVLHseCGPASFQCNSSTCIPQLWACDNDPDCEDGSDEWPQRCRG—OH

LA(SAcm)$_{12}$-3-4 module **27** (0.8 μmol, 8 mg) was dissolved in AcOH/H_2O (1/1, v/v, 0.3 mM) and AgOAc was added (20 mg). The suspension was stirred in the dark at 50°C for 2h. DTT was added (1.5 equiv of AgOAc) and the precipitate was separated by centrifugation. The precipitate was washed twice with AcOH/H_2O (1/1, v/v). The supernatants were combined. The crude peptide was purified by preparative HPLC (Method A). The purified peptide LA(SH)$_{12}$-3-4 module **28** was obtained as a white solid (40%, 3 mg).

HRMS (ESI): calculated for $[C_{357}H_{544}N_{108}O_{132}S_{12}]^+$: m/z 8839.5818 , found : 8839.5879.

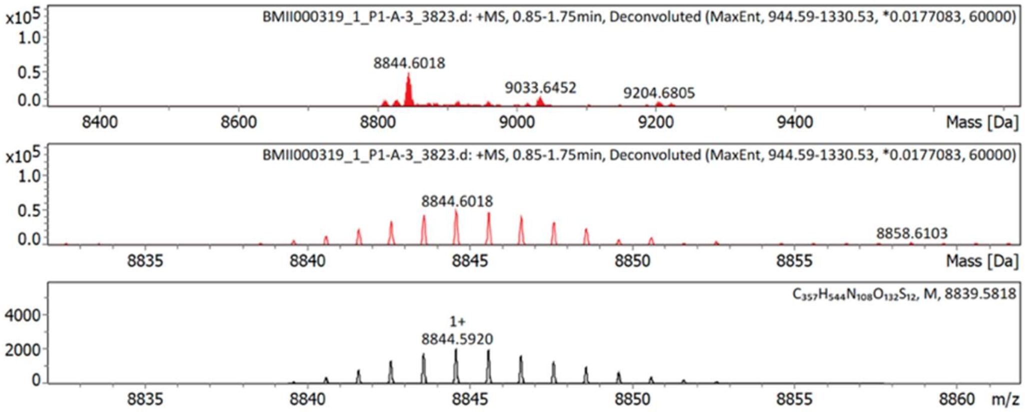
BMII000319_1_P1-A-3_3823.d: +MS, 0.85-1.75min, Deconvoluted (MaxEnt, 944.59-1330.53, *0.0177083, 60000)
x10^5
8844.6018
9033.6452
9204.6805
Mass [Da]
BMII000319_1_P1-A-3_3823.d: +MS, 0.85-1.75min, Deconvoluted (MaxEnt, 944.59-1330.53, *0.0177083, 60000)
8844.6018
8858.6103
Mass [Da]
C357H544N108O132S12, M, 8839.5818
1+
8844.5920
m/z

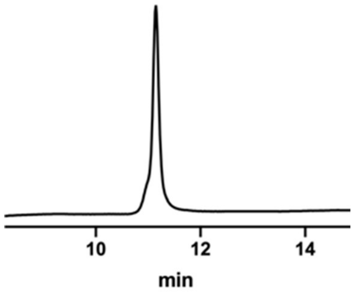
10
12
14
min

12. NMR Spectra

Hydrocinnamic acid cyanopyridiniumylide 1

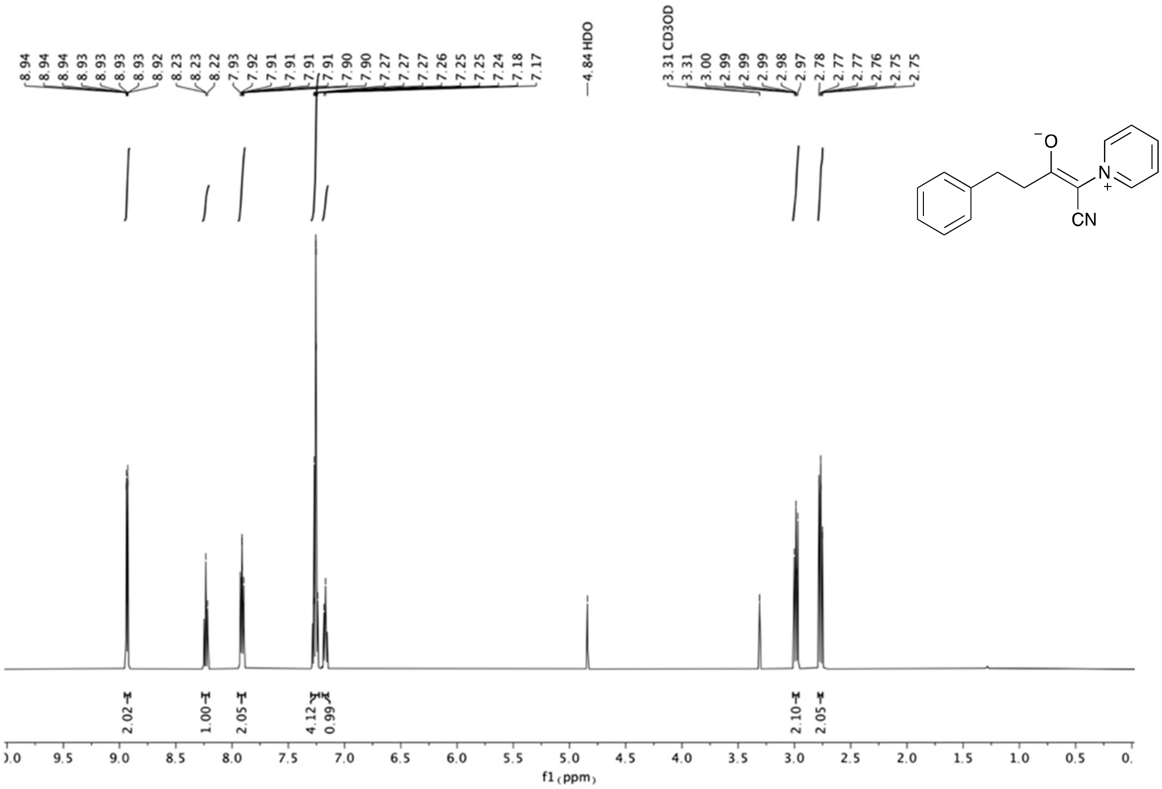

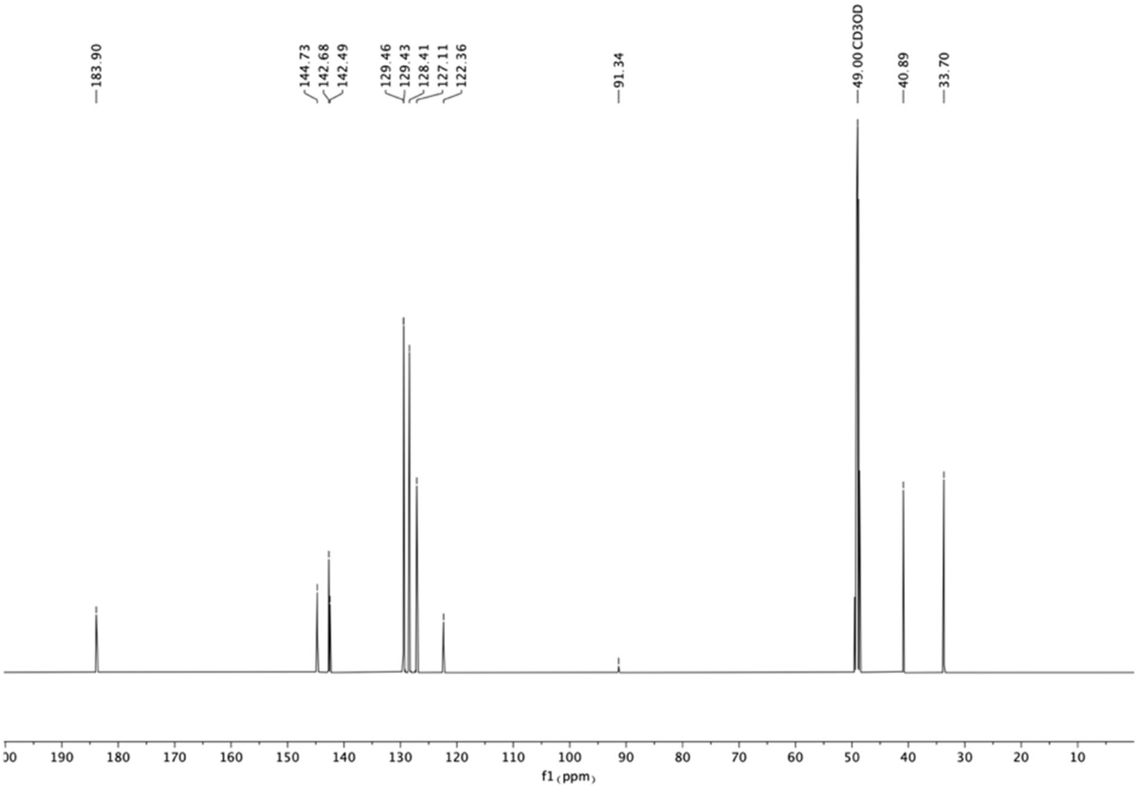

Hydrocinnamic acid cyano(4-methoxy)pyridiniumylide 2

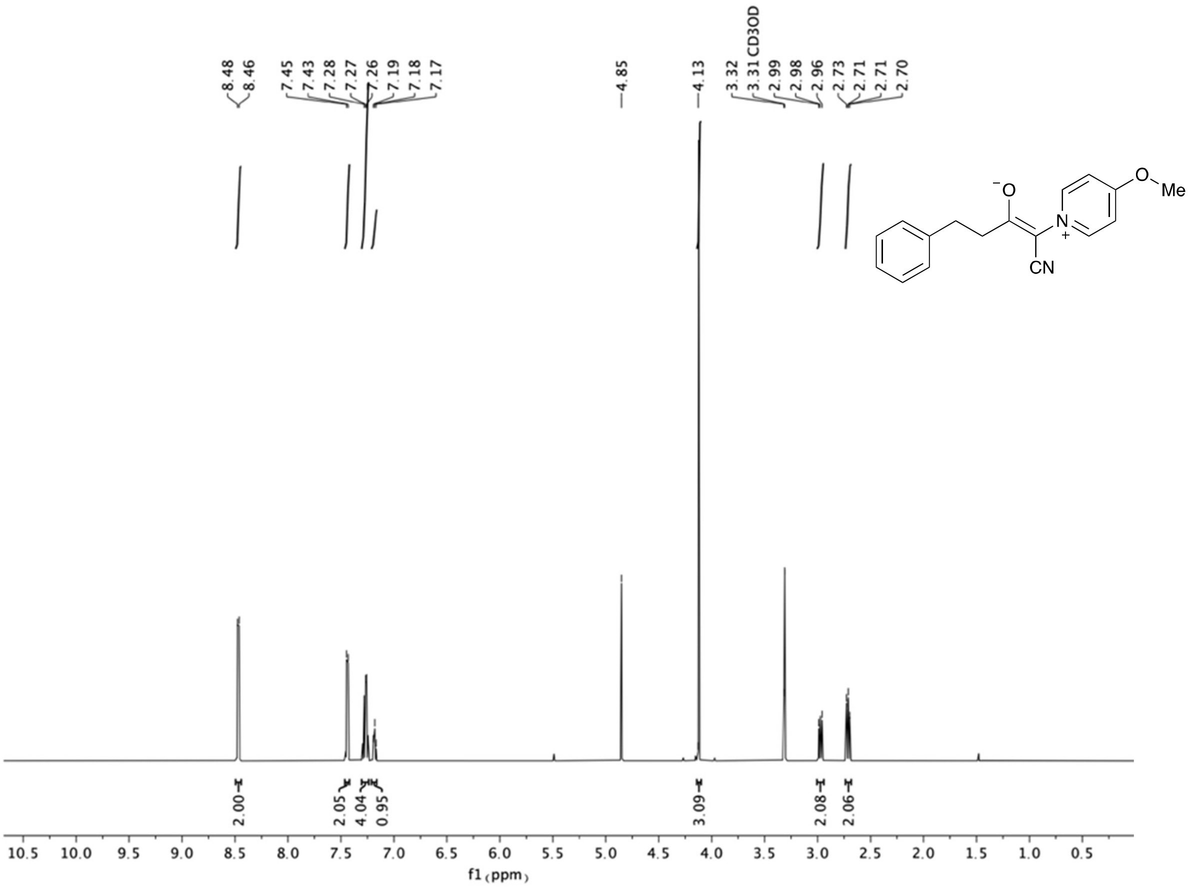

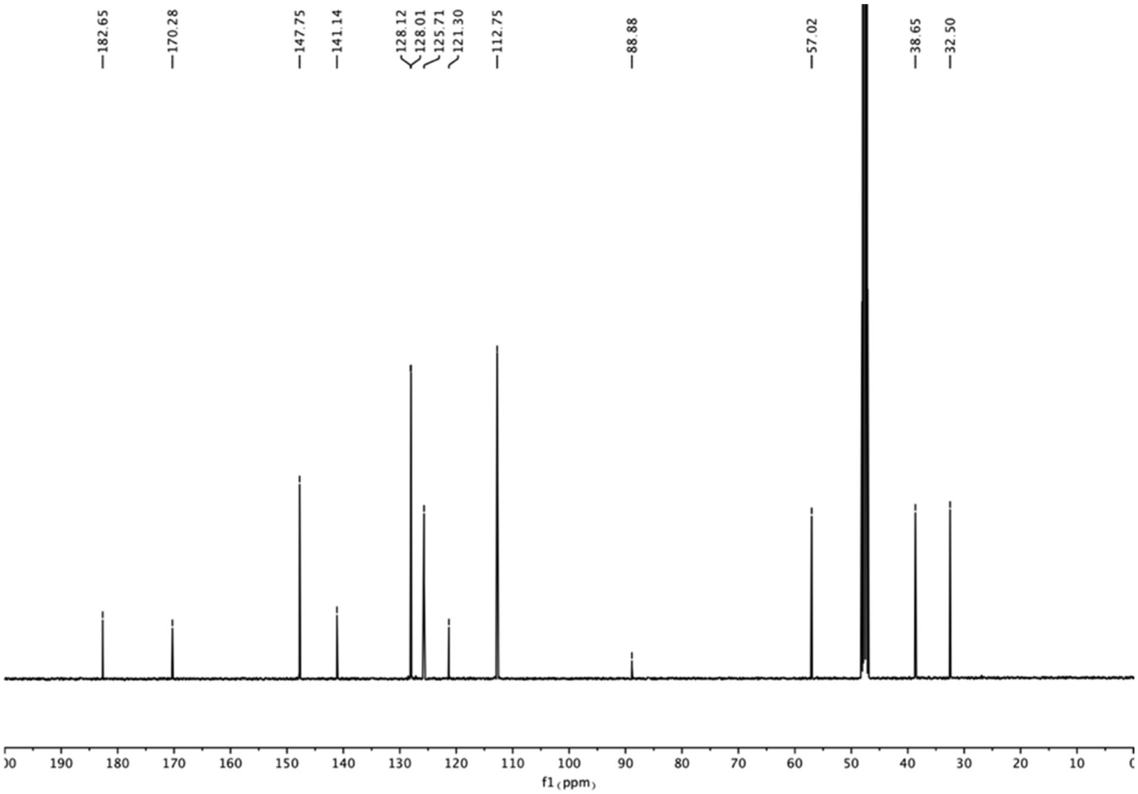

Hydrocinnamic acid cyano[3-(methyl-amide)]pyridiniumylide 3

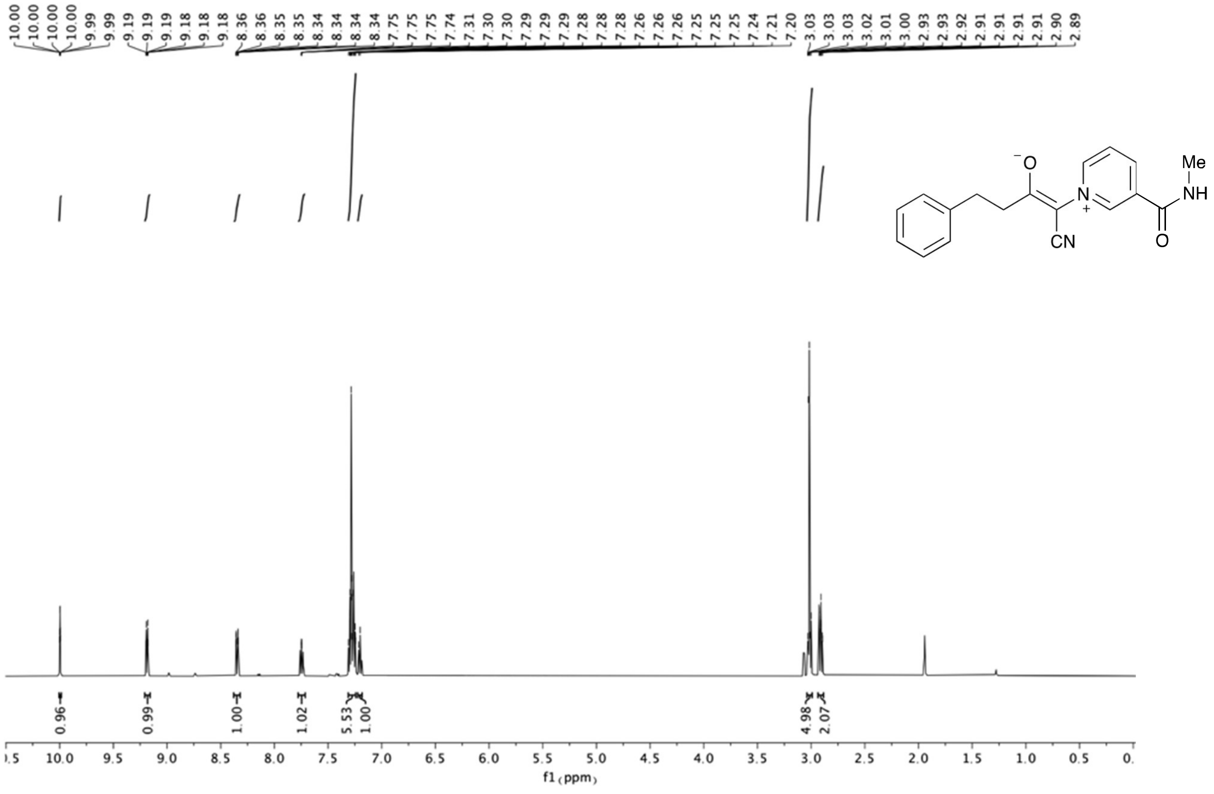

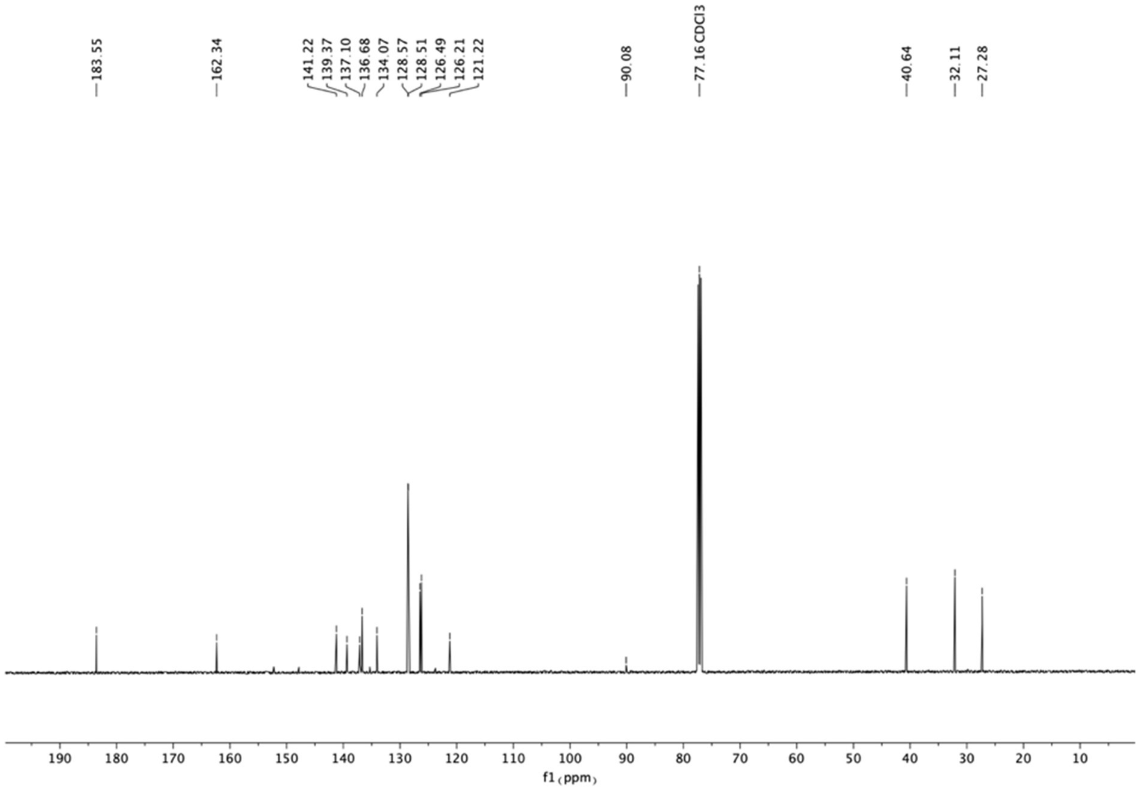

Hydrocinnamic acid cyano(3-chloro)pyridiniumylide 4

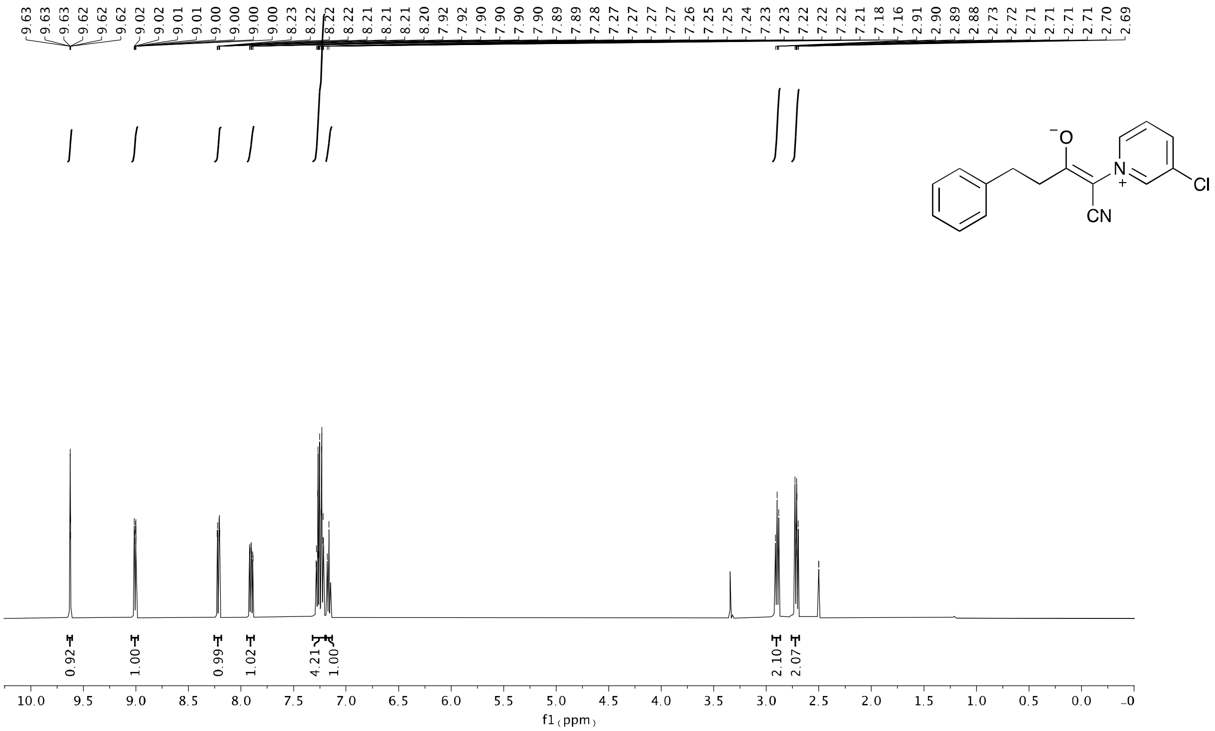

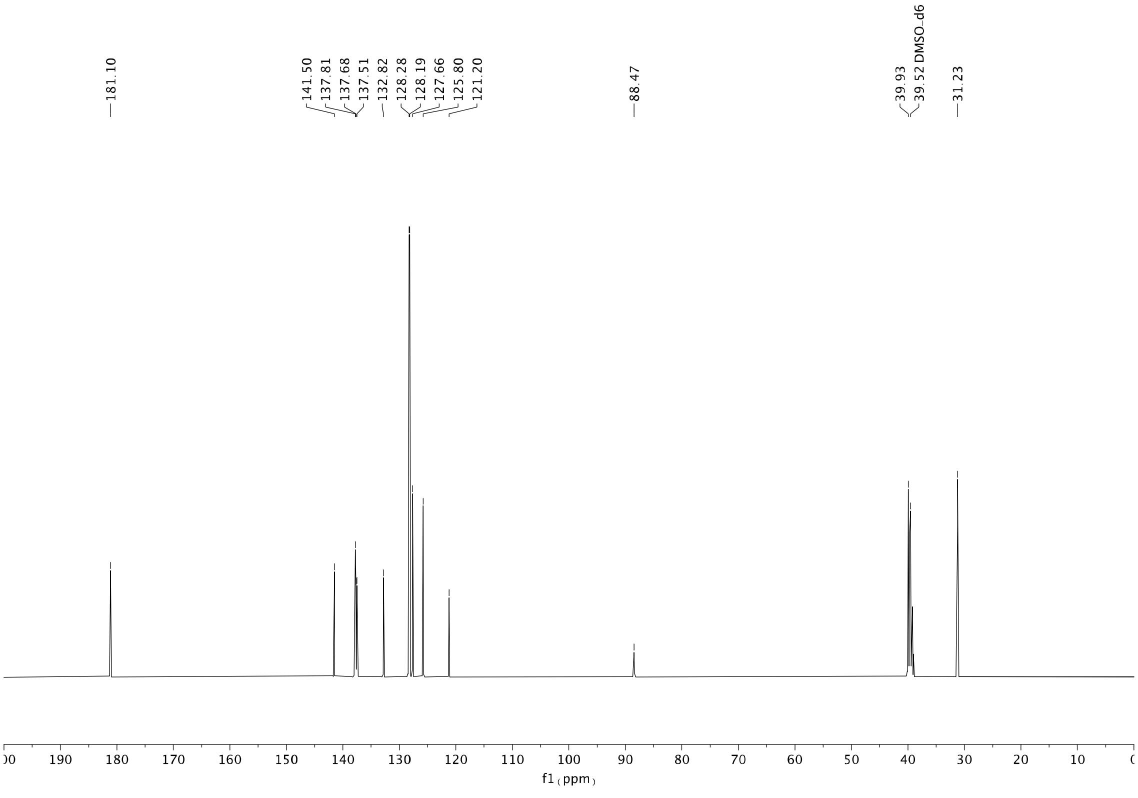

Hydrocinnamic acid cyano(4-phenyl)pyridiniumylide 5

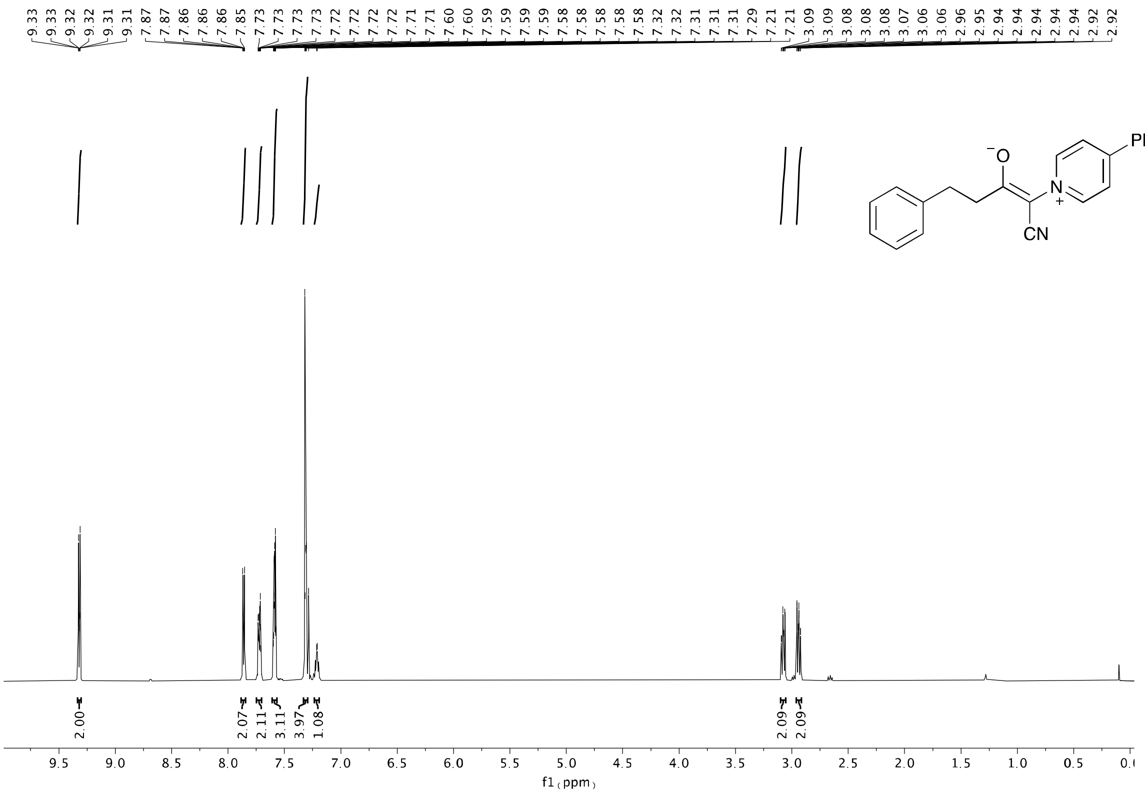

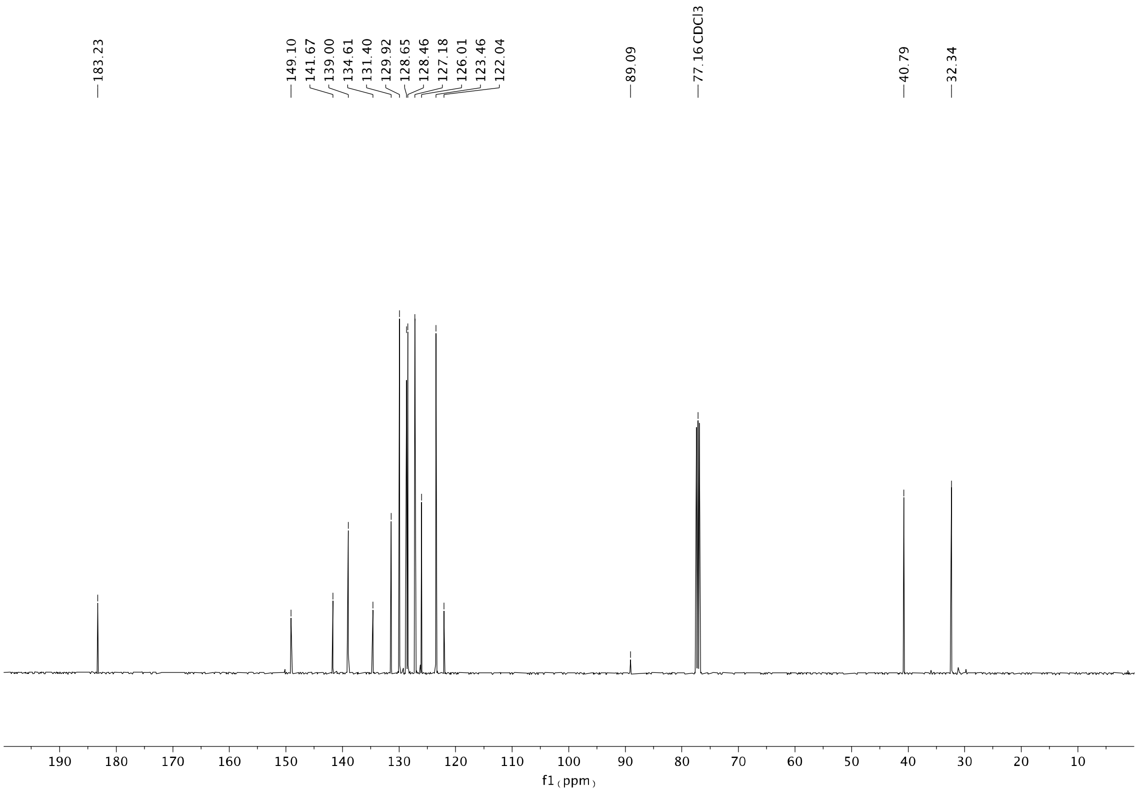

N-acetonitrile-pyridinium bromide 7

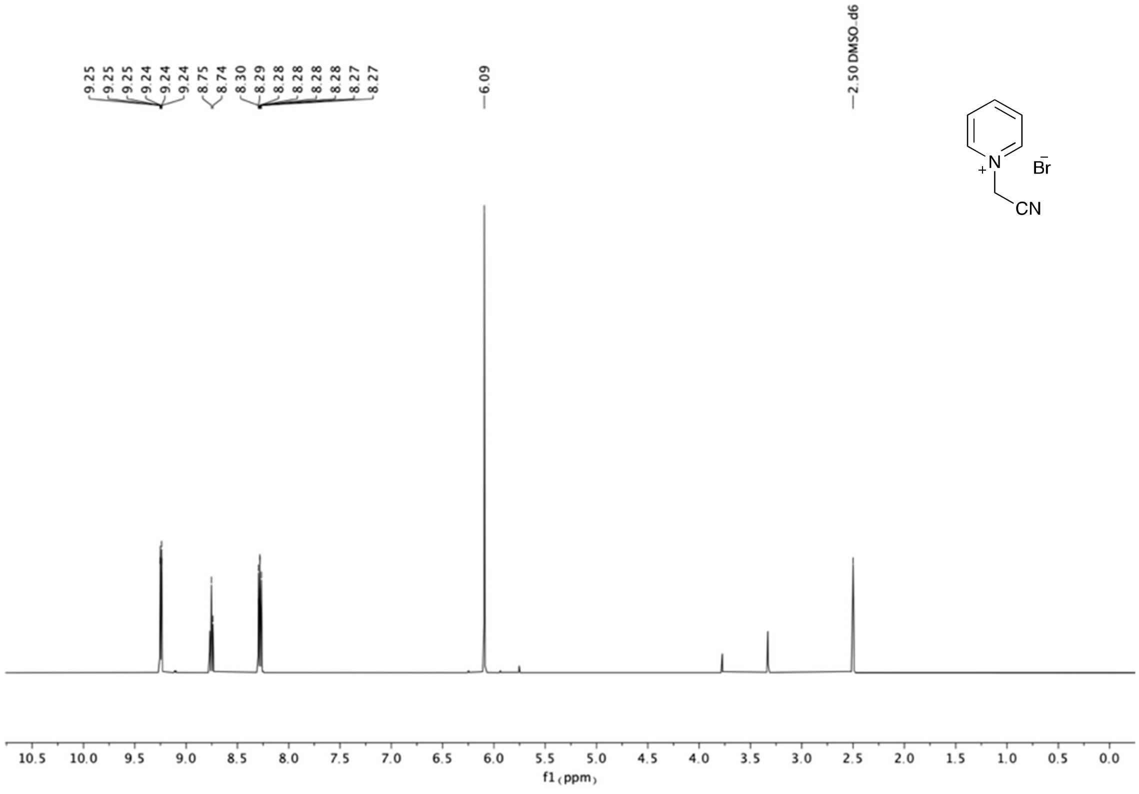

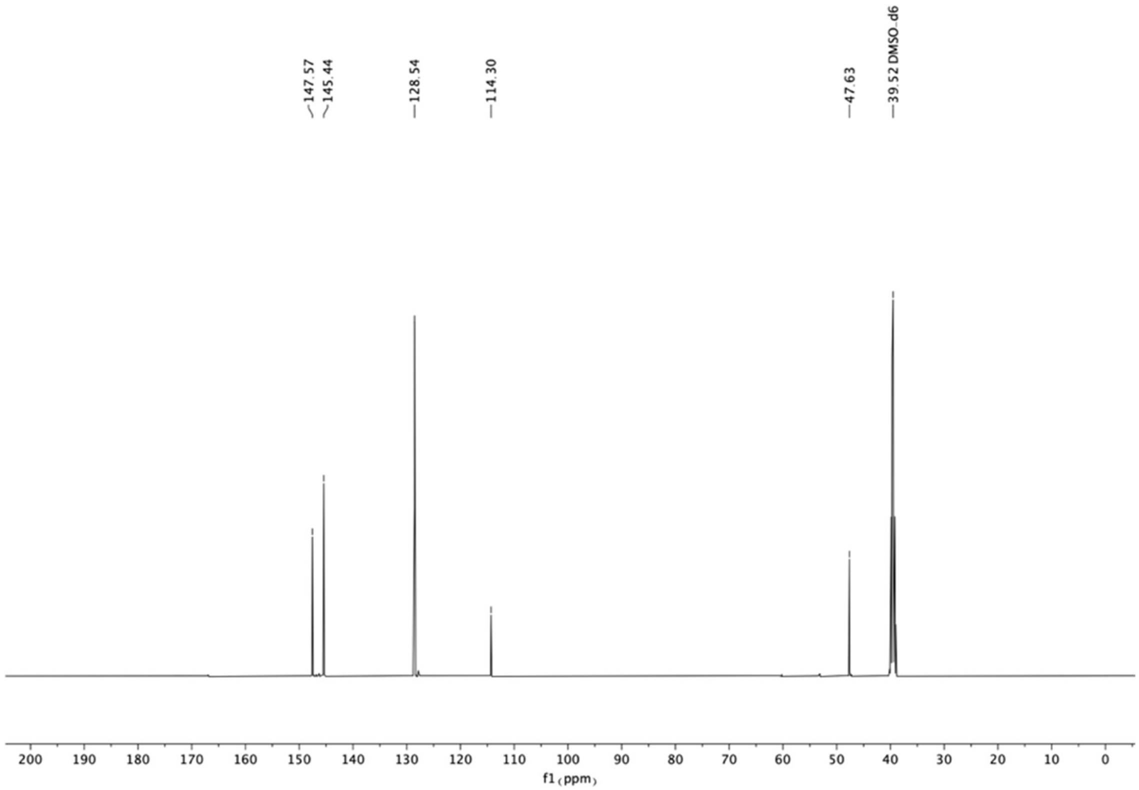

N-acetonitrile-(4-methoxy)pyridinium bromide 8

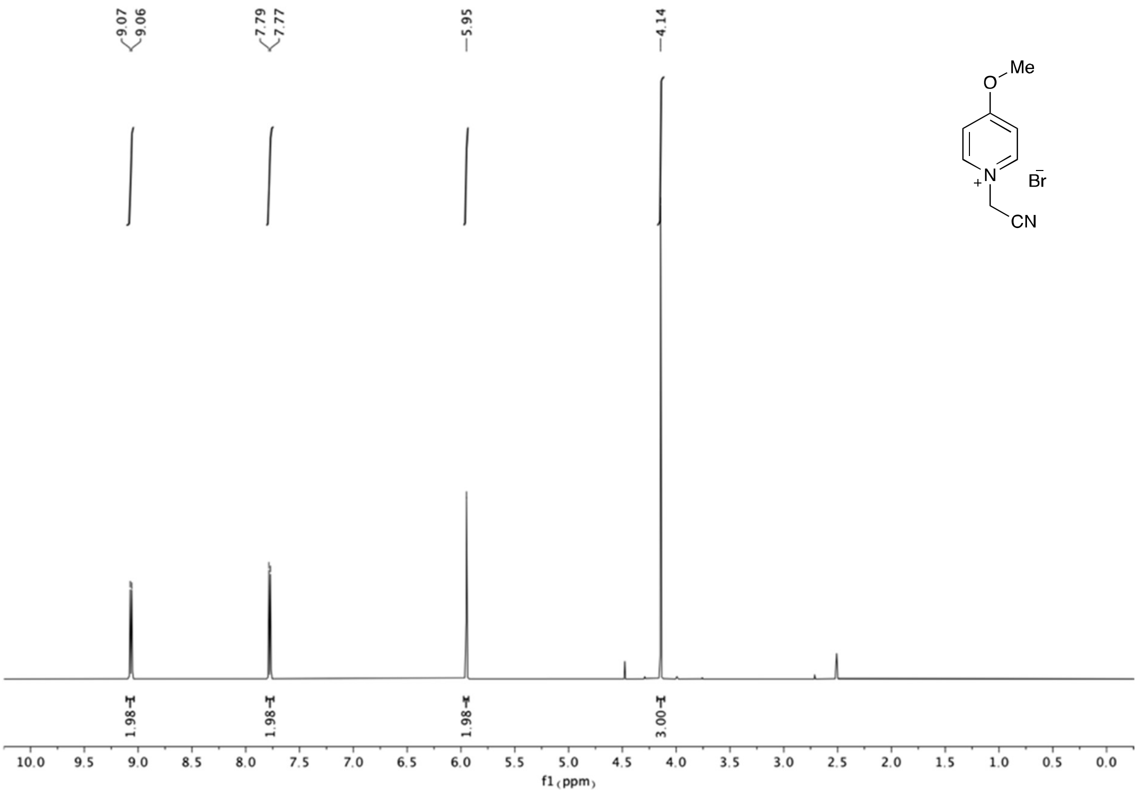

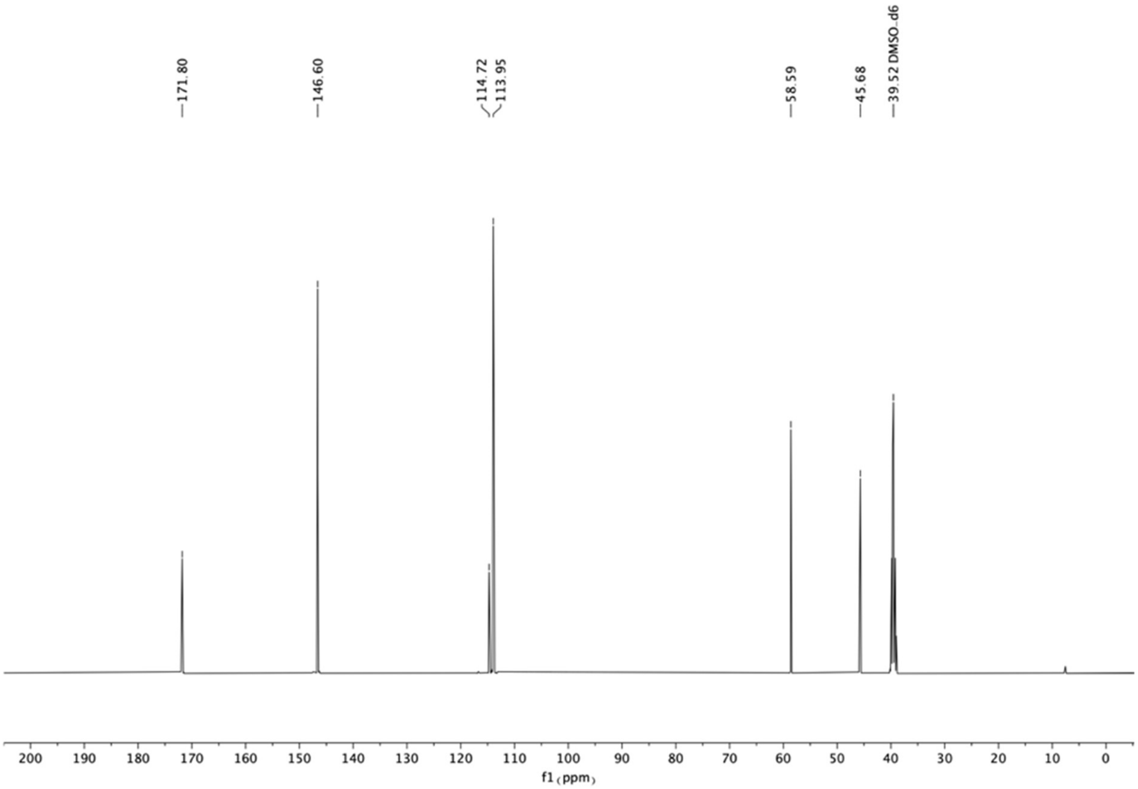

N-Acetonitrile-[3-(methyl-amide)]pyridinium Bromide 9

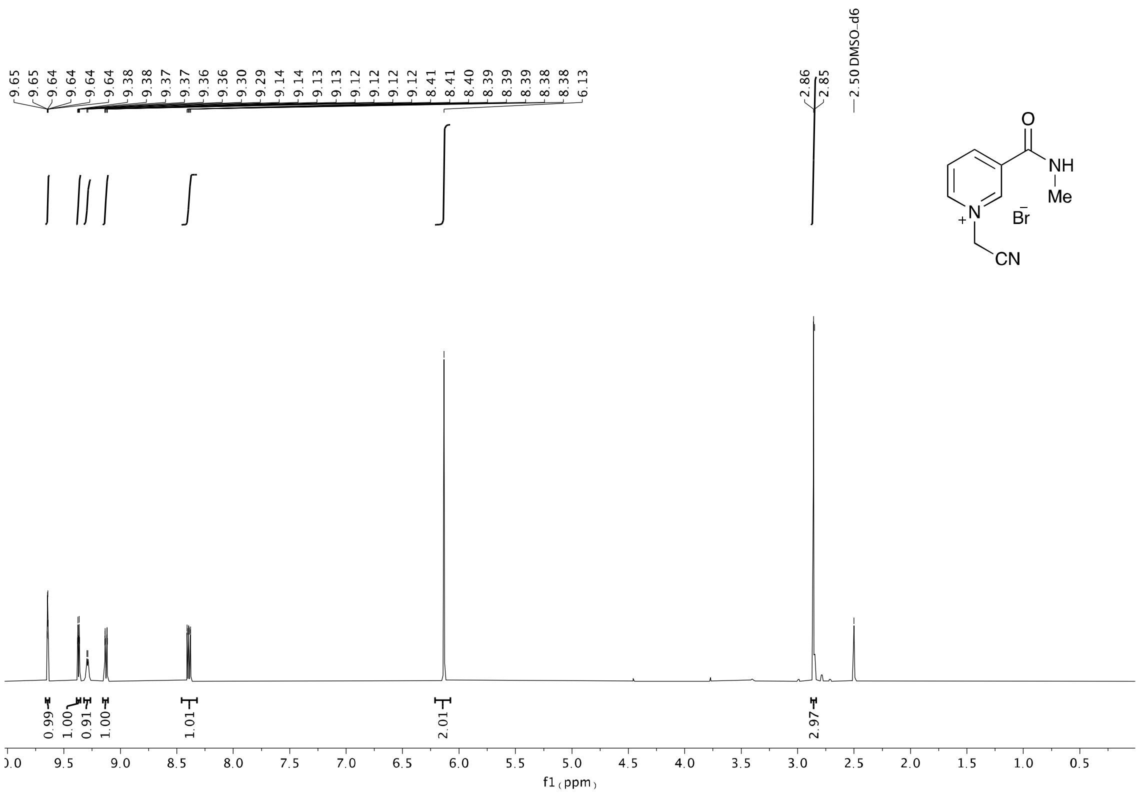

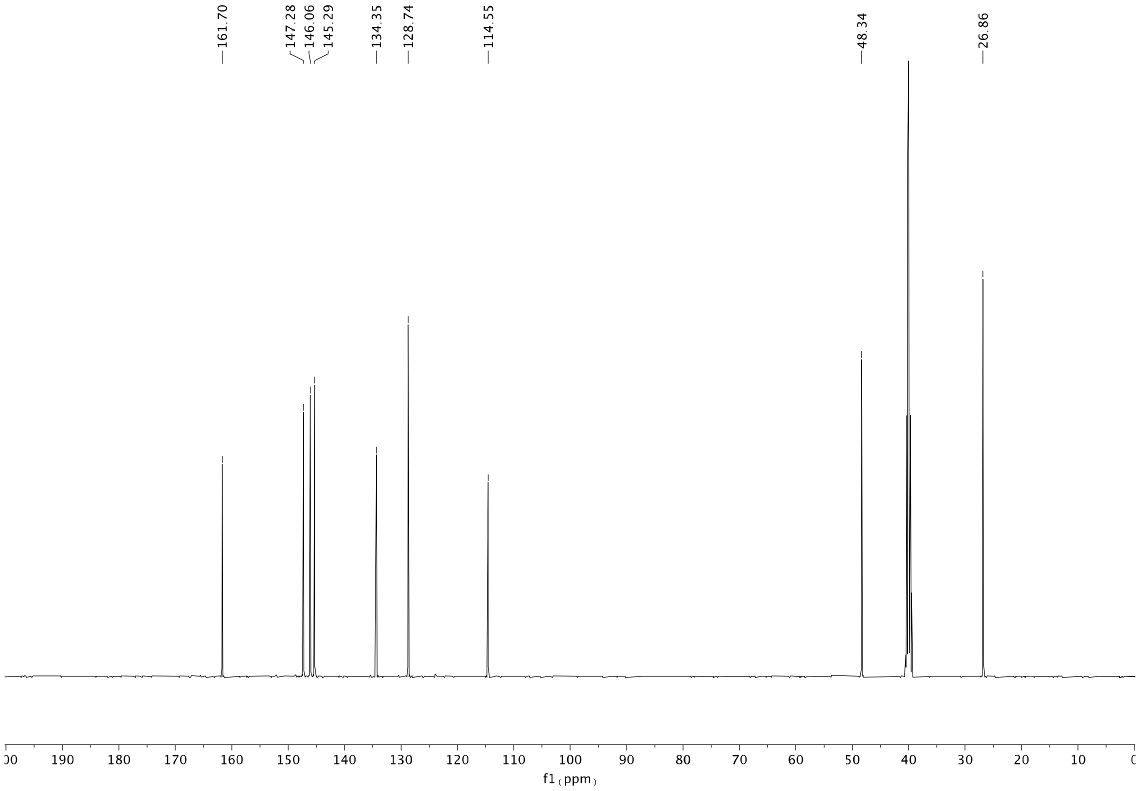

N-acetonitrile-(3-chloro)pyridinium bromide 10

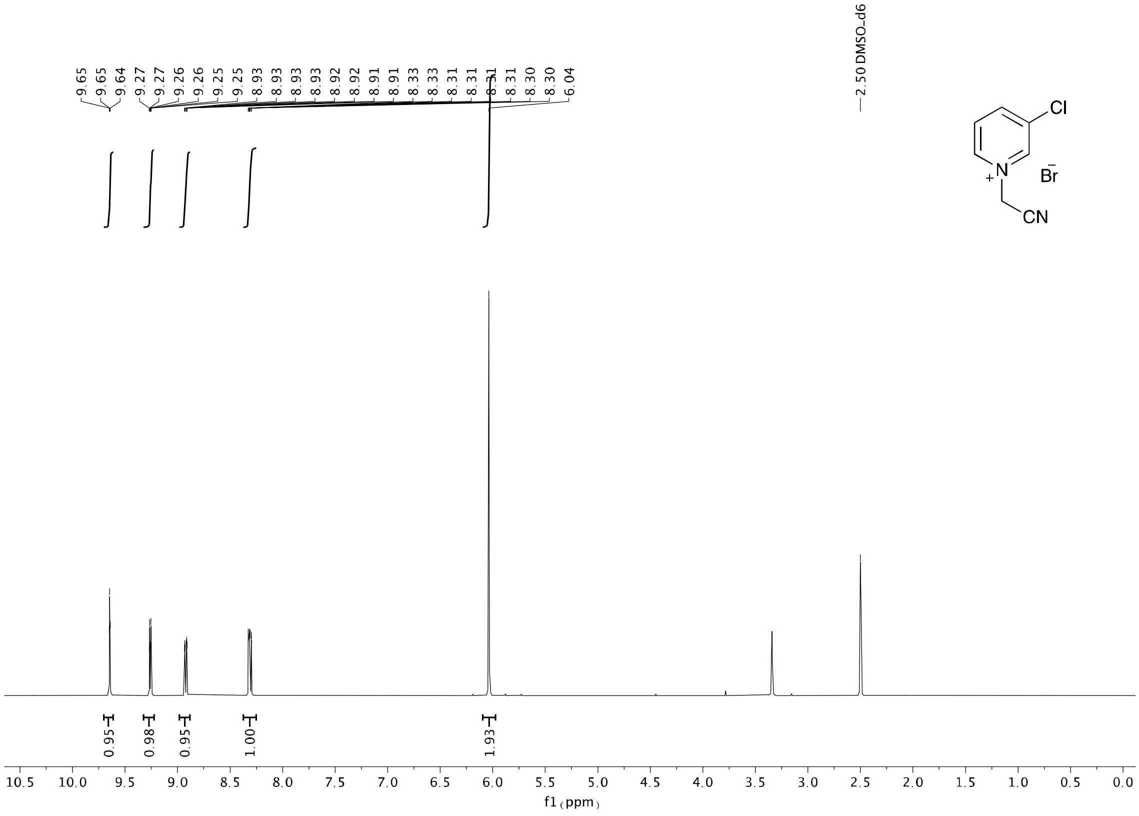

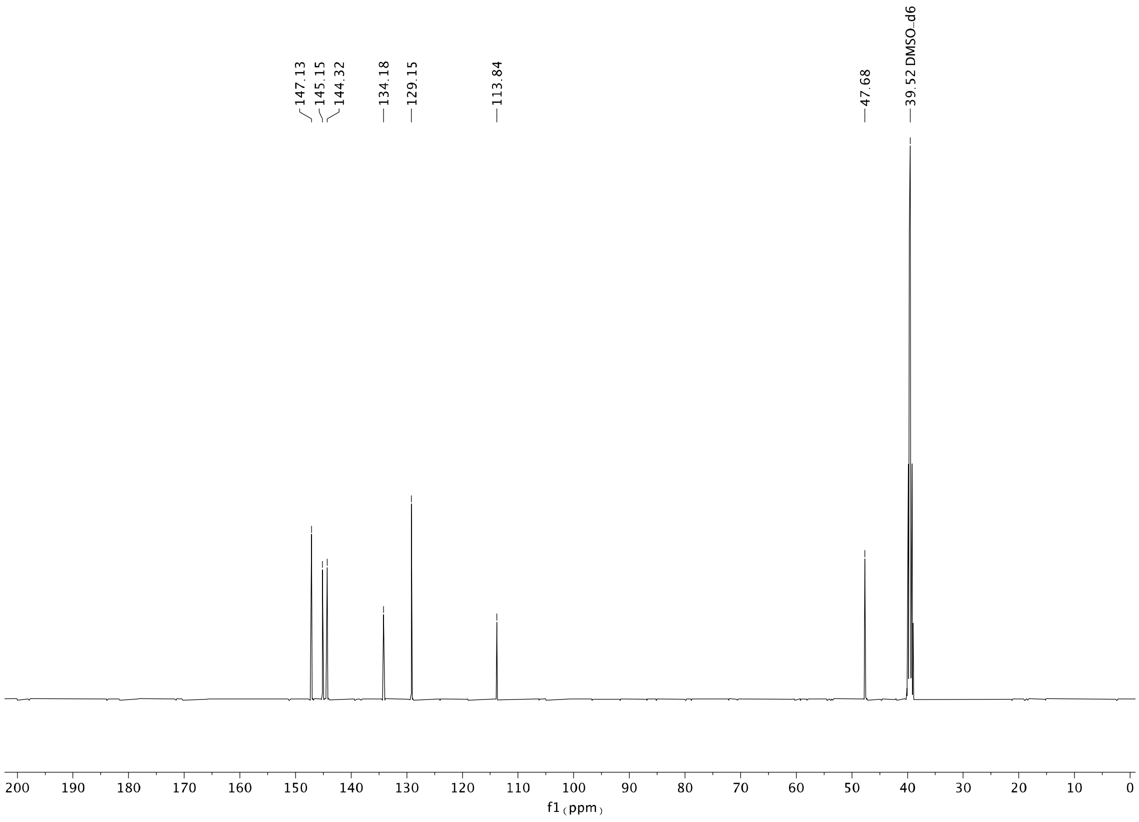

_N_-acetonitrile-(4-phenyl)pyridinium bromide 11

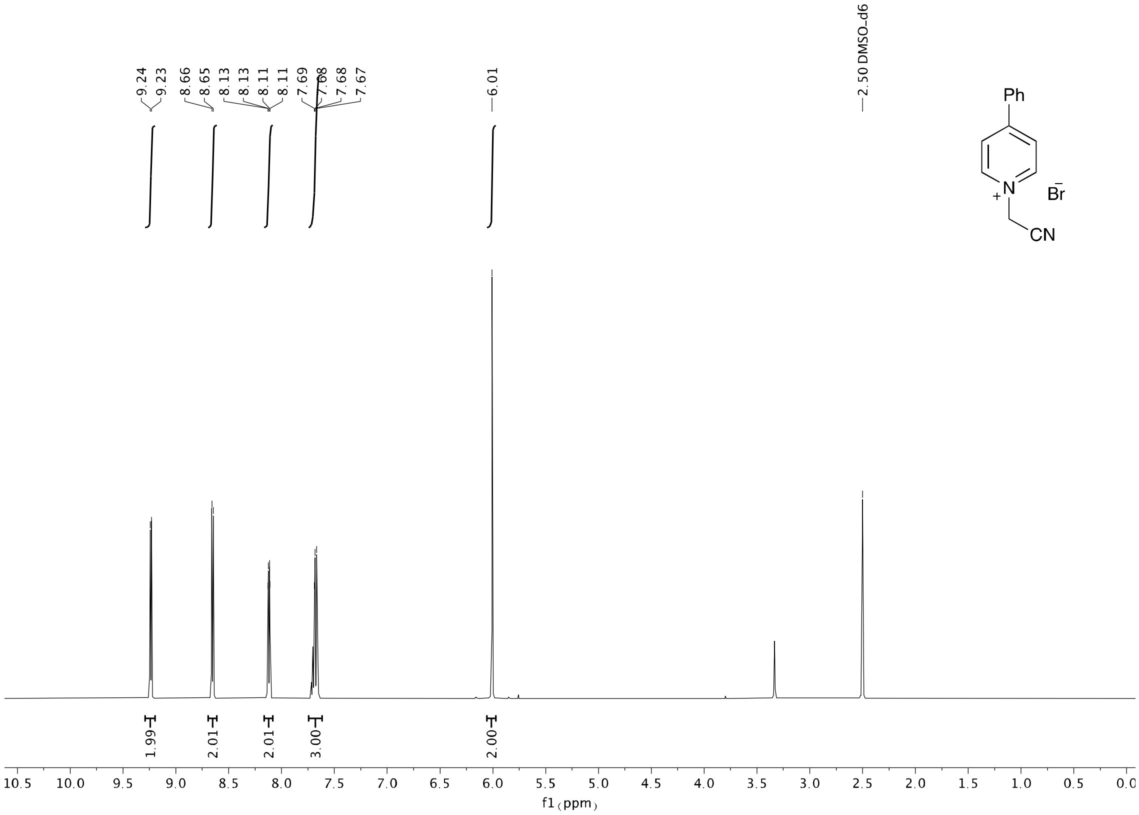

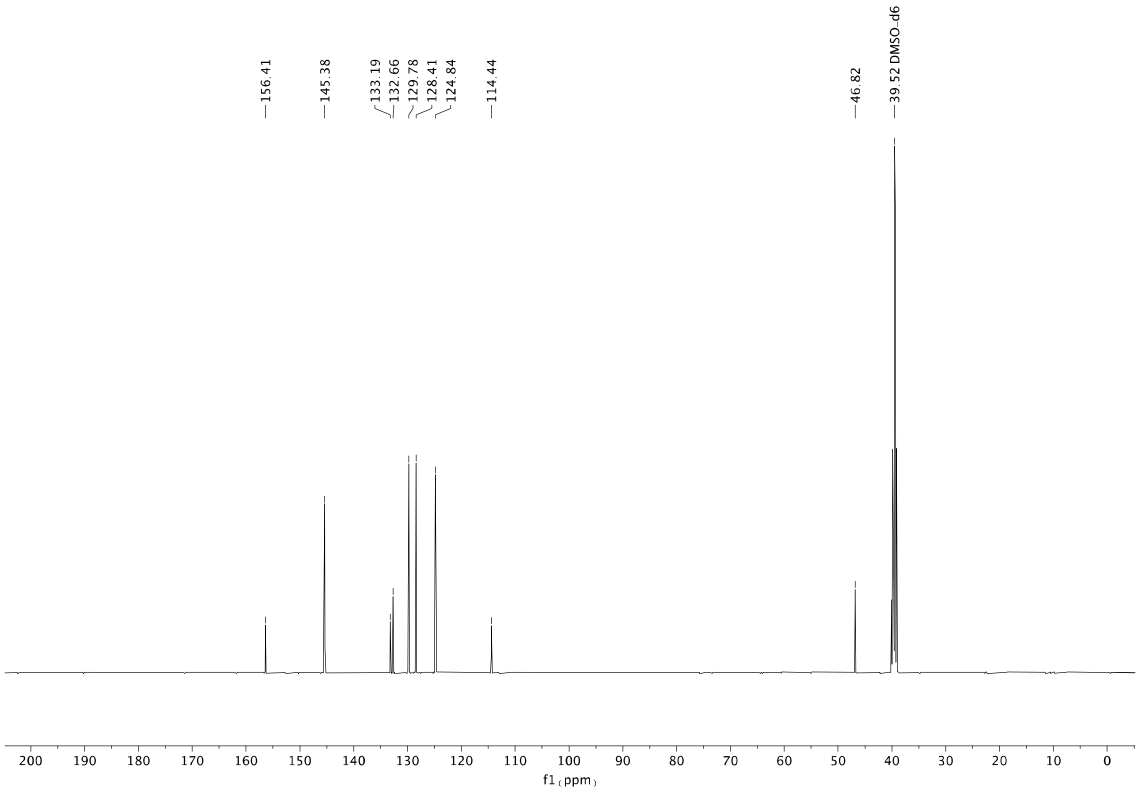

Fmoc-Asp(CyPY)-O^{t}Bu 13

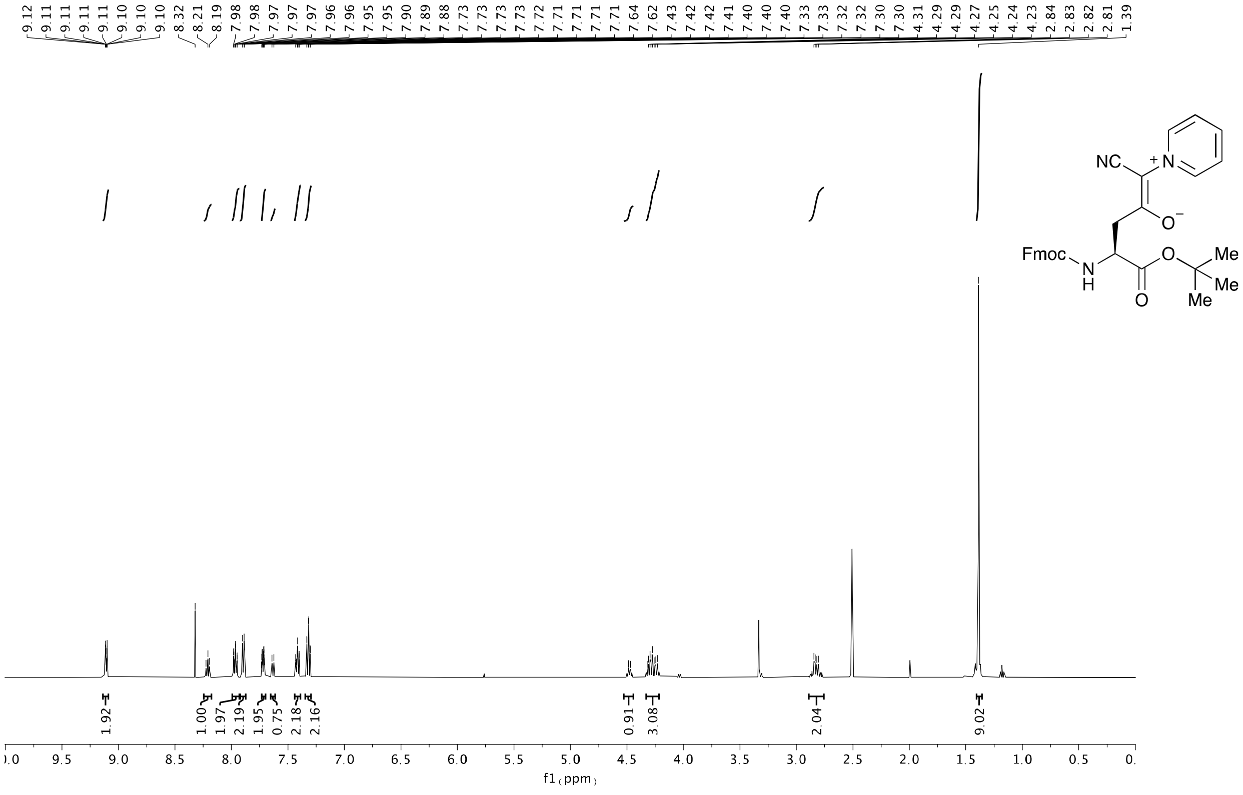

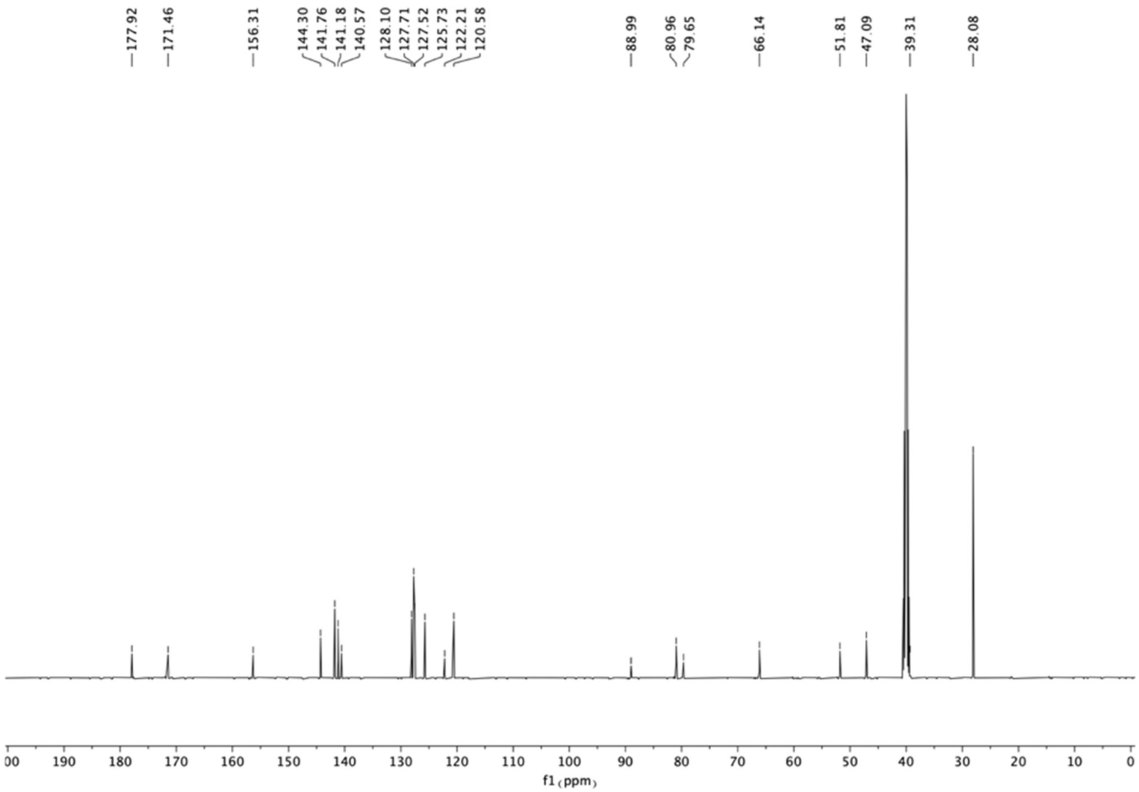

Fmoc-Asp(CyPY)-OH 12

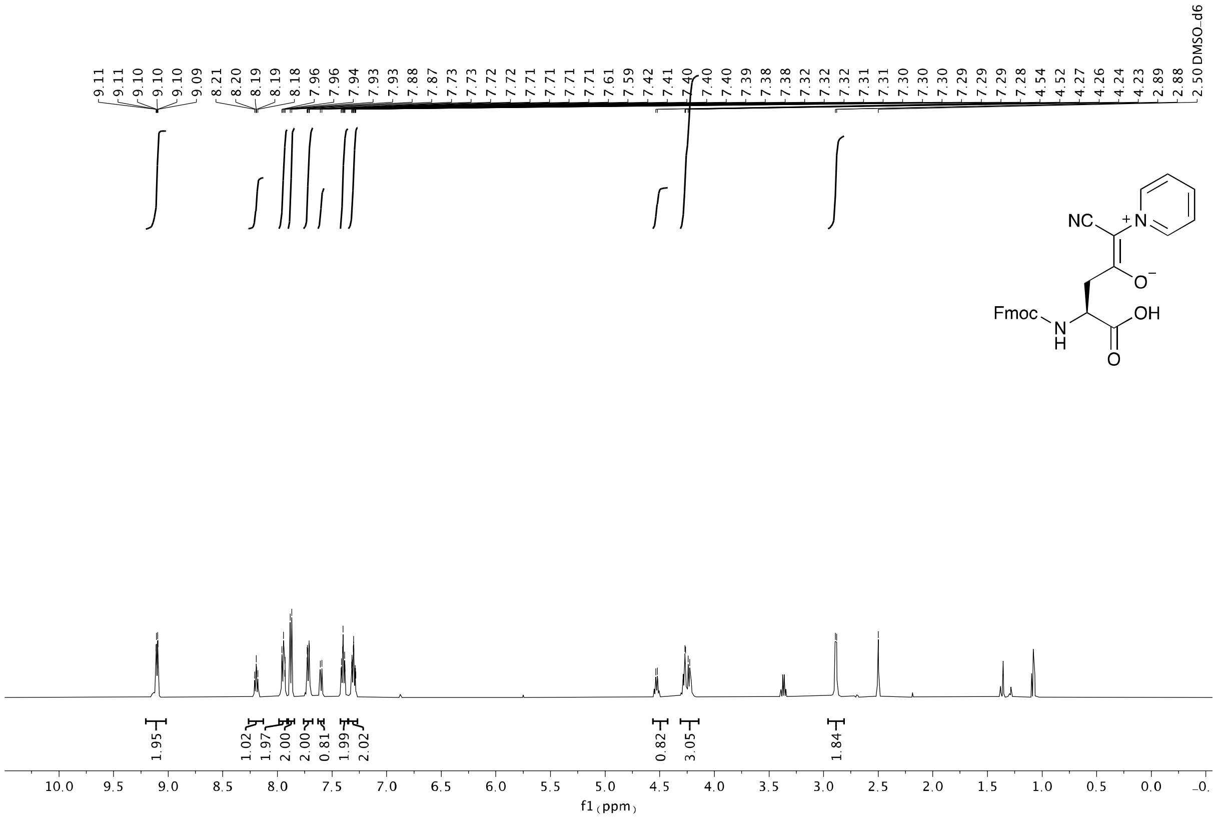

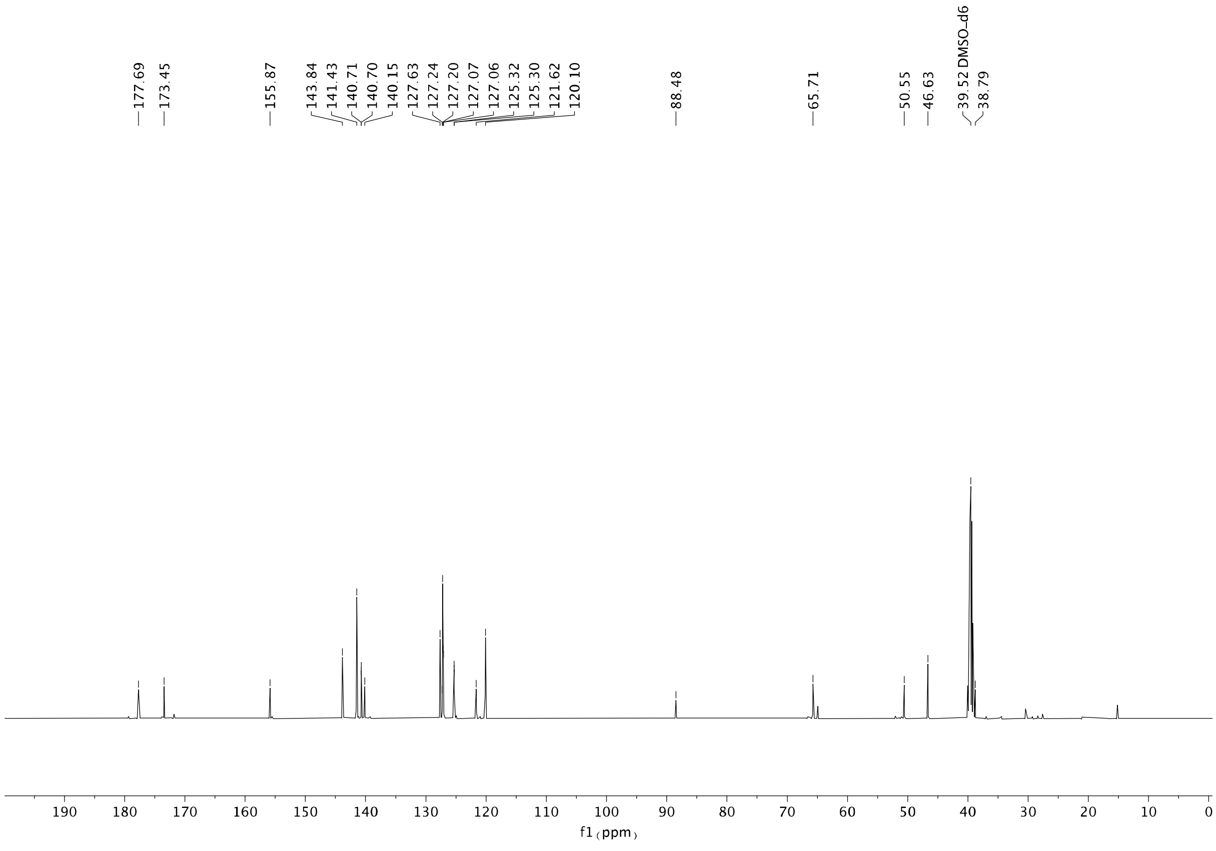

13. HRMS

Hydrocinnamic acid cyanopyridiniumylide 1

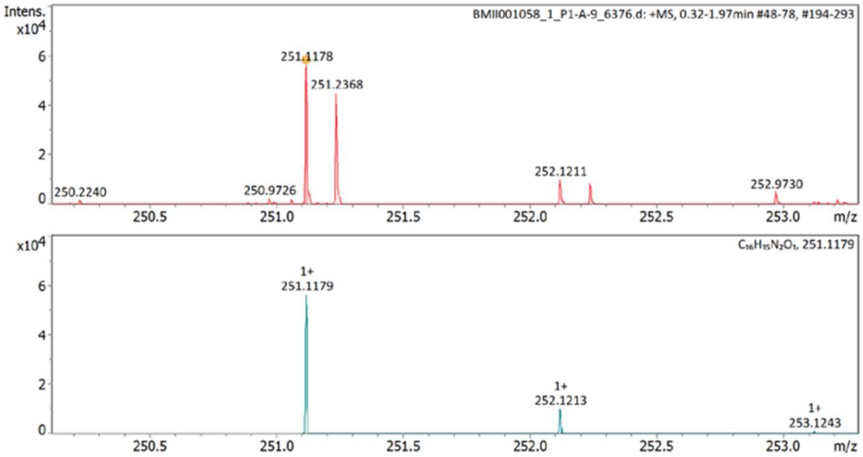

Hydrocinnamic acid cyano(4-methoxy)pyridiniumylide 2

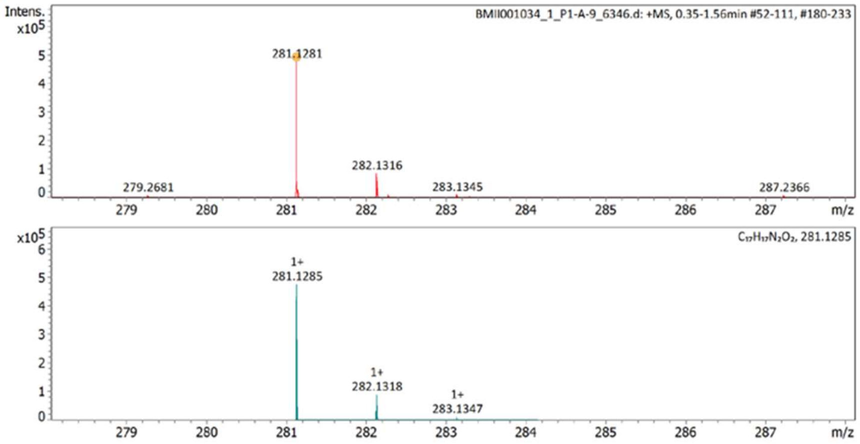

Hydrocinnamic acid cyano(3-(methyl-amide))pyridiniumylide 3

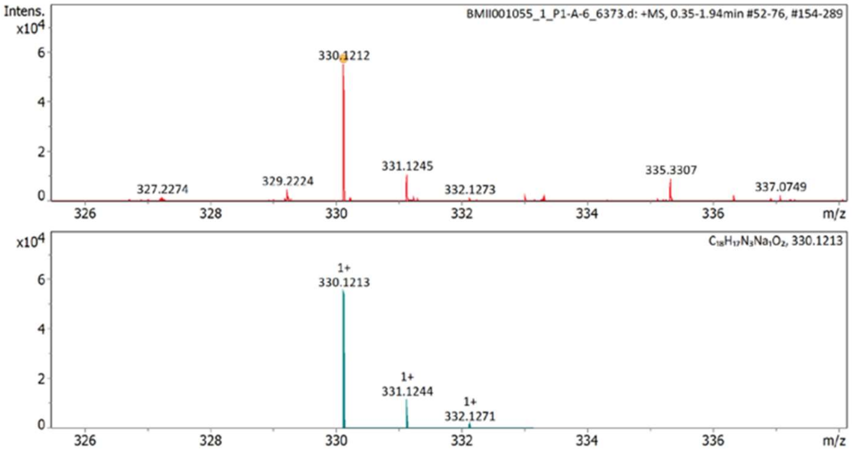

Hydrocinnamic acid cyano(3-chloro)pyridiniumylide 4

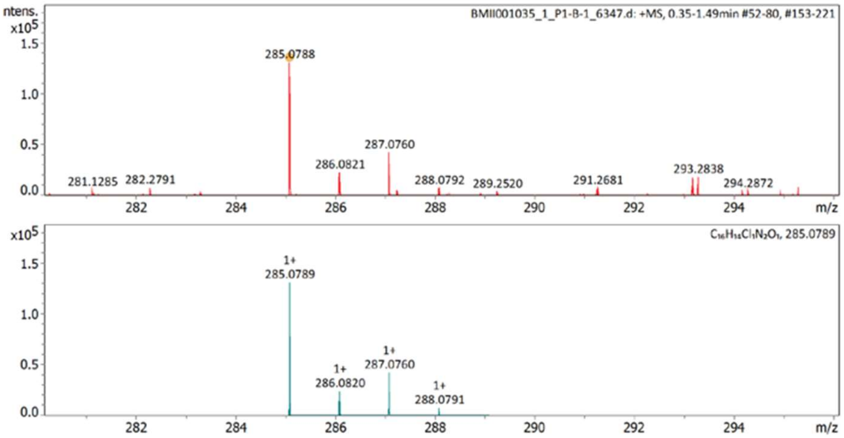

Hydrocinnamic acid cyano(4-phenyl)pyridiniumylide 5

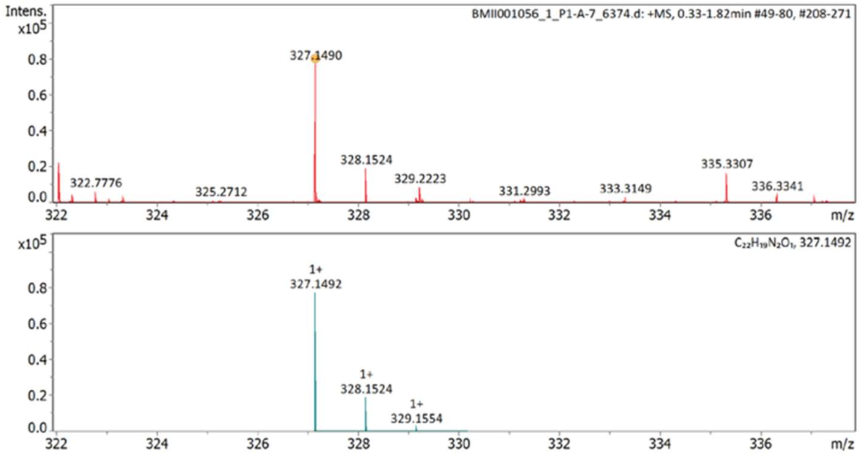

N-acetonitrile pyridinium bromide 7

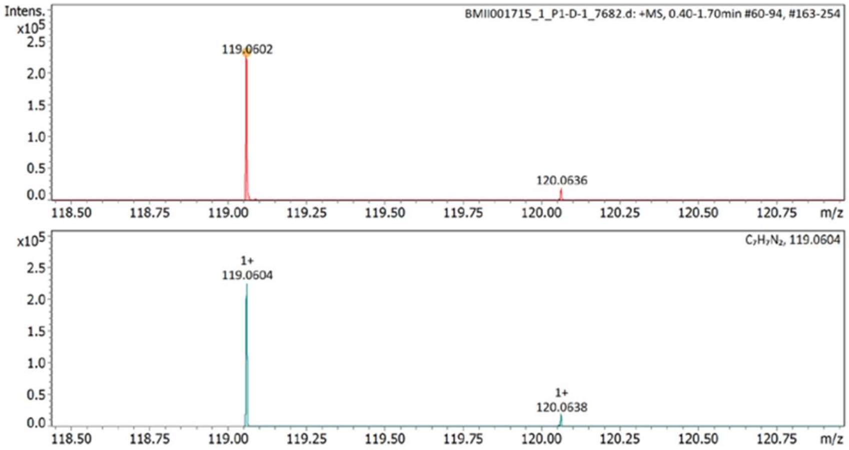

N-acetonitrile 4-Methoxypyridinium bromide 8

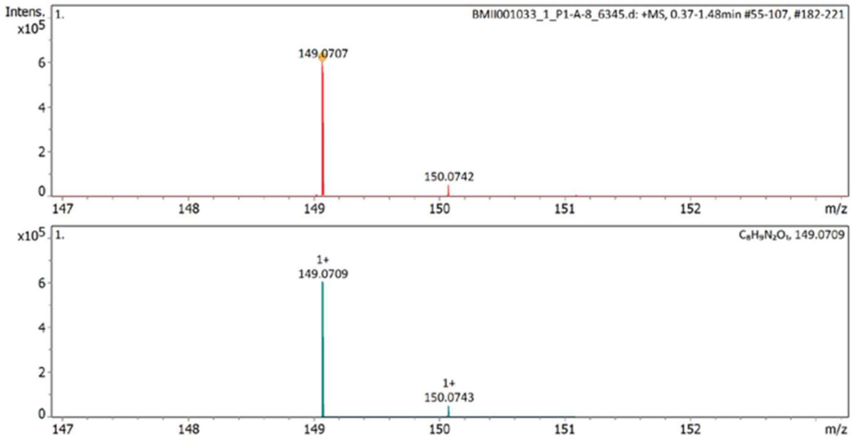

N-acetonitrile (3-methyl)amide-pyridinium bromide 9

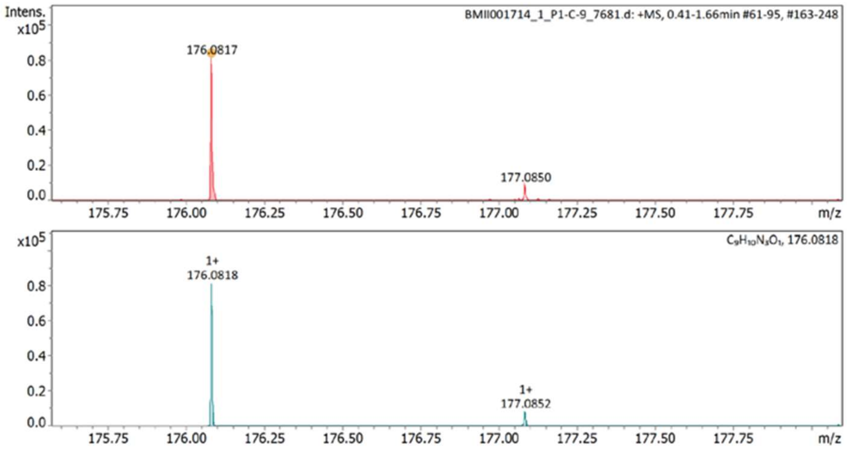

N-acetonitrile 3-Chloropyridinium bromide 10

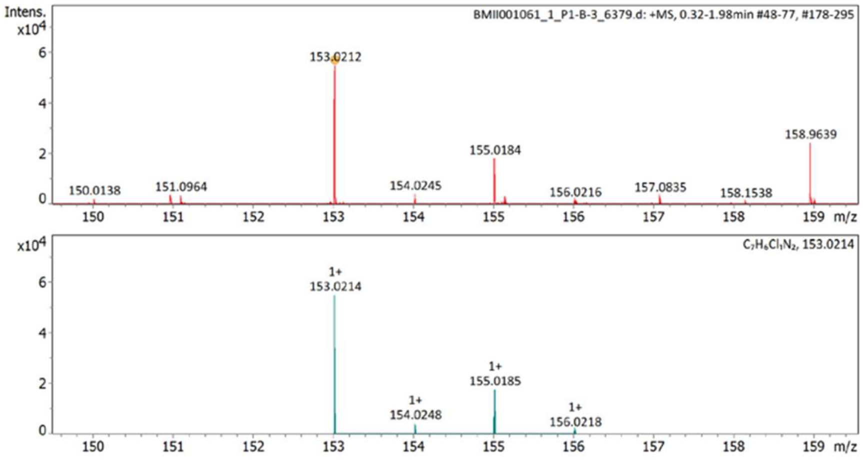

N-acetonitrile 4-phenylpyridinium bromide 11

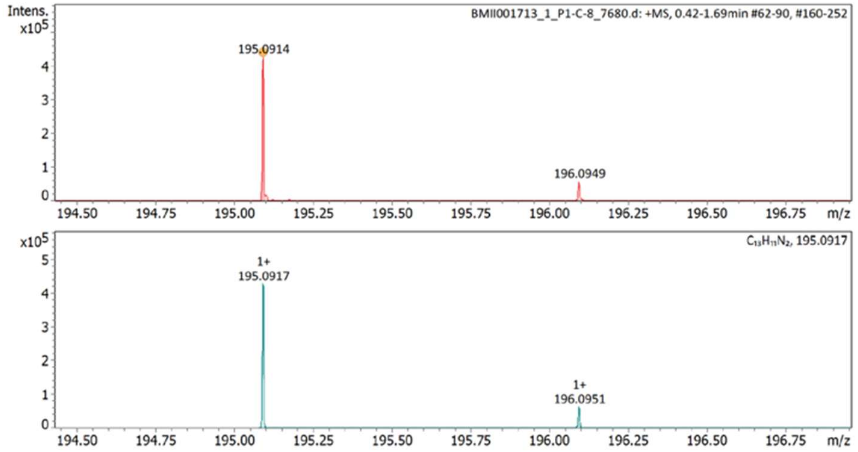

Fmoc-Asp(CyPY)-OH 12

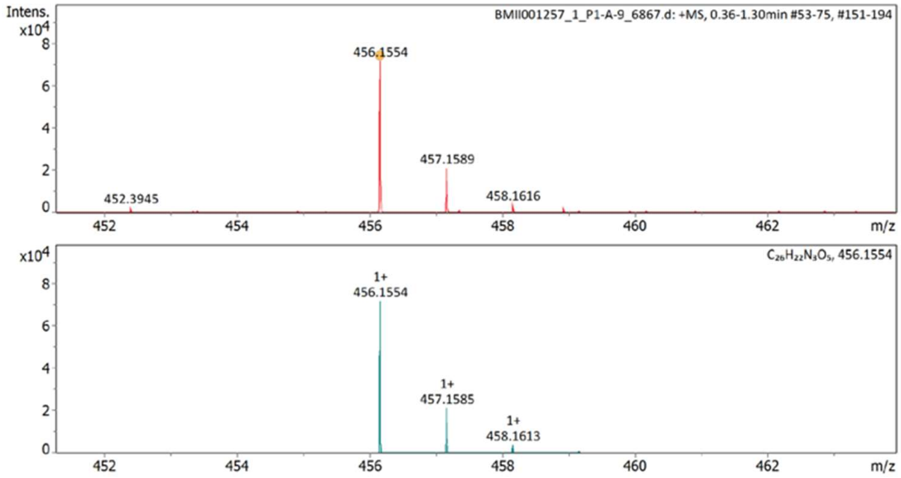

Fmoc-Asp(CyPY)-O^{t}Bu 13

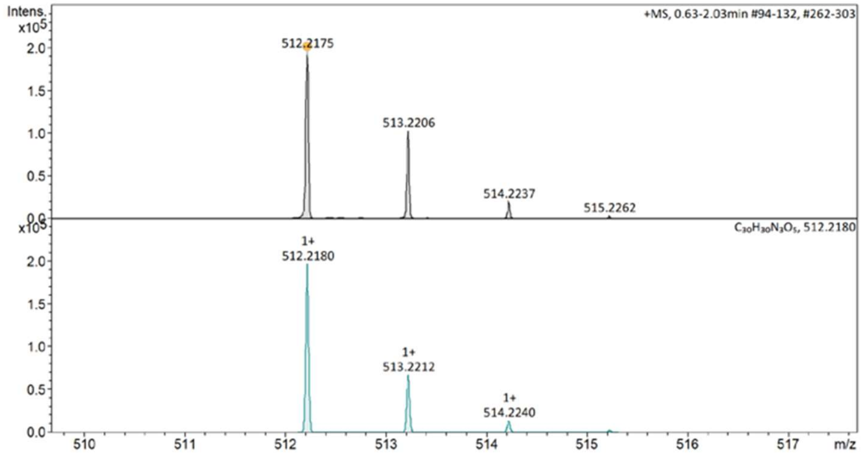

Folded LA3

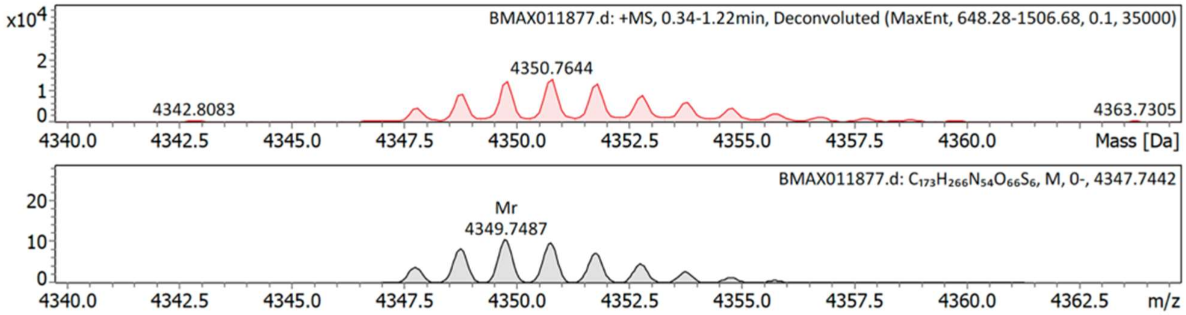

Folded LA4

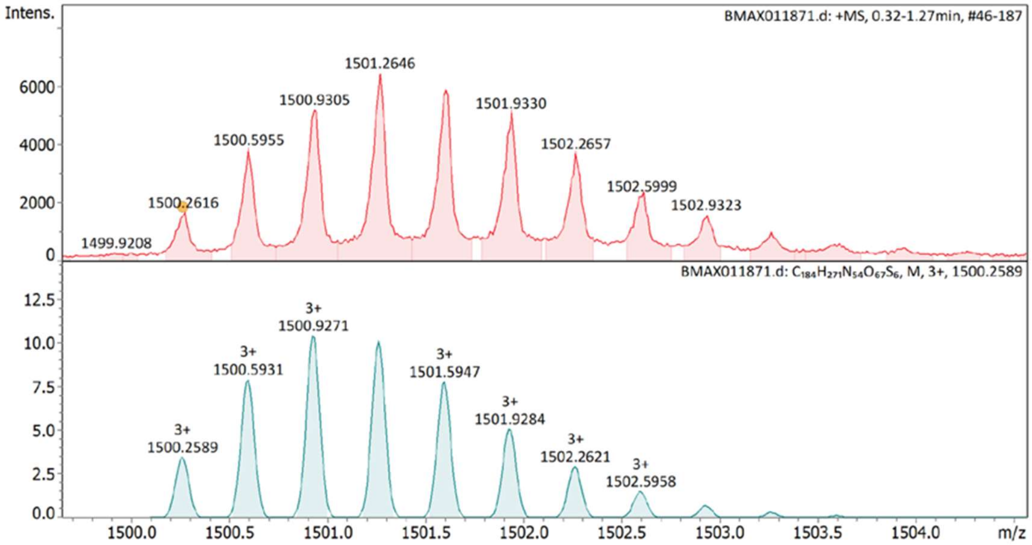

14. MS-Spectra

LA(SS)$_3$-3 module 18

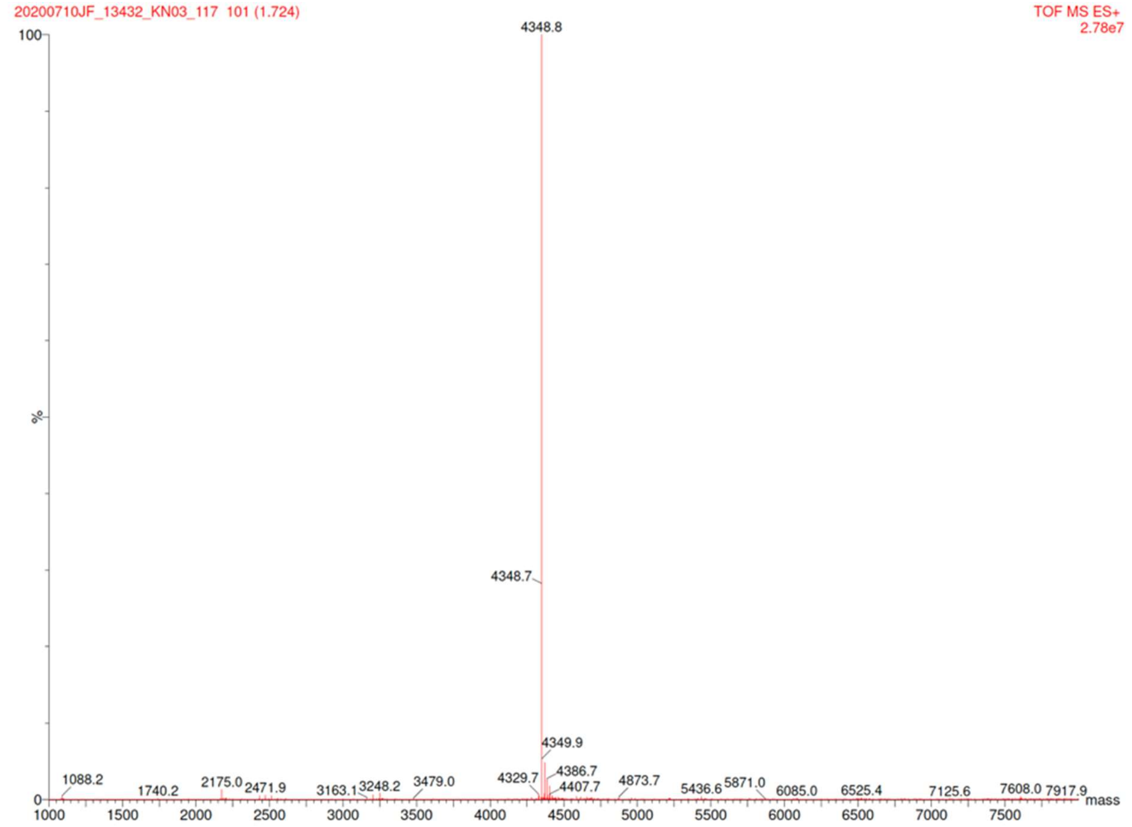

LA(SS)$_3$-4 module 19

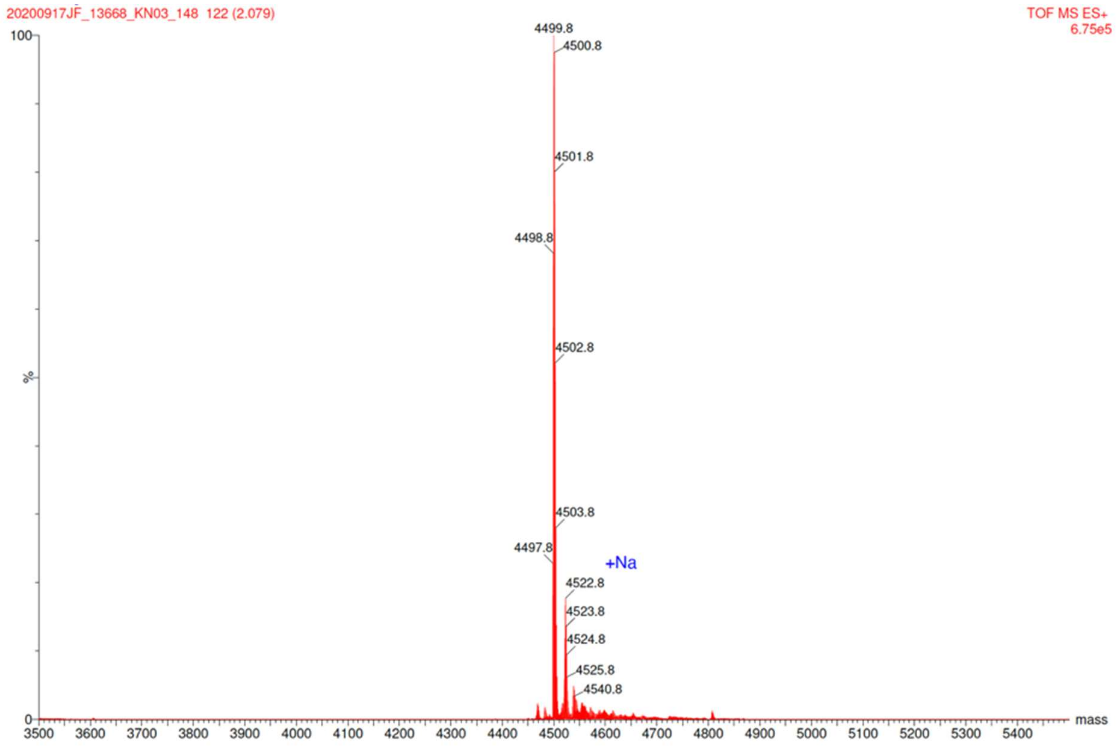

LA(Acm)$_6$-3 module 20

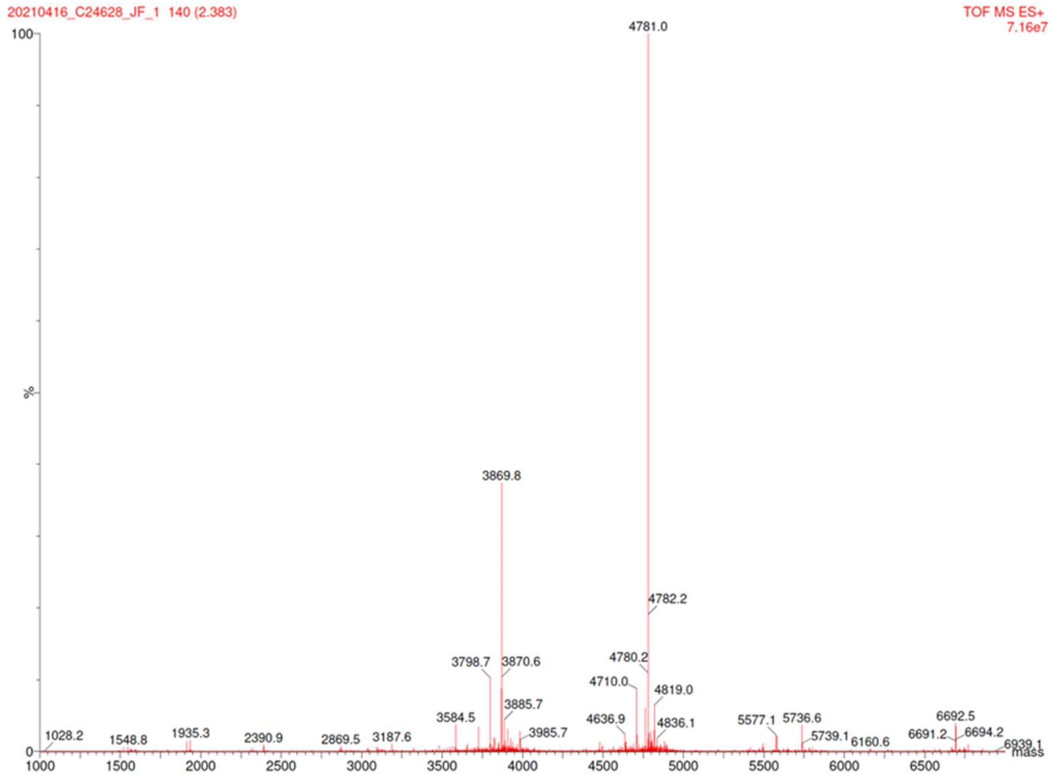

LA(Acm)$_6$-4 module 21

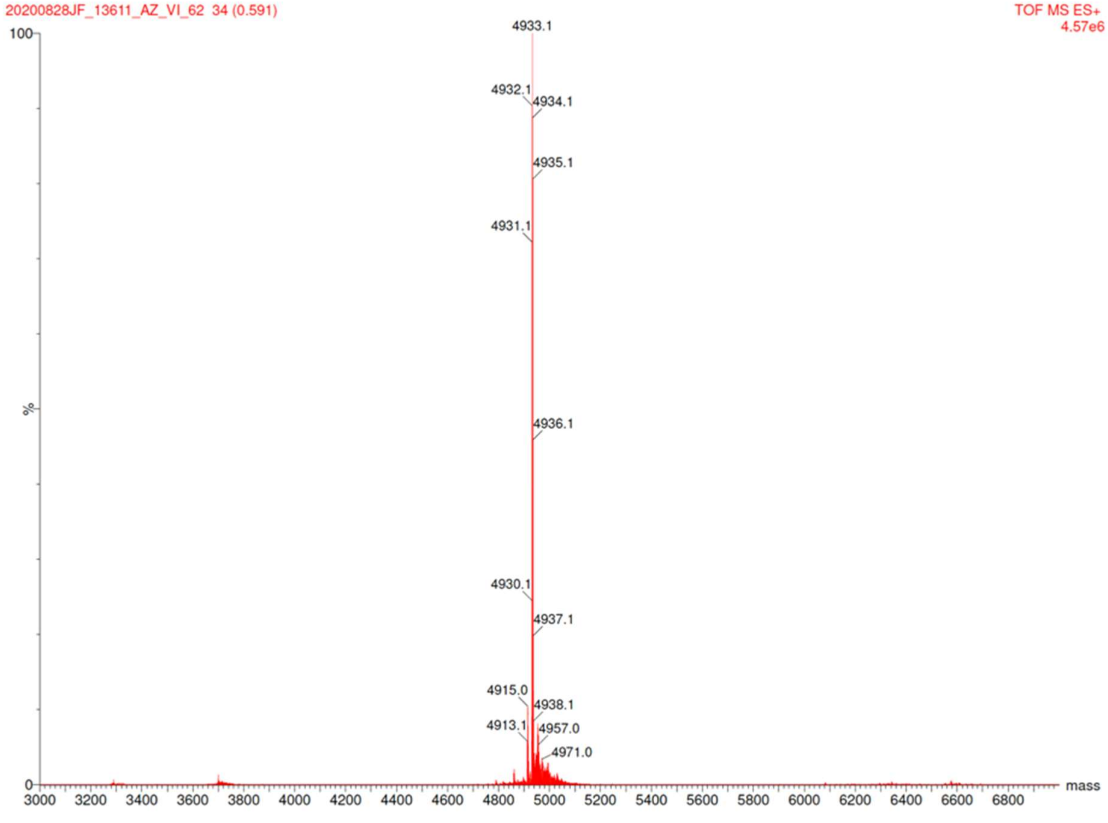

LA(SS)$_6$-3-4 module 24

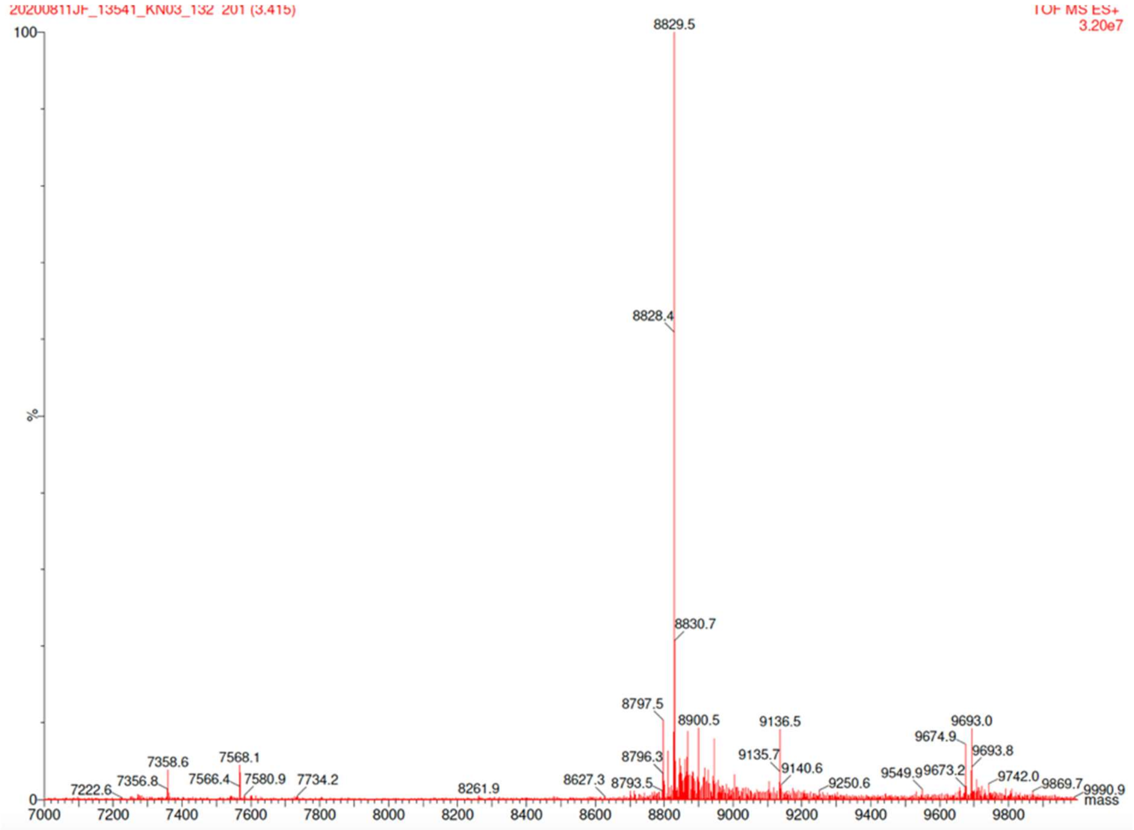

LA(SAcm)$_6$-3 ketoacid module 25

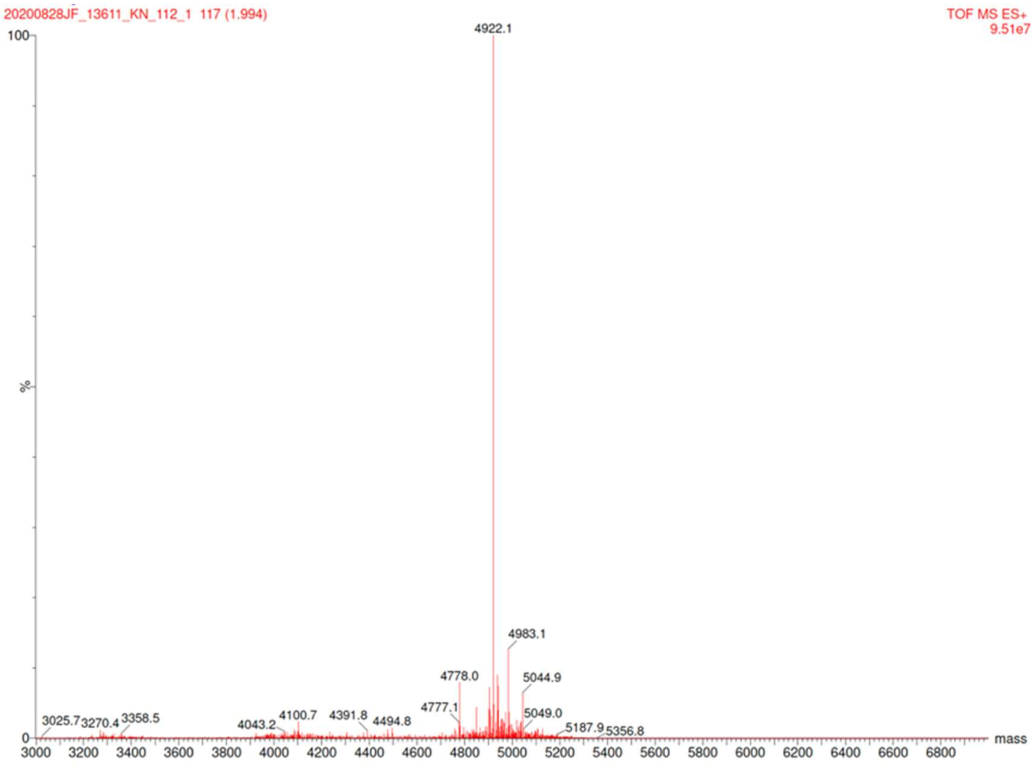

LA(SAcm)$_6$-4 hydroxylamine module 26

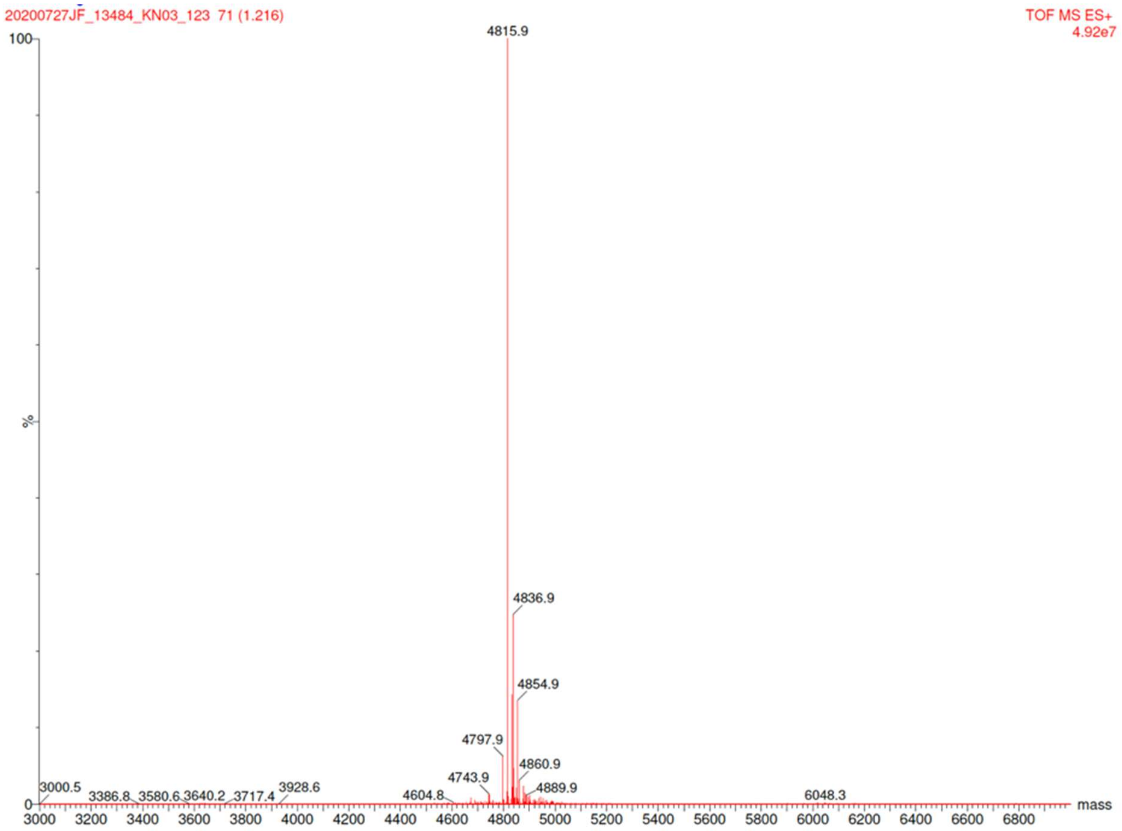

15. Crystallography

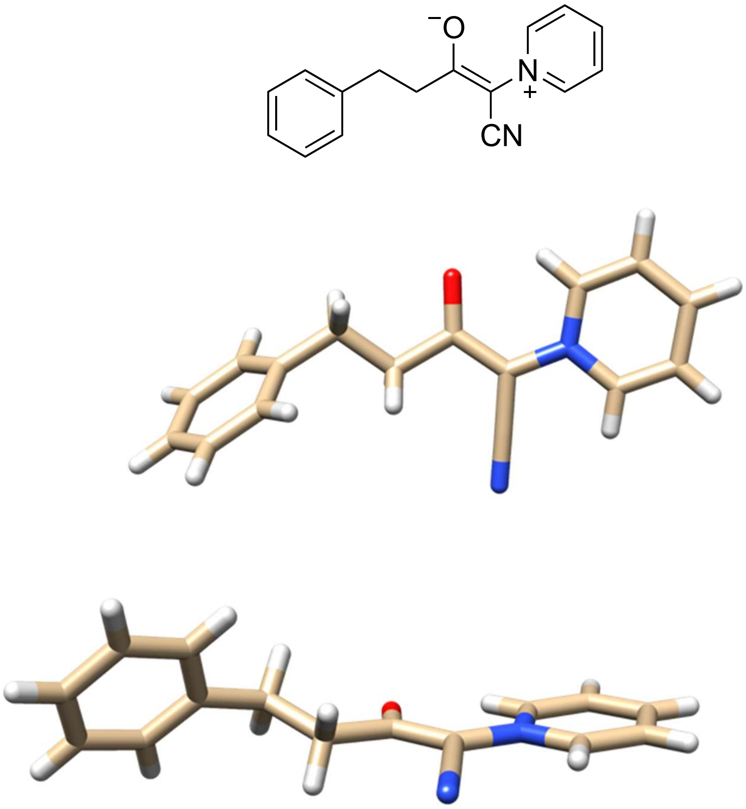

Figure S17 Crystal structures and structure of CyPY **1**.

```
Bond precision:  C-C = 0.0020 A          Wavelength=0.71073

Cell:            a=5.6123(7)       b=8.5149(11)      c=26.949(4)
                 alpha=81.000(4)   beta=87.607(3)    gamma=84.526(3)
Temperature:     100 K

                 Calculated                 Reported
Volume           1265.7(3)                  1265.7(3)
Space group      P -1                       P -1
Hall group       -P 1                       -P 1
Moiety formula   C16 H14 N2 O               C16 H14 N2 O
Sum formula      C16 H14 N2 O               C16 H14 N2 O
Mr               250.29                     250.29
Dx,g cm-3        1.314                      1.313
Z                4                          4
Mu (mm-1)        0.084                      0.084
F000             528.0                      528.0
F000'            528.20
h,k,lmax         7,11,36                    7,11,36
Nref             6383                       6370
Tmin,Tmax        0.991,0.993                0.708,0.746
Tmin'            0.987

Correction method= # Reported T Limits: Tmin=0.708 Tmax=0.746
AbsCorr = MULTI-SCAN

Data completeness= 0.998          Theta(max)= 28.422

R(reflections)= 0.0479( 4331)     wR2(reflections)= 0.1195( 6370)

S = 1.013                  Npar= 343
```

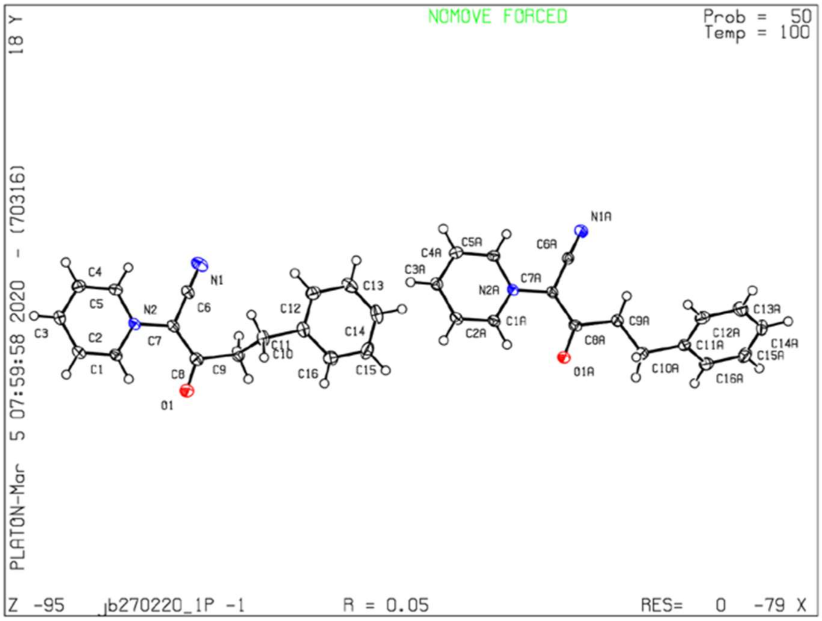

References

1 J. Tailhades, A. Sethi, E. Petrie, P. Gooley, R. Bathgate, J. Wade, M. A. Hossain, Chem. Eur. **2016**, *22*, 1146–1151.

2 F. Thuaud, F. Rohrbacher, A. Zwicky, J. W. Bode *Org. Lett.* **2016**, *18*, 3670–3673.
